# External strain on the plasma membrane is relayed to the endoplasmic reticulum by membrane contact sites and alters cellular energetics

**DOI:** 10.1126/sciadv.ads6132

**Published:** 2025-06-25

**Authors:** Ziming Chen, Peilin Chen, Jiayue Li, Euphemie Landao-Bassonga, John Papadimitriou, Junjie Gao, Delin Liu, Andrew Tai, Jinjin Ma, David Lloyd, Brendan F. Kennedy, Ming Hao Zheng

**Affiliations:** ^1^ Centre for Orthopaedic Research, School of Biomedical Sciences, The University of Western Australia, Nedlands, WA 6009, Australia.; ^2^ BRITElab, Harry Perkins Institute of Medical Research, QEII Medical Centre, Nedlands, and Centre for Medical Research, The University of Western Australia, Perth, WA 6009, Australia.; ^3^ Department of Electrical, Electronic and Computer Engineering, School of Engineering, The University of Western Australia, Nedlands, WA 6009, Australia.; ^4^ Australian Research Council Centre for Personalised Therapeutics Technologies, Melbourne, VIC, Australia.; ^5^ Perron Institute for Neurological and Translational Science, Nedlands, WA 6009, Australia.; ^6^ Department of Orthopaedics, Shanghai Jiao Tong University Affiliated Shanghai Sixth People’s Hospital, Shanghai 200233, PR China.; ^7^ Institute of Future Health, South China University of Technology, Guangzhou International Campus, Guangzhou 511442, PR China.; ^8^ School of Medicine, South China University of Technology, Guangzhou, Guangdong 510006, PR China.; ^9^ Centre of Biomedical and Rehabilitation Engineering, Griffith University, Gold Coast, QLD, Australia.; ^10^ Institute of Physics, Faculty of Physics, Astronomy and Informatics, Nicolaus Copernicus University in Toruń, Grudziadzka 5, 87-100 Torun, Poland.

## Abstract

Mechanotransduction is essential for living cells to adapt to their extracellular environment. However, it is unclear how the biophysical adaptation of intracellular organelles responds to mechanical stress or how these adaptive changes affect cellular homeostasis. Here, using the tendon cell as a mechanosensitive cell type within a bioreactor, we show that the tension of the plasma membrane (PM) and the endoplasmic reticulum (ER) adaptively increases in response to repetitive external stimuli. Depletion of stromal interaction molecule 1 (STIM1), the highest expressed PM-ER tether protein, interfered with mechanotransduction from the PM to the ER, and affected the ER tension. We found that an optimized mechanical strain increased ER tension in a homeostatic manner, but excessive strain resulted in ER expansion, as well as activating ER stress. Last, we showed that changes in ER tension were linked with ER-mitochondria interactions and associated with cellular energetics and function. Together, these findings identify a PM-ER mechanotransduction mechanism that dose-dependently regulates cellular metabolism.

## Teaser

Plasma membrane–endoplasmic reticulum physical tethering is a mechanotransduction pathway.

## INTRODUCTION

By receiving and reacting to repetitive mechanical stimuli, live cells can adapt to their extracellular environment by changing their mechanical properties and cellular behavior (*1*). Mechanotransduction, the ubiquitous process that converts mechanical force into cellular responses, is the key mystery of mechanobiology. It includes the direct transmission of a mechanical force on the plasma membrane (PM) to intracellular components, which is then converted into downstream biochemical signaling responses (*2*). A body of evidence has indicated that dysfunctions in mechanotransduction lead to various diseases, including tendinopathy, osteoporosis, hypertension, asthma, deafness, malaria, and cancer (*3*–*6*). To stabilize cells against mechanical perturbations, the adaptation of cells can be retained even after the cessation of physical stimuli and exposure to a different environment (*7*–*9*). However, the mechanisms contributing to persisting effects of forces on cell activities remain relatively unexplored.

Now, the mechanism by which external mechanical stimuli are relayed to intracellular components, and how this relaying affects downstream cellular responses, is still unclear. Recent studies have yielded notable insights into the processes of intracellular mechanotransmission, but they have been mainly based on the actin cytoskeleton (*10*). Actin cytoskeleton networks are found throughout the cell, providing it a structural advantage for force propagation. In addition to the actin cytoskeleton, the nucleus, the stiffest organelle, also plays a pivotal role in sensing and responding to mechanical stresses (*11*, *12*). Forces transmitted to the nucleus via the cytoskeleton can induce nuclear deformation, chromatin reorganization, and transcriptional changes, establishing a central pathway in mechanotransduction (*13*, *14*). Furthermore, the transcriptional coactivators Yes-associated protein (YAP) and transcriptional coactivator with PDZ-binding motif (TAZ) are also recognized as key mediators of mechanotransduction, linking mechanical signals to essential cellular processes such as proliferation and differentiation (*15*). However, the mechanisms of mechanotransmission in the endomembrane system, another extensive network within the cell, have yet to be elucidated.

The endoplasmic reticulum (ER), one of the largest and generally distributed intracellular organelles, is a continuous structure and the most abundant endomembrane system. It serves as a site for a variety of biological processes, including protein synthesis and folding, sensing of cellular stress, lipid biogenesis, and storage of intracellular calcium (*16*–*18*). Morphologically, the ER is considered as an elaborate system of interconnected tubules with a high membrane curvature and flattened sheets with a low membrane curvature (*19*). The sheet-like ER is mainly involved in protein synthesis, whereas the branched tubular ER is critical for synthesis of lipids and sterols, regulation of calcium storage, and detoxification. Moreover, this extensive membranous network interacts with other cellular components. Mediated by tethering proteins localized to membrane contact sites (MCSs), the ER is physically anchored, although not fused, to many other subcellular structures, including the PM, endosomes, the Golgi complex, lysosomes, lipid droplets, mitochondria, and peroxisomes (*20*). The notable features of structural continuity, wide distribution, and connection to other organelles by the ER mirror those of the cytoskeleton, thereby making this endomembrane compartment a putative platform for transmitting forces from the PM to the cell interior (*21*).

ER-related MCSs are known to regulate several cellular activities. For example, PM-ER contacts are related to activation of store-operated Ca^2+^ entry, autophagosome biogenesis, and lipid transfer (*22*, *23*). Furthermore, ER-mitochondria contacts are known to mediate Ca^2+^ exchange, lipid exchange, mitochondrial division, and autophagosome biogenesis (*24*). In addition, ER-endosome contacts regulate endosome positioning and sterol sensing, cholesterol transfer, and endosomal tubule fission (*25*). However, the role of the ER and physical tethering in dynamic mechanical environments remains unclear.

Mechanical properties of organisms can be adaptively changed in response to mechanical loading or stress (*26*–*29*). Changes in stiffness are attributed to adaptations of the organisms. At a tissue level, clinical studies have suggested that high-loading cyclic strain leads to increased stiffness of tendon (*26*, *27*), which can be detected at the cellular level, as well (*28*). At the subcellular level, it has been shown that even isolated nuclei can resist a force by adjusting their stiffness (*29*). The changes in stiffness, in turn, affect cellular function (*30*). Similar to stiffness for solid objects, membrane tension is the resistance of a membrane to deformation (*31*).

Far from being a passive participant, the tension can act as a regulator of biological processes. Changes in membrane tension are known to regulate membrane morphology, protein assembly, and cellular function (*3*, *32*–*34*). However, detection of tension on subcellular structures is a challenging area in mechanobiology studies, especially in live cells. Traditional techniques for mechanical measurements, such as atomic force microscopy and membrane tether pulling assays (*35*, *36*), are difficult to apply to the measurement of such parameters inside living cells. Thus, little has been explored regarding how the tension on the endomembrane system adaptively responds to repetitive mechanical stimuli and how this, in turn, affects intracellular biological processes.

Here, we aimed to investigate the adaptation of tension in subcellular structures induced by different cyclic strains, the underlying mechanisms of these adaptations, and their associations with cellular function. Tendon cells are an ideal model for the study of mechanobiology as they are effectively dose-dependently mechanosensitive (*37*). Cyclic physiological strain is beneficial for tendon cell function, which can be evaluated by extracellular matrix (ECM) production, whereas pathological strain, including mechanical overload and underload, is harmful (*38*). Here, using tendon cells and in vitro loading models, we reveal a key role of the ER in the propagation of mechanical signals from the PM to the cell interior and the mechanical strain–mediated regulation of cellular energetics and function. Applying fluorescence lifetime imaging microscopy (FLIM) with membrane tension probes and live-cell imaging, we show that membrane tension in the ER adaptively increases after cyclic mechanical strain in a dose-dependent manner. We further show that this response of the ER tension is regulated via a PM-ER tether structure consisting of tether proteins localized to contact sites between the two membranes. We also show how this system relates to ER-mitochondria interaction, which is associated with cellular energy metabolism. Last, by elucidating the underlying mechanisms involved in this process, we can modify PM-ER tethering to rescue cellular function impaired by mechanical overload.

## RESULTS

### Cyclic mechanical strain increases PM and ER tension

**Fig. 1.**
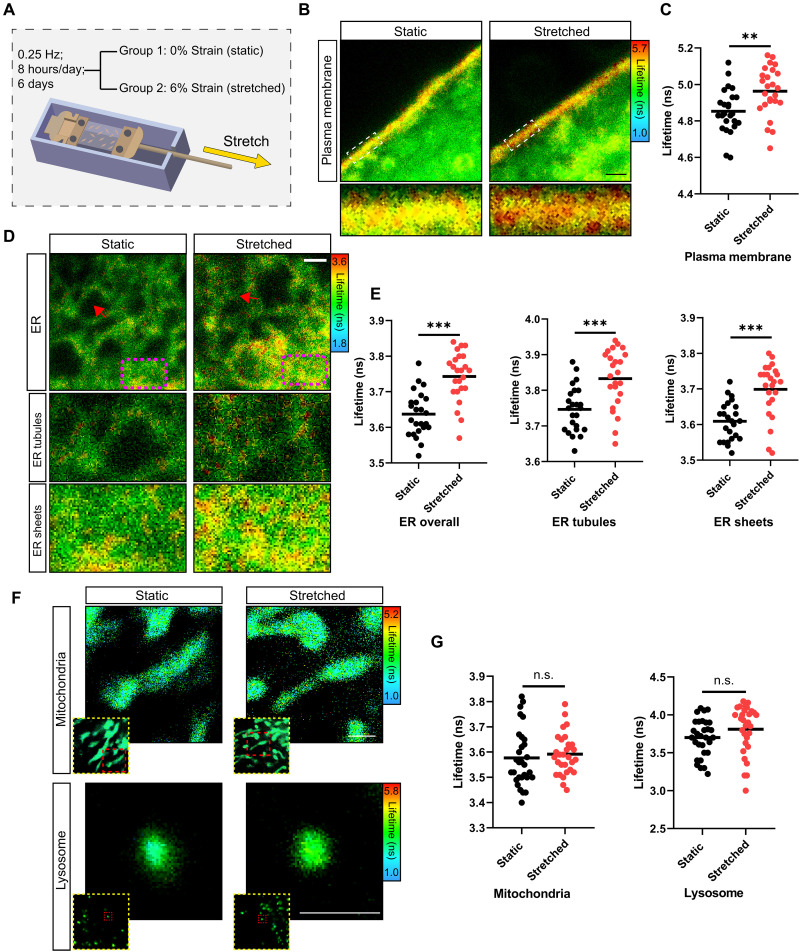
Cyclic mechanical strain increases tension of the PM and the ER. (**A**) Schematic diagram illustrating the experimental approach used to mechanically stimulate monolayers of mouse tendon cells. (**B**) Representative FLIM images of PM tension probed by Flipper-TR in tendon cells with or without cyclic mechanical stimulation. Each bottom panel is an enlargement of the dashed boxed area in the corresponding top panel. (**C**) Distribution of fluorescence lifetime of Flipper-TR after selecting the PM as the region of interest (ROI) between the static group and the stretched group (*n* = 25 cells per group, each with at least two ROIs, from three independent experiments). (**D**) Representative FLIM images of overall ER (top) stained with ER Flipper-TR in tendon cells with or without cyclic mechanical stimulation and their enlargements showing ER tubules (middle), as indicated by the red arrows in the top images, and areas of ER sheets (bottom), as indicated by purple dashed rectangles in the top images. (**E**) Distribution of fluorescence lifetime of ER Flipper-TR selecting the overall ER, ER tubules, or ER sheets, as the ROI between the static group and the stretched group (*n* = 24 cells per group, each with at least two ROIs, from three independent experiments). (**F**) Representative FLIM images of mitochondria stained with Mito Flipper-TR (top) and lysosome stained with Lyso Flipper-TR (bottom), in tendon cells with or without cyclic mechanical strain. Each top right panel is an enlargement of the red dashed boxed area in the yellow dashed boxed bottom left panel. (**G**) Distribution of fluorescence lifetime of Mito Flipper-TR and Lyso Flipper-TR between static group and stretched group (*n* = 30 cells per group, each with at least three ROIs, from three independent experiments). Line marks the mean of the distribution. Scale bars, 1 μm. **P < 0.01; ***P < 0.001; n.s., not significant by Student’s *t* test.

First, we investigated how the tension of subcellular structures changes after cyclic mechanical strain. We used a two-dimensional (2D) uniaxial stretching culture model to induce mechanical stimulation of primary mouse tendon cells, with 6% strain at 0.25 Hz for 8 hours/day for 6 days (*39*) (Fig. 1A). The tension on subcellular structures was detected by mechanosensitive fluorescent lipid tension reporter (FliptR) probes, including Flipper-TR, ER Flipper-TR, Mito Flipper-TR, and Lyso Flipper-TR, which target the PM, the ER, mitochondria, and lysosomes, respectively (*40*, *41*). Use of FliptR probes enabled us to qualitatively and quantitatively measure membrane tension through their fluorescence lifetime depending on the orientation between their chromophoric groups (*40*, *41*). When we examined PM tension by FLIM imaging, we found a longer fluorescence lifetime of Flipper-TR on the PM in cells that underwent stretching compared to cells in static cultures (Fig. 1, B and C). These findings demonstrated that external cyclic mechanical strain could increase the tension on the PM, which was consistent with previous findings (*35*). Tensile stress on cells has been shown to increase the apparent tension of the PM by tether pulling assays (*35*, *42*). However, because such assays inherently deform the membrane, the increased apparent tension of the PM induced by tensile strains could be explained by an inherent PM tension plus the tension induced by the attachment of the PM to the cytoskeleton. In such cases, even if the in-plane tension remains unchanged, stronger attachment of the cytoskeleton to the PM could influence the readout, making it not a measure of “pure” PM tension (*43*). By using the FliptR system, we eliminated any contribution induced by the cytoskeleton attachment to the measurement and further confirmed that cyclic stretch increased the PM in-plane tension.

Next, we examined the tension on the ER, the largest cytoplasmic organelle and one with multiple direct contacts with the PM, by ER Flipper-TR imaging and found a longer fluorescence lifetime in cells that underwent stretching compared to cells in static cultures, indicating that the stimuli resulted in increased ER tension (Fig. 1, D and E). As the ER contains sheets and tubules and these two structures display different membrane tensions (*41*), we evaluated the tension profile of these two distinct ER structures. We found longer fluorescence lifetimes in both ER sheets and ER tubules in mouse tendon cells after stretching, as compared to those in static cultures, indicating that both of these substructures of the ER had a higher tension in response to external mechanical stretch (Fig. 1, D and E). We concluded that ER increasing its ability to resist deformation, thus enhancing its stability under mechanical loading, is a process of cellular mechanoadaptation. This finding is consistent with previously observed instances of mechanoprotection that occurs in response to mechanical stress on certain subcellular structures, including the actin cytoskeleton and the cell nucleus (*28*, *29*, *44*, *45*).

To further map the distribution of tension on the PM and the ER, we costained Flipper-TR and ER Flipper-TR in primary mouse tendon cells. By FLIM imaging, we found that the fluorescence lifetime showed a decreasing gradient from the edge of the cell to its interior, with the highest lifetime at the PM, a lower level at the marginal ER, and the lowest level in the central ER (fig. S1, A and B). These results suggest that changes in the tension of the ER are due to stimuli from the extracellular environment that act on the PM rather than from any direct stimulation on the interior of the cell.

Concurrent elevation of tension on both the PM and the ER after cyclic strain suggests that these structures are perhaps involved in the same mechanotransmission system, bearing the shared stress propagated from one to the other, and therefore exhibit similar adaptation profiles. Next, we examined whether external cyclic mechanical strain also changed the tension in other subcellular organelles, including mitochondria and lysosomes. Using Mito Flipper-TR and Lyso Flipper-TR probes followed by FLIM imaging, we found that there was no significant difference in the membrane tension of mitochondria and lysosomes between cells with and without stretching (Fig. 1, F and G). We increased the strain further to 9% and assessed the tension in mitochondria and lysosomes (fig. S2A). FLIM imaging confirmed no significant differences in the membrane tension of these organelles between static cultures and those subjected to 9% strain (fig. S2, B and C), reinforcing the conclusion that external cyclic mechanical strain does not notably affect the membrane tension of mitochondria and lysosomes.

Next, to evaluate whether cells can acquire adaptive membrane tension after mechanical strain by a short-term stimulation, we investigated the effects of a 1-day cyclic strain on PM tension and ER tension. As a comparison to the 6-day stretching culturing, we first cultured mouse tendon cells with or without 6% strain at 0.25 Hz for 8 hours/day for 1 day, following a 5-day static culturing with the tension measurements taken on the sixth day (fig. S3A). By FLIM imaging analysis, we found that there was no significant difference in the fluorescence lifetime of Flipper-TR on the PM or ER Flipper-TR on the ER in cells subjected to static culturing and after stretching (fig. S3, B to E). To evaluate the possibility of tension recovery after stretching leading to nonsignificant tension changes when measured on the sixth day, we further measured tension after the first day or immediately after the 8-hour stretching (fig. S3, F and K). The fluorescence lifetime of Flipper-TR on the PM or ER Flipper-TR on the ER showed that there was no significant difference between PM tension or ER tension in cells with static cultures and those after stretching, regardless of whether they were assessed after the first day or immediately after the 8-hour stretching (fig. S3, G to J and L to O). These findings indicate that cyclic strain needs to reach a certain threshold of duration to lead to the adaptive changes of PM tension and ER tension.

Together, these results show that cyclic mechanical loading of cells increases PM tension and ER tension but that this response on membrane tension does not universally occur in all cellular organelles.

### ER tension is associated with cellular function and ER stress

Rather than experiencing mechanical forces in 2D, cells in vivo receive such forces in 3D. To explore whether the results we obtained in 2D cultures also apply to 3D cultures, we cultured mouse tendon cells in 3D bioreactors and stimulated them with cyclic mechanical loading (*39*). Our previous ex vivo study showed that cyclic stretching at 0% strain or 3% strain is an underload, whereas 6% strain is a normal load and 9% strain is an overload for tendons (*37*). We therefore performed uniaxial stretch with loading at 0, 3, 6, or 9% strain (0.25 Hz; 8 hours/day; 6 days) on 3D tendon constructs formed by growing mouse tendon cells in vitro in 3D cultures (fig. S4A) (*39*). By immunoblotting for the tenogenesis markers, type 1 collagen (COL1) and tenomodulin (TNMD), as readouts of cell function, we confirmed that the tendon cells in 3D cultures displayed a different functional status with increased strength of loading, with a 6% strain resulting in the greatest degree of cell function compared to underloading (0 and 3% strain) or overloading (9% strain) (fig. S4, B to D).

**Fig. 2.**
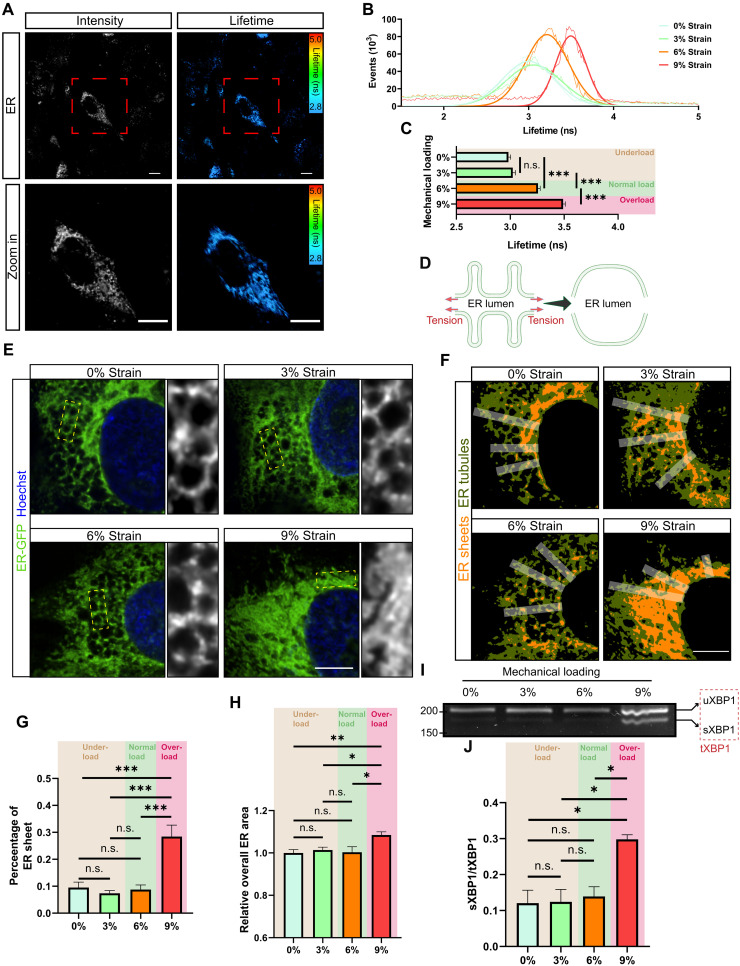
Comparison of ER loading profiles from 3D cell constructs between underloaded, normal loaded, and overloaded conditions. (**A**) Representative confocal and FLIM images of ER Flipper-TR in 3D tendon constructs showing the intensity and corresponding fluorescence lifetime. Scale bars, 10 μm. Bottom panels show the enlargement of the cell in tendon construct as the ROI. (**B**) Representative fluorescence lifetime histograms of ER Flipper-TR with Gaussian fits in 3D tendon constructs receiving 0, 3, 6, or 9% cyclic strain. (**C**) Quantification of fluorescence lifetime of ER Flipper-TR in 3D tendon constructs receiving 0, 3, 6, or 9% cyclic strain (*n* = 15 cells per group from three independent experiments). (**D**) Hypothetical model of tension-driven ER morphological changes. Tensile stress can expand the area of membranes and flatten the membrane at force. (**E**) Representative confocal images of GFP-labeled ER and Hoechst-labeled nuclei in tendon cells receiving 3D mechanical stretching with the indicated loading regimes. (**F**) ER morphology was analyzed by drawing 56-pixel-wide line segments from the nuclear envelope, selecting ER sheets (orange) and tubules (atrovirens) using Renyi entropy threshold, and calculating sheet percentage per segment. (**G** and **H**) Quantification of the percentage of ER sheets (G) and overall ER area (H) in different mechanical loading environments using method (F) (*n* = 15 cells per group from three independent experiments, three regions per cell). (**I** and **J**) RT-PCR analysis (I) and its quantification (J) of XBP1 splicing with different mechanical loading regimes (three biological replicates from three independent experiments). The size of unspliced XBP1 (uXBP1) is 205 base pairs (bp), whereas spliced XBP1 (sXBP1) is 179 bp. Total XBP1 (tXBP1) was defined as uXBP1 + sXBP1. Scale bars, 5 μm. *P < 0.05; **P < 0.01; ***P < 0.001; n.s., not significant by one-way ANOVA. Error bars stand for SEM.

We next examined the ER tension profile of the tendon constructs in the 3D system and found that the fluorescence lifetime of ER Flipper-TR positively correlated with an increase in the levels of mechanical strain, although there was no significant difference between 0% strain and 3% strain (Fig. 2, A to C). These results, in addition to the findings above that show there was no difference in cell function between 0 and 3% strain (fig. S3, B to D), imply that external mechanical signals can be relayed to the ER in this system in a dose-dependent manner, but with a certain threshold level needed to affect ER tension and further to cell function.

Tensile stress can expand the area of the membrane and flatten it at force (*32*, *33*, *46*). Thus, we assumed that the cyclic strain–induced changes in ER tension would also alter the ER morphology (Fig. 2D). We thus measured the shape of the ER by labeling the organelle with green fluorescent protein (GFP) fused to the ER signal sequence of calreticulin and KDEL followed by confocal microscopy. We found that 9% cyclic strain altered ER shape and led to ER expansion (Fig. 2E). However, 6% cyclic loading did not cause obvious changes in ER morphology (Fig. 2E), although as noted above, it was sufficient to increase the ER tension. Measurement of the ratio of ER sheets to ER tubules showed that the percentage of ER sheets significantly increased in cells from tendon constructs receiving 9% strain as compared to those receiving no more than 6% strain (Fig. 2, F to H). These results suggest that only excessive external strain, and thus hyperelevated ER tension, is associated with altered morphological features of the ER.

A shift of the ER configuration from mostly tubules to sheets is a way of expanding ER content (*47*) (Fig. 2G). However, ER expansion is normally related to ER stress and dysfunction, leading to the buildup of excessive unfolded proteins (*47*, *48*). Such accumulation results in induction of unconventional splicing of X-box binding protein 1 (XBP1) mRNA (*49*). Thus, we speculated that the morphological changes of ER at 9% strain result in ER stress and induction of the unfolded protein response. We performed semiquantitative reverse transcription polymerase chain reaction (RT-PCR) for *Xpb1* and found that the ratio of spliced *Xbp1* to total *Xbp1* increased in tendon constructs receiving 9% strain compared to those receiving 0, 3, or 6% strain (Fig. 2, I and J). Furthermore, live-cell confocal imaging using thioflavin T (ThT), a small molecule capable of detecting ER stress by exhibiting enhanced fluorescence when it binds to protein aggregates (*50*), showed increased fluorescence intensity of ThT in tendon constructs receiving 9% strain compared to those receiving 0, 3, or 6% strain (fig. S5, A and B), further implying that excessive strain to tendon constructs causes ER stress. There was no significant difference among tendon constructs receiving 0, 3, or 6% strain with respect to the ratio of spliced *Xbp1* to total *Xbp1* or the fluorescence intensity of ThT.

Together, our results indicate that normal cyclic loading with 6% strain alters ER tension but does not cause ER expansion. Overloading at 9% strain causes changes in ER morphology and induces ER expansion while activating ER stress.

### Altering PM-ER tethering changes ER tension

**Fig. 3.**
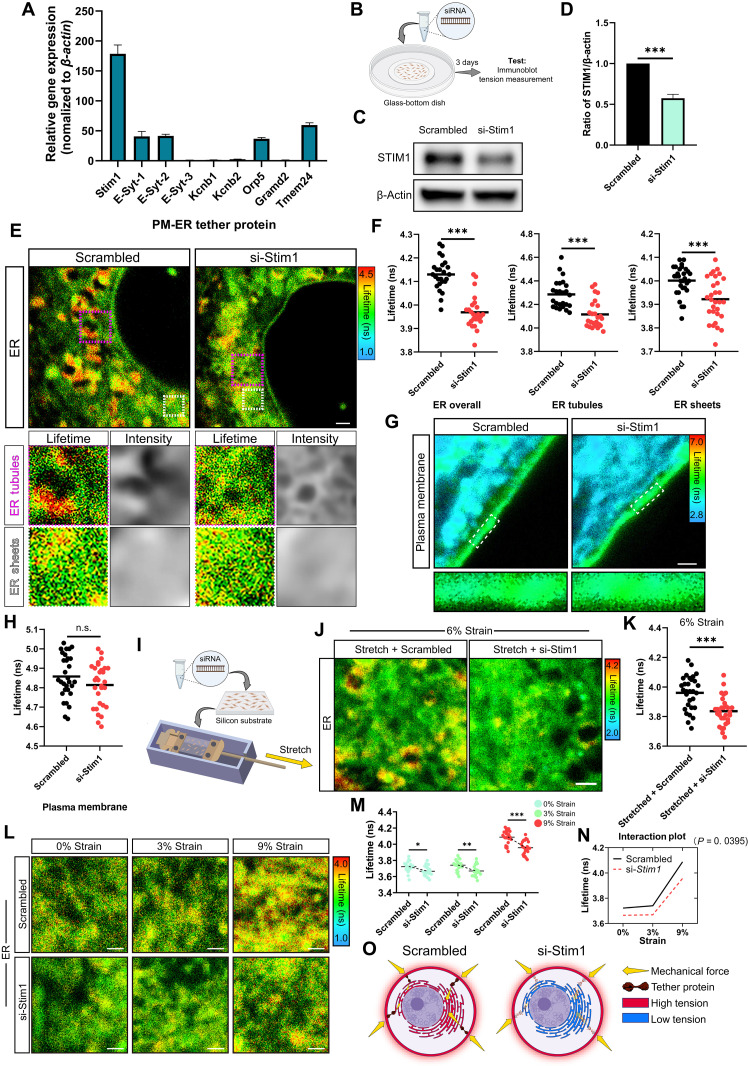
Partial knockdown of the PM-ER tether protein STIM1 reduces ER tension. (**A**) RT-qPCR analysis of PM-ER tether proteins in mouse tendon cells (three biological replicates from three independent experiments). (**B**) Schematic diagram illustrating the experimental approach used to test membrane tension of tendon cells transfected with scrambled siRNA or *Stim1*-siRNA. (**C** and **D**) Immunoblot analysis (C) and quantification (D) of STIM1 expression in cells 3 days post-siRNA transfection (three biological replicates from three independent experiments). (**E** and **F**) Representative FLIM images (E) of ER Flipper-TR in cells transfected with siRNA (top). Bottom panels show enlarged views of ER tubules (purple dashed rectangles) and ER sheets (white dashed rectangles) from the top panels, alongside confocal images displaying intensity, and quantification (F) selecting the overall ER, ER tubules, or ER sheets (*n* = 25 to 30), as the ROI. (**G** and **H**) Representative FLIM images (G) of Flipper-TR in cells transfected with siRNA and quantification (H) selecting the PM as the ROI (*n* = 30). (**I**) Schematic illustrating 2D mechanical stimulation to tendon cells transfected with siRNA. (**J** and **K**) Representative FLIM images of ER Flipper-TR in cells transfected with siRNA after 2D 6% cyclic strain (J) and quantification (K) (*n* = 30). (**L** and **M**) Representative FLIM images of ER Flipper-TR in tendon cells transfected with siRNA after 2D 0, 3, or 9% cyclic strain (L) and quantification (M) (*n* = 21 to 24). (**N**) Interaction plot from two-way ANOVA indicating the interactive effect of partial *Stim1* knockdown and cyclic strain on ER tension. (**O**) Schematic illustrating that detethering the ER from the PM disrupts mechanical force propagation, reducing adaptive ER tension. Scale bars, 1 μm. *n*, cells per group, each with at least two ROIs, from three independent experiments. *P < 0.05; **P < 0.01; ***P < 0.001; n.s., not significant by Student’s *t* test (D, F, H, and K) or two-way ANOVA with Sidak’s post hoc (M and N). Error bars stand for SEM. (B), (I), and (O) created with BioRender.com.

Next, we focused on the mechanism regulating ER tension. As the ER is physically tethered to the PM via MCSs, we hypothesized that PM-ER tether proteins could act as mechanotransmitters and induce the adaptive changes in membrane tension of the ER. We selectively labeled PM-ER junctions by expressing the marker GFP-membrane attached peripheral ER (MAPPER) (*51*) and confirmed the existence of PM-ER contacts in mouse tendon cells (fig. S6, A and B). Furthermore, we measured the gene expression of known ER-localized PM-ER tether proteins (*52*–*57*) in mouse tendon cells by reverse transcription with quantitative polymerase chain reaction (RT-qPCR) and found that stromal interaction molecule 1 (*Stim1*) was by far the most highly expressed (Fig. 3A). As knockdown of *Stim1* expression has been shown to reduce the degree of PM-ER contacts (*56*), we suppressed *Stim1* expression by small interfering RNA (siRNA) in unstimulated mouse tendon cells to investigate the role of PM-ER contacts in mechanotransduction (Fig. 3, B to D). We found that the fluorescence lifetime of ER Flipper-TR in total ER, ER tubules, and ER sheets was lower after partial *Stim1* knockdown compared to tendon cells transfected with scrambled siRNA (Fig. 3, E and F). By use of the Flipper-TR probe, we found that these changes in ER tension occurred independently of any changes in the tension of the PM as there was no difference in the fluorescence lifetime of the PM probe between cells transfected with scrambled siRNA or with *Stim1* siRNA (Fig. 3, G and H). Together, these results showed that STIM1 mediates the induction of adaptive ER tension.

To further confirm the effect of partial *Stim1* knockdown on ER tension, we next transfected mouse tendon cells with *Stim1* siRNA in 2D uniaxial stretching culture with 6% strain (Fig. 3I and fig. S7). By probing with ER Flipper-TR, we found that compared to the scrambled control cells, partial *Stim1* knockdown also resulted in lower fluorescence lifetime in the ER after stretching, indicating that the ER tension of cells receiving strain is also reduced by si-*Stim1* treatment (Fig. 3, J and K). We further evaluated the effects of partial *Stim1* knockdown on tendon cells subjected to 0, 3, and 9% strain. Using FLIM imaging, we found that partial knockdown of *Stim1* efficiently reduced ER tension in tendon cells under all conditions, whether subjected to 0, 3, or 9% strain (Fig. 3, L and M). However, interaction analysis revealed that the extent of ER tension reduction following si-*Stim1* treatment differed significantly depending on the applied strain (Fig. 3N). Specifically, the reduction in ER tension was greater in cells subjected to 9% strain compared to those under 0 or 3% strain. These findings further reinforced the conclusion that PM-ER contact sites served as a regulator for increasing ER tension in response to cyclic strain. We thus propose that detethering of the ER from the PM by si-*Stim1* treatment hinders the propagation of mechanical stress from the PM to the ER and thus prevents the adaptive changes of ER tension (Fig. 3O).

**Fig. 4.**
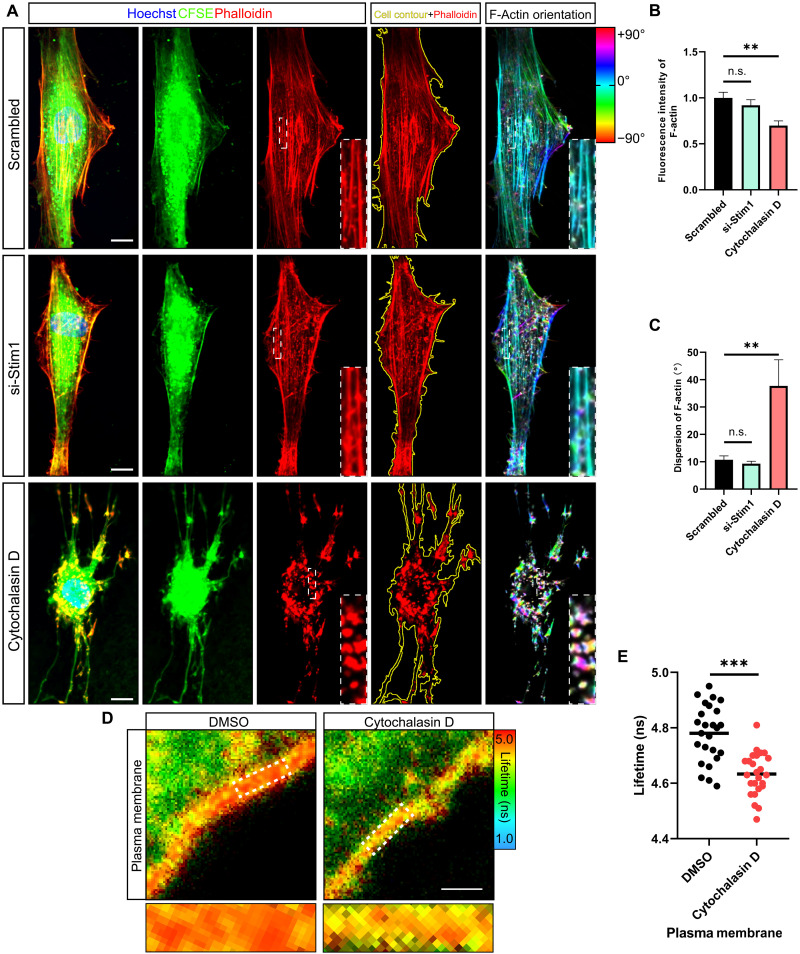
Regulating the PM-ER tethered mechanotransmission system can manipulate the ER tension without interfering the actin cytoskeleton. (**A**) Representative confocal images and analysis of CFSE-labeled cells (green), phalloidin-labeled F-actin (red), and merged images with Hoechst-labeled nuclei (blue) in tendon cells transfected with either scrambled siRNA or *Stim1*-siRNA or treated with cytochalasin D. For image analysis, the cell contour (yellow) is indicated by CFSE, and the F-actin orientation is color coded (rightmost images). More highly magnified images in the phalloidin-stained cells (middle) and the color-coded F-actin orientation indicated that cells (rightmost) are of the white dashed boxed areas. Scale bars, 10 μm. (**B**) Fluorescence intensity quantification by analyzing phalloidin fluorescence intensity divided by cell area (*n* = 15 cells per group from three independent experiments). (**C**) Dispersion quantification of F-actin by analyzing F-actin orientation (*n* = 15 cells per group from three independent experiments). (**D**) Representative FLIM images of PM tension probe Flipper-TR–stained tendon cells treated with carrier [dimethyl sulfoxide (DMSO)] or with cytochalasin D. Scale bar, 1 μm. (**E**) Distribution of the fluorescence lifetime of Flipper-TR selecting the PM as the ROI in tendon cell treated with or without cytochalasin D (*n* = 25 cells per group, each with at least two ROIs, from three independent experiments). **P < 0.01; ***P < 0.001; n.s., not significant by Student’s *t* test (B and C) or by one-way ANOVA (E). Error bars stand for SEM.

Canonically, mechanotransduction from the extracellular environment into cells occurs via actin filaments (F-actin) (*58*). This classic pathway is commonly used to inhibit intracellular mechanotransduction, but it also has limited application as disruption of the cytoskeleton affects many aspects of cellular activity (*59*, *60*). To test whether PM-ER tethered mechanotransduction is dependent on cytoskeleton mechanotransduction, we first assessed whether detethering the PM from the ER affected the cytoskeleton. We used cytochalasin D (20 μM) to disrupt the cytoskeleton as a positive control (*61*). By labeling the cytoskeleton with phalloidin and labeling cells with carboxyfluorescein diacetate succinimidyl ester (CFSE), followed by confocal fluorescence microscopy, we found that cells with a disrupted cytoskeleton induced by cytochalasin D displayed less F-actin fluorescence intensity and higher dispersion of F-actin orientation, whereas there was no significant difference of F-actin fluorescence intensity and F-actin orientation between cells transfected with scrambled siRNA or si-*Stim1* (Fig. 4, A to C). These results indicated that si-*Stim1* treatment does not notably disrupt the cytoskeleton. Next, by probing with Flipper-TR, we found that cytochalasin D treatment did reduce the fluorescence lifetime of the probe in the PM, indicating that disruption of the cytoskeleton decreases the tension of the PM (Fig. 4, D and E), which was not shown in cells after si-*Stim1* treatment (Fig. 3, G and H). Furthermore, using the ER Flipper-TR probe, we observed that cytochalasin D treatment reduced the fluorescence lifetime of the probe in the ER, indicating that disruption of the cytoskeleton decreased ER tension (fig. S8, A and B). This finding suggests that the cytoskeleton is essential for maintaining the tension of both the ER and the PM. In addition, we extended our investigation to examine the role of the actin cytoskeleton in si-*Stim1*–treated cells cultured for 6 days under 9% strain using cytochalasin D, comparing it to the baseline ER tension in control cells under static culture (fig. S9A). Confocal microscopy confirmed the depolymerization of F-actin in cells treated with cytochalasin D under cyclic strain (fig. S9B). FLIM imaging analysis further demonstrated that, under 9% strain, the combined disruption of the actin cytoskeleton and si-*Stim1* transfection resulted in ER tension comparable to that of control cells under the static condition (fig. S9, C and D). As si-*Stim1* treatment reduced ER tension under static culture conditions to levels lower than those in control cells (Fig. 3, E and F), this suggests that external cyclic forces may still influence ER tension, potentially through remaining mechanotransduction pathways, such as residual PM-ER contact sites or other yet-to-be-identified structures (fig. S10, A to D).

Together, our results indicate that, although the actin cytoskeleton is essential for maintaining the tension of both the PM and the ER, changes of the PM-ER tethering structure can alter the ER tension without notably affecting the actin cytoskeleton.

### STIM1 mediates ER tension via tethering, not store-operated calcium entry

**Fig. 5.**
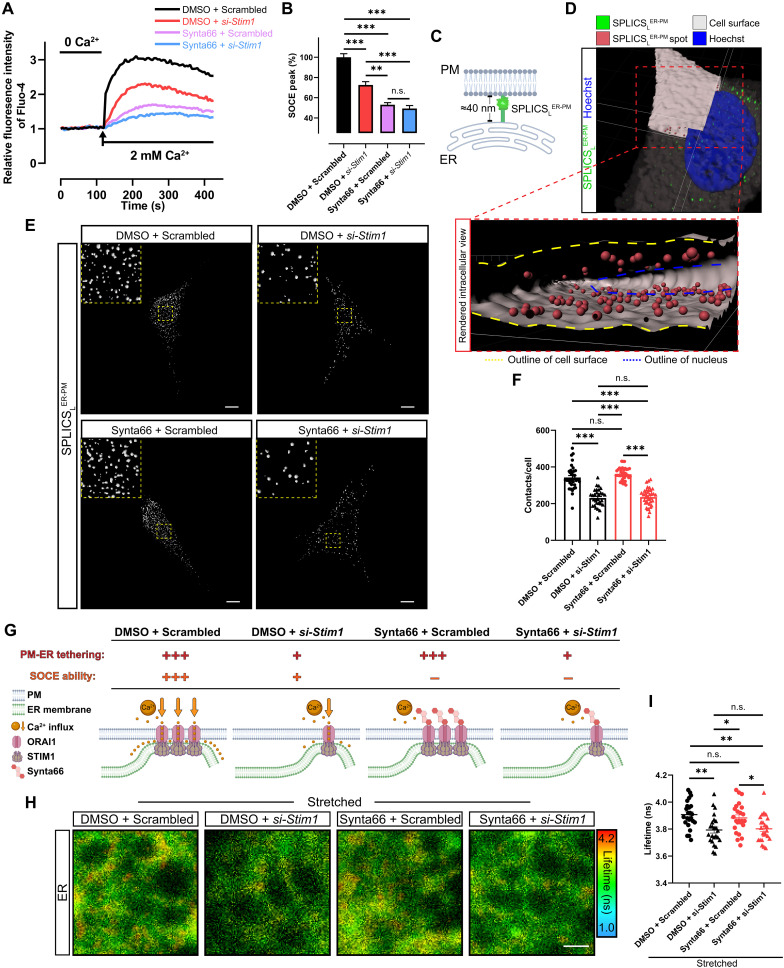
STIM1 mediates ER tension via the physical PM-ER tethering rather than SOCE. (**A**) Representative time course of cytosolic Ca^2+^ measurements in Fluo-4 NW–loaded tendon cells transfected with scrambled siRNA or *Stim1*-siRNA and further treated with DMSO (carrier) or Synta66 for 6 days. (**B**) Normalized maximal values of SOCE from tendon cells transfected with siRNA and further treated with DMSO or Synta66 for 6 days (five biological replicates, three independent experiments). (**C**) Cartoon showing the PM-ER contact sites measured by SPLICS_L_^ER-PM^ that can detect interactions occurring between the PM and the ER within a reciprocal distance of around 40 nm. (**D**) Representative 3D-rendered confocal image of tendon cells expressing SPLICS_L_^ER-PM^. SPLICS_L_^ER-PM^ spots were rendered by detecting SPLICS_L_^ER-PM^ signals. Rendered intracellular view (bottom) is an enlargement of the red dashed boxed area in the top panel, showing SPLICS_L_^ER-PM^ spot-labeled PM-ER contact sites. (**E** and **F**) Representative 3D-rendered confocal images of tendon cells expressing SPLICS_L_^ER-PM^ and transfected with siRNA, further treated with DMSO or Synta66 for 6 days (E) and quantification (F) (*n* = 31 to 37 cells per group from three independent experiments). Each top left panel is an enlargement of the yellow dashed boxed area in the corresponding middle panel. Scale bars, 10 μm. (**G**) Schematics summarizing the status of PM-ER tethering and the SOCE ability in tendon cells with different treatments. (**H** and **I**) Representative FLIM images (H) of ER Flipper-TR in tendon cells transfected with siRNA and treated with DMSO or Synta66 in 2D cyclic stretching environment for 6 days and quantification (I) (*n* = 24 cells per group, each with at least two ROIs, from three independent experiments). Scale bars, 1 μm. *P < 0.05; **P < 0.01; ***P < 0.001; n.s., not significant by one-way ANOVA (B and I) or Kruskal-Wallis test (F). Error bars stand for SEM. (C) and (G) created with BioRender.com.

More than just being a physical tether protein to act as a mechanotransmitter between the PM and the ER, STIM1 is also essential for calcium homeostasis. STIM1 can sense the concentration of Ca^2+^ within the ER. Ca^2+^ store depletion increases the number of STIM1 dimers within each MCS (*62*). STIM1 dimers then engage with ORAI1, a PM Ca^2+^ channel. STIM1 binding with all six subunits of ORAI1 leads to the fully opening of the channel’s pore, allowing Ca^2+^ to enter the cell via the store-operated calcium entry (SOCE) pathway (*63*). To distinguish whether the physical “tethering” function or the role of STIM1 in SOCE causes the decreased ER tension after si-*Stim1* treatment, we introduced Synta66, a highly selective ligand to the ORAI1 extracellular sites, to block SOCE without affecting STIM1 oligomerization and the interaction of STIM1/ORAI1 (*64*). We first evaluated the effect of Synta66 on SOCE signaling in a 6-day culture of tendon cells. Cytosolic Ca^2+^ elevation, recorded by a Ca^2+^ indicator Fluo-4, induced by restoration of extracellular Ca^2+^ to cells pretreated with thapsigargin, a sarco-ER Ca^2+^-ATPase (SERCA)–pump inhibitor, to empty Ca^2+^ stores, was used to evaluate the SOCE ability of cells (Fig. 5, A and B). For tendon cells treated without Synta66, partial *Stim1* knockdown interfered with SOCE activity, compared to the scrambled control–treated cells (Fig. 5, A and B). Compared to partial *Stim1* knockdown without Synta66, Synta66 treatment with scrambled control–treated cells resulted in a more pronounced inhibition of SOCE (Fig. 5, A and B). Synta66 treatment further decreased SOCE activity in si-*Stim1*–treated cells (Fig. 5, A and B). Moreover, partial *Stim1* knockdown cells and scrambled control–treated cells showed a similar degree of inhibition of SOCE after Synta66 treatment (Fig. 5, A and B).

To evaluate the combination effect of Synta66 treatment with partial *Stim1* knockdown on PM-ER tethering, we mapped the PM-ER contact sites on tendon cells with different treatments using a split-GFP–based contact site sensor (SPLICS), SPLICS_L_^ER-PM^. SPLICS_L_^ER-PM^ is able to detect interactions occurring between the PM and the ER within a reciprocal distance of around 40 nm (*56*) (Fig. 5, C and D). By quantification of PM-ER contact sites by confocal microscopy of tendon cells expressing SPLICS_L_^ER-PM^, we found that the number of PM-ER contact sites was lower upon si-*Stim1* treatment (Fig. 5, E and F). Furthermore, treatment with Synta66 did not affect PM-ER contact sites in both si-*Stim1*–treated cells and scrambled control–treated cells (Fig. 5, E and F).

Collectively, the above observations enabled us to develop the strategy of using Synta66 and si-*Stim1* to dissect the roles of physical PM-ER tethering and SOCE in regulating ER tension (Fig. 5G). We measured ER tension by probing with ER Flipper-TR in mouse tendon cells transfected with scrambled siRNA or with *Stim1* siRNA, and treated with or without Synta66, in 2D uniaxial stretching culture with 6% strain. By FLIM imaging, we found a shorter fluorescence lifetime of ER Flipper-TR in si-*Stim1*–treated tendon cells, compared to scrambled control–treated cells, regardless of Synta66 treatment or not (Fig. 5, H and I). Moreover, the fluorescence lifetime of ER Flipper-TR was unaffected by Synta66 treatment in both tendon cells transfected with scrambled siRNA or with *Stim1* siRNA (Fig. 5, H and I). Together with the results from the SOCE assessment and SPLICS_L_^ER-PM^ quantification, we show that detethering of the ER from the PM lowers ER tension under cyclic strain, whereas SOCE does not notably regulate ER tension.

### ER tension dose-dependently associates with ER-mitochondria interaction

**Fig. 6.**
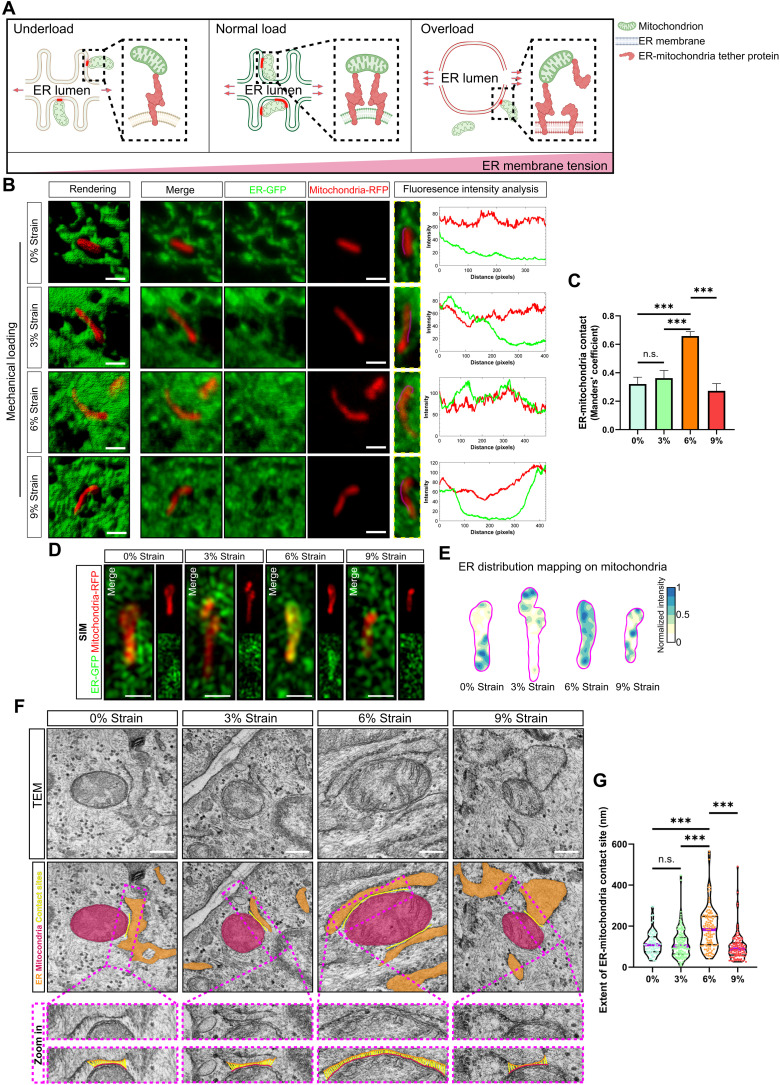
ER tension dose-dependently associates with ER-mitochondria interaction. (**A**) Schematic diagram illustrating our hypothetical model of tension-driven ER-mitochondria interaction. (**B**) Representative 3D confocal images (left) and fluorescence intensity analysis (right) around the mitochondria showing the colocalization between RFP-labeled mitochondria (red) and GFP-labeled ER (green) in 3D tendon constructs receiving 0, 3, 6, or 9% cyclic strain. Scale bars, 1 μm. (**C**) Quantification of the Manders’ coefficient based on data from (B) (*n* = 15 cells from three independent experiments). (**D** and **E**) Representative live-cell SIM images (D) and corresponding color-coded distribution maps of the ER on the mitochondria (magenta) (E) showing the colocalization between RFP-labeled mitochondria and GFP-labeled ER in 3D tendon constructs receiving 0, 3, 6, or 9% cyclic strain. Scale bars, 1 μm. (**F** and **G**) Representative TEM images of 3D tendon constructs receiving 0, 3, 6, or 9% cyclic strain (F). Scale bars, 200 nm. Middle panels show the pseudocolored images highlighting the ER (orange), the mitochondria (red), and ER-mitochondria contact sites (yellow) in different colors. ER-mitochondria contact sites are defined as sites of contact within a reciprocal distance of 30 nm. Bottom panels are the zooms of the purple dashed boxed area in the corresponding middle panel. Yellow lines indicate the ER-mitochondria contacts. The corresponding quantitative analysis to the extent of individual ER-mitochondria contact site (0% strain, *n* = 74; 3% strain, *n* = 127; 6% strain, *n* = 119; 9% strain, *n* = 102; *n*, contact sites from three independent experiments) (G). ***P < 0.001; n.s., not significant by one-way ANOVA (C) or by Kruskal-Wallis test (G). SIM, structured illumination microscopy. Violin plot presents the median and quartiles. Error bars stand for SEM. (A) created with BioRender.com.

We then focused on the adaptive ER tension induced by cyclic strain and sought to identify its correlations with cellular activities. We noted that increased ER tension was correlative with flattened and expanded ER membranes, although only under an excessive loading environment of 9% strain (Fig. 2, E to G). This led us to hypothesize that there is a structure in place that maintains proper ER configurations when tension of its membrane is increased under smaller loads, such as 6% cyclic strain. Previously, it has been shown that moderate tension recruits proteins to the membranes to maintain their curvature (*36*, *65*, *66*), whereas high tension could inhibit protein-protein interactions (*34*). As ER-mitochondria tethering structures consist mainly of interacting proteins that are indispensable for maintaining ER morphology (*67*, *68*), we hypothesized that moderate tension of the ER may drive an increase in the number of ER-mitochondria tether proteins recruited to the complex to maintain ER morphological homeostasis while also increasing the attachment of mitochondria to the ER membranes. On the other hand, excessively high tension may impair the protein-protein interactions between the ER and mitochondria, leading to ER expansion and a decrease in ER membrane curvature (Fig. 6A).

To test this potential link, we characterized the ER-mitochondria tether structure by volume-rendered 3D reconstruction confocal microscopy. Using this approach, we found a partial contact between GFP-labeled ER and red fluorescent protein (RFP)–labeled mitochondria in mouse tendon constructs cultured with underloading via 0 or 3% strain (Fig. 6B). Compared to underloading, normal loading with 6% strain enhanced the interaction between the ER and mitochondria in tendon constructs, with more complete contact between the two organelles (Fig. 6B). However, further increasing of the load to a 9% strain decreased the contact between the ER and mitochondria, leading to uncoupling of the mitochondria from the ER (Fig. 6B). Quantitative analysis revealed the highest Manders’ colocalization coefficient between the ER and mitochondria in tendon constructs receiving 6% strain compared to that in tendon constructs receiving 0, 3, or 9% (Fig. 6C). Live-cell structured illumination microscopy (SIM) and corresponding ER distribution mapping on mitochondria further confirmed the highest colocalization between the ER and mitochondria occurred in tendon constructs receiving 6% strain (Fig. 6, D and E). Furthermore, the ultrastructure of ER-mitochondria contacts as determined by transmission electron microscopy (TEM) confirmed that the longest extent of ER-mitochondria contacts occurred in tendon constructs receiving 6% strain, compared to those receiving 0, 3, or 9% strain (Fig. 6, F and G). In addition, consistent with the results of ER tension, there was no significant difference in the ER-mitochondria colocalization coefficient, or the extent of ER-mitochondria contacts, between tendon constructs receiving 0 and 3% strain (Figs. 2C and 6, B to G), implying that 3% strain was insufficient to increase ER tension compared to 0% strain and thus subsequently did not change the ER-mitochondria interaction nor cell function. Notably, by TEM imaging, we observed that mitochondrial cristae, the unique folded structures of the inner mitochondrial membrane that host the electron transport chain and adenosine triphosphate (ATP) synthase and are closely associated with oxidative phosphorylation function (*69*), were affected by different mechanical strains (fig. S11, A and B). Mitochondrial cristae appeared longest with clear shape in tendon constructs subjected to 6% strain compared to those subjected to 0, 3, or 9% strain.

Together, these data suggested that moderate ER tension is associated with enhanced ER-mitochondria interactions, whereas excessively high ER tension is linked to a disruption of this interaction.

### Mechanical strain affects cellular energetics

**Fig. 7.**
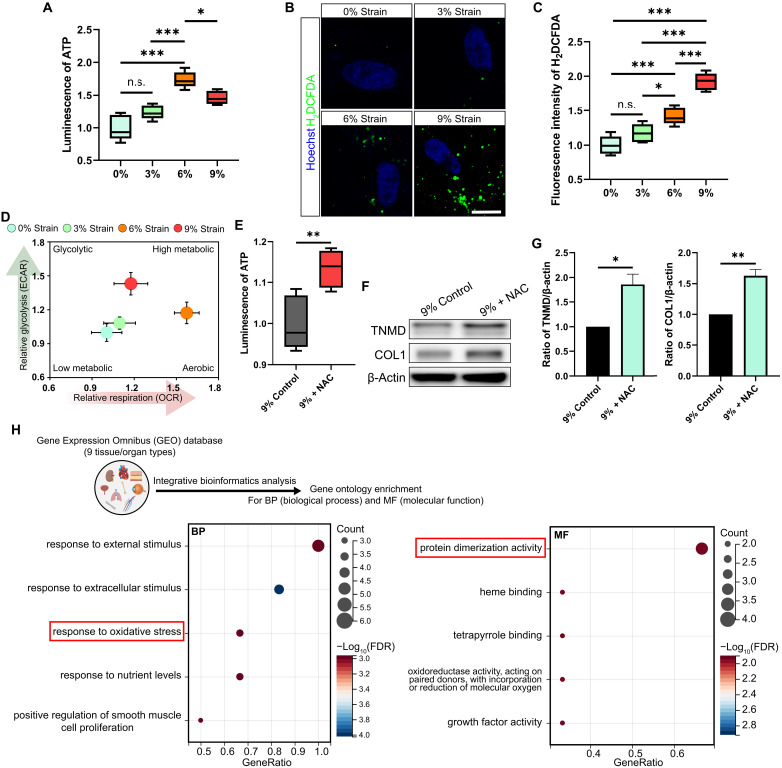
Mechanical strain affects cellular energy metabolism. (**A** to **C**) Luminescence measurement of ATP (A) (*n* = 5 biological replicates from three independent experiments), representative confocal fluorescence images of H_2_DCFDA-labeled ROS (scale bar, 10 μm) (B), and corresponding fluorescence intensity quantification (C) of H_2_DCFDA in 3D tendon constructs receiving 0, 3, 6, or 9% cyclic strain (*n* = 5 biological replicates from three independent experiments). (**D**) Mitochondrial respiration and glycolysis activity of 3D tendon constructs receiving 0, 3, 6, or 9% cyclic strain (*n* = 5 biological replicates from four independent experiments) measured by the OCR and ECAR. (**E** to **G**) Luminescence measurement of ATP (E) (*n* = 5 biological replicates from three independent experiments) and representative immunoblot analysis of TNMD and COL1 expression (F), as well quantitative analysis of the immunoblot data (G) (three biological replicates from three independent experiments), of 3D tendon constructs receiving 9% strain treated with or without NAC. β-Actin expression was measured as the internal control in the immunoblot analysis. (**H**) Bubble plots of integrative bioinformatics analysis showing the top 5 ranked GO terms for biological process (BP; left) and molecular function (MF; right) (by *P* value) for common mechanoresponsive cellular activities. *P < 0.05; **P < 0.01; ***P < 0.001; n.s., not significant by one-way ANOVA (A and C) or by Student’s *t* test (E and G). NAC, *N*-acetylcysteine. Error bars stand for SEM (A, C, E, and G) or SD (D). (H) created with BioRender.com.

The ER-mitochondria tether structure and mitochondrial cristae are essential for cellular energy metabolism (*70*–*72*). Energy metabolism is accompanied by generation of ATP and reactive oxygen species (ROS) (*73*), whereas the levels of ROS indicate the degree of oxidative stress (*74*). Thus, we first measured ATP production of 3D tendon constructs under mechanical strain. We found that the highest ATP production occurred in tendon constructs receiving 6% strain compared to tendon constructs receiving 0, 3, or 9% strain (Fig. 7A). Like the ER tension and ER-mitochondria interaction results, the ATP production in tendon constructs receiving 3% strain was similar to that with 0% strain (Fig. 7A). Next, we measured ROS levels by 2′,7′-dichlorodihydrofluorescein diacetate (H_2_DCFDA) staining. We found that tension induced ROS production in an increasing dose-dependent manner, although with no statistical difference between 0 and 3% strain (Fig. 7, B and C). Although an optimal level of ROS is imperative for redox homeostasis, excessive ROS production is considered pathological (*75*, *76*), which is consistent with our observations that a decreased cell function occurs in tendon constructs receiving 9% strain. Considering that most intracellular ATP is generated through mitochondrial respiration and cytosolic glycolysis (*77*), we extended our investigation to include the measurement of the oxygen consumption rate (OCR) and the extracellular acidification rate (ECAR) in 3D tendon constructs subjected to cyclic strain of 0, 3, 6, or 9%. The data revealed that compared to other conditions, a normal loading condition of 6% strain triggered a metabolic transition in tendon constructs toward an aerobic phenotype (Fig. 7D). This finding, in conjunction with ATP level assessment, suggested that a normal load enhances mitochondrial respiratory activity, thereby boosting ATP production. In contrast, an overload condition of 9% strain was found to inhibit mitochondrial respiration (Fig. 7D). Despite a compensatory increase in glycolytic activity under 9% strain, there was still a net decrease in ATP production (Fig. 7, A and D), indicating a detrimental effect of overload on the energy metabolism of the tendon constructs, especially on mitochondrial respiration. The reduced ATP production aligns with the decline in cellular function observed under 9% strain, suggesting that mitochondrial ATP production may play a critical role in maintaining cellular function. In summary, our data indicate that a normal load boosts cellular energy metabolism, whereas excessive load reduces cellular energetics.

To confirm the relationship between energy metabolism and cell function, we next used *N*-acetylcysteine (NAC), an antioxidant that inhibits ROS (*78*). We found that NAC treatment of tendon constructs receiving a 9% strain resulted in significantly lower levels of ROS as measured by H_2_DCFDA staining, as compared to control-treated strained tendon constructs (fig. S12, A and B). We also examined the effect of NAC treatment on mitochondrial respiration during overload and found that the OCR of tendon constructs significantly increased following NAC treatment under 9% strain (fig. S13). We further treated cells with H_2_O_2_ to elevate ROS levels under 6% strain conditions. The increase in ROS was confirmed by H_2_DCFDA staining (fig. S14A). As expected, mitochondrial respiration, measured by OCR, significantly decreased in cells treated with H_2_O_2_ under 6% strain compared to control-treated strained tendon constructs (fig. S14B), confirming that excessive ROS was detrimental. Because ER stress has been reported to affect mitochondrial function, albeit with contradictory effects observed in different contexts (*79*–*81*), we then sought to determine whether ER stress under overload conditions contributes to mitochondrial dysfunction. To evaluate the relationship between mitochondrial respiration and ER stress, we used tauroursodeoxycholic acid (TUDCA), a chemical chaperone that stabilizes protein conformation and inhibits ER stress (*82*). The inhibition of ER stress under 9% strain following TUDCA treatment was confirmed by the decreased unconventional splicing of *Xbp1* (fig. S15, A and B). OCR measurements showed no significant difference in mitochondrial respiration in 3D tendon constructs subjected to 9% strain with or without TUDCA treatment, suggesting that mitochondrial dysfunction under 9% strain was unaffected by ER stress relief and, therefore, such dysfunction in response to a high strain occurred independently of ER stress (fig. S15C). Furthermore, compared to the control-treated group, ATP production by tendon constructs under a 9% strain was higher upon NAC treatment (Fig. 7E). Moreover, by immunoblotting we found that the expression of the tendon cell functional markers, TNMD and COL1, was significantly greater in tendon constructs under a 9% strain that were treated with NAC treatment compared to controls, indicating an augmentation of metabolic status (Fig. 7, F and G).

To further clarify whether changes in energy metabolism is a common cellular response to mechanical stimulation, we performed an integrative bioinformatics analysis to expression profiles of mechanically stimulated cells from 17 datasets and their matching unstimulated cells across nine tissues/organs from the Gene Expression Omnibus (GEO) database (*83*) (fig. S16A). Intriguingly, meta-analysis by the robust rank aggregation (RRA) method with Gene Ontology (GO) enrichment for biological processes revealed that the “response to oxidative stress” was the top-ranked mechanoresponsive category in which external/extracellular stimulus was the primary treatment (Fig. 7H and fig. S16B). Moreover, we noticed that by meta-analysis with GO enrichment for molecular function, “protein dimerization activity,” which is a crucial process in protein-protein interactions (*84*), was the highest ranked category across different mechanically stimulated tissues/organs (Fig. 7H and fig. S16C). These data suggest that ER tension–related changes in energy metabolism might be a common response to mechanostimulation of mammalian cells.

### Manipulation of the PM-ER tether structure regulates mechanoresponsive energy metabolism

**Fig. 8.**
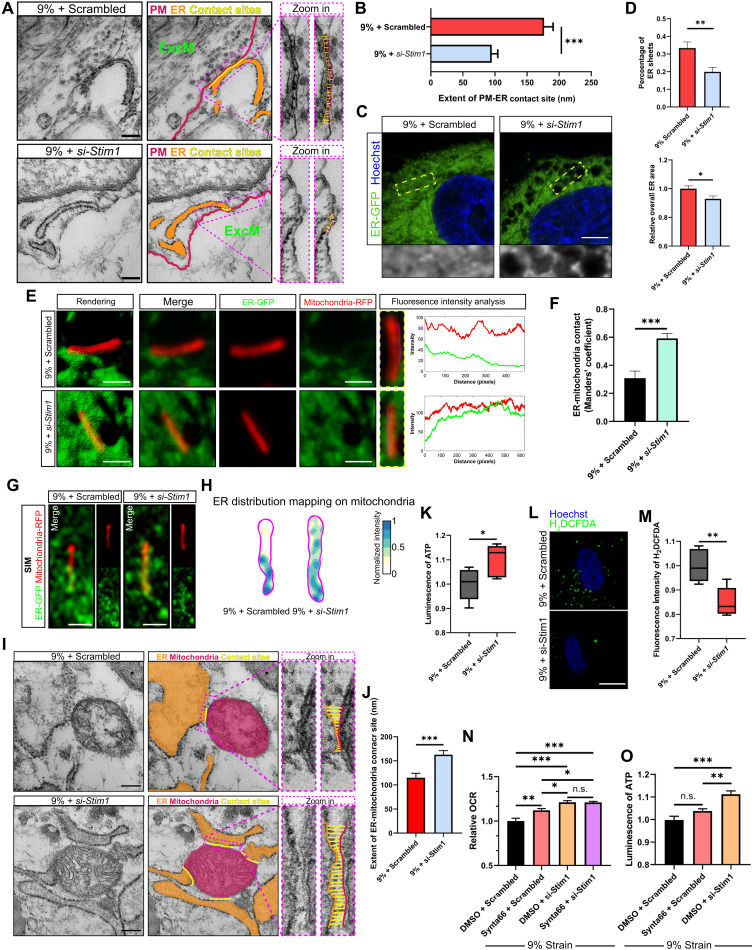
Manipulation of the PM-ER tether structure alters mechanoresponsive energy metabolism and related ER responses. (**A** and **B**) Representative TEM images of 3D tendon constructs transfected with siRNA and cultured under 9% cyclic strain showing PM-ER contacts (A). Middle panels highlighting the PM (red), ER (orange), and contact sites (yellow). PM-ER contact sites, a reciprocal distance of 30 nm. Right panels are from the purple dashed boxed area in the middle panel. ExcM, extracellular matrix. (B) Quantification to the extent of individual PM-ER contact site (*n* = 62 to 73 contact sites). (**C** and **D**) Representative confocal images of GFP-labeled ER (C) and ER morphology analysis (D) in constructs (*n* = 15 cells). (**E** and **F**) Representative 3D confocal images (E) showing colocalization of RFP-labeled mitochondria and GFP-labeled ER in constructs transfected with siRNA under 9% strain and Manders’ coefficient quantification (F) (*n* = 15 cells). (**G** and **H**) Representative SIM imaging (G) and distribution maps of GFP-labeled ER on RFP-labeled mitochondria (H) in constructs receiving 9% strain and transfected with siRNA. (**I** and **J**) Representative TEM images of constructs transfected with siRNA under 9% strain showing the ER-mitochondria contacts (I) and quantification (J) (*n* = 67 to 107 contact sites). (**K** to **M**) ATP luminescence (K), representative confocal fluorescence images of H_2_DCFDA-labeled ROS (L), and H_2_DCFDA quantification (M) (*n* = 5 biological replicates) in constructs receiving 9% strain with siRNA transfection. (**N**) Mitochondrial respiration of tendon constructs transfected with siRNA and treated with DMSO or Synta66 under 9% strain (*n* = 7 biological replicates). (**O**) Luminescence measurement of ATP in constructs receiving 9% strain, transfected with siRNA, and treated with DMSO or Synta66 (*n* = 5 biological replicates). Scale bars, 200 nm (A and I), 10 μm (C and L), or 1 μm (E and G). *n*, replicates per group from at least three independent experiments. *P < 0.05; **P < 0.01; ***P < 0.001; n.s., not significant by Student’s *t* test (B, D, F, K, M, and J) or one-way ANOVA (N and O). Error bars stand for SEM.

Our data show that only optimal ER tension downstream of 6% loading is associated with enhanced energy metabolism, whereas overloading-induced excessive ER tension is linked to harmful effects. We thus then examined whether manipulating the PM-ER tethering system during overloading to reduce ER tension and its related responses could rescue cellular energy metabolism. We disrupted the PM-ER tethering system by partial knocking down *Stim1* expression (fig. S17). By TEM, we confirmed that partial *Stim1* knockdown detethered the ER from the PM in 3D tendon constructs cultured in 9% strain, which led to a reduction of the extent of PM-ER contacts from around 176.6 to 94.9 nm (Fig. 8, A and B). The ultrastructure analysis to PM-ER contacts also revealed heterogeneous ER that contacted with PM (referred to as cortical ER) in the tendon constructs, including wide ER and thin ER (fig. S18, A and B). Compared to wide cortical ER, thin cortical ER features less lumen space (*85*). Quantitative assessment to wide ER and thin ER in tendon constructs transfected with scrambled siRNA or *Stim1*-siRNA and cultured in 9% strain showed that si-*Stim1* treatment only slightly changed the portion of wide ER and thin ER (fig. S18C). In addition, the extent of both wide ER-PM contacts and thin ER-PM contacts was reduced by si-*Stim1* treatment in tendon constructs (fig. S18D). Also, by GFP labeling of the ER and by confocal microscopy, we found that, compared to the transfection with scrambled siRNA, si-*Stim1* treatment alleviated the expansion of ER content and changes in ER morphology that occurs during overloading (Fig. 8, C and D). Furthermore, on the basis of our geometric measurements, we used a computer-aided cell modeling approach to hypothesize the role of PM-ER contacts in maintaining ER mechanical homeostasis under stretching, yielding results consistent with our experimental observations (text S1 and fig. S19).

Next, we explored the effects of PM-ER tether structure on ER-mitochondria interaction. Using 3D reconstruction of confocal imaging, we found that partial knockdown of *Stim1* could rejuxtapose GFP-labeled ER to RFP-labeled mitochondria after overloading with 9% strain (Fig. 8E). The Manders’ coefficient indicated a higher colocalization between the ER and mitochondria in tendon constructs cultured under 9% strain and transfected with *Stim1* siRNA compared to scrambled siRNA (Fig. 8F). By live-cell SIM, we also showed that partial *Stim1* knockdown in tendon constructs cultured under 9% strain could recolocalize the ER to mitochondria, as indicated by an increasing ER distribution mapping on mitochondria (Fig. 8, G and H). The ultrastructure of ER-mitochondria contacts revealed by TEM further confirmed that, compared to the transfection with scrambled siRNA, si-*Stim1* treatment could rejuxtapose ER to mitochondria after overloading with 9% strain, by increasing the extent of ER-mitochondria contacts (Fig. 8, I and J). Furthermore, by TEM analysis followed by mitochondrial cristae length measurements, we found that *Stim1*-siRNA treatment increased mitochondrial cristae length under 9% strain, suggesting that partial *Stim1* knockdown preserved mitochondrial cristae integrity and protected them from damage under overload conditions (fig. S20).

Consistent with the ER-mitochondria interaction results, we also found that partial knockdown of *Stim1* could further reboost ATP production and decrease ROS accumulation in tendon constructs cultured overloaded with 9% strain, indicating that partial knockdown of *Stim1* could rescue the energy metabolism deficits induced in tendon constructs by overloading (Fig. 8, K to M). Consistent with these results, we found by immunoblotting of tendon cell functional markers that partial knockdown of *Stim1* with two different doses of siRNA could induce a significant increase in expression of TNMD in tendon constructs cultured with 9% strain compared to scrambled control–treated cells under the same strain, whereas the higher dose of si-*Stim1* also significantly increased the expression of COL1 (fig. S21, A and B).

We then evaluated whether si-*Stim1* treatment reboosted cellular energetics by its physical “tethering” function or the role of STIM1 in SOCE cultured with overload. As we found that mitochondrial respiratory activity was the main factor affecting ATP production regulating by cyclic strain (Fig. 7D), we measured mitochondrial respiration of 3D tendon constructs transfected with scrambled siRNA or *Stim1*-siRNA and further treated with or without Synta66, after 9% cyclic strain. Partial knockdown of *Stim1* significantly raised the OCR in tendon constructs cultured with 9% strain, confirming the essential role of STIM1 in regulating cellular energetics by affecting mitochondrial respiration (Fig. 8N). The OCR also showed that, compared to 3D tendon constructs transfected with scrambled siRNA and treated without Synta66, Synta66 treatment of 3D tendon constructs transfected with scrambled siRNA exhibited a higher mitochondrial respiration, indicating that overload affected the OCR via the SOCE to some extent (Fig. 8N). However, compared to tendon constructs transfected with scrambled siRNA and treated with Synta66, partial knockdown of *Stim1* induced a higher OCR in tendon constructs (Fig. 8N). As Synta66 led to a more pronounced inhibition of SOCE than partial *Stim1* knockdown (Fig. 5, A and B), these data implied that both physical PM-ER tethering and SOCE contributed to the regulation of mitochondrial respiration but that physical PM-ER tethering had a more dominant effect than SOCE. Moreover, further repression of SOCE activity in partially *Stim1*-knockdown tendon constructs followed by Synta66 treatment demonstrated no significant difference in the OCR, compared to si-*Stim1*–treated tendon constructs without Synta66 treatment (Fig. 8N). As we revealed the regulatory role of ROS in mitochondrial respiration during overload, we then evaluated ROS levels in 3D tendon constructs transfected with scrambled siRNA or *Stim1*-siRNA and treated with or without Synta66 under 9% cyclic strain (fig. S22). ROS levels decreased following Synta66 treatment, confirming that SOCE also contributed to ROS regulation. However, compared to tendon constructs transfected with scrambled siRNA and treated with Synta66, partial *Stim1* knockdown resulted in lower ROS levels, suggesting that PM-ER physical tethering played a more dominant role than SOCE in ROS regulation. In addition, inhibition of SOCE activity by Synta66 in tendon constructs transfected with si-*Stim1* showed no significant difference in ROS levels compared to si-*Stim1*–transfected constructs without Synta66 treatment. We further investigated the role of physical PM-ER tethering and SOCE in ATP production. ATP measurements revealed that under 9% strain, partial *Stim1* knockdown significantly increased ATP production in tendon constructs to levels higher than those observed in control-treated tendon constructs and those treated with Synta66 (Fig. 8O). Although there was a trend of increased ATP production with Synta66 treatment, the difference was not statistically significant compared to untreated tendon constructs under 9% strain (Fig. 8O). Furthermore, Synta66 treatment did not significantly alter ATP production in si-*Stim1*–treated tendon constructs cultured under 9% strain (fig. S23). Together with the mitochondrial respiration and ROS results, these findings suggest that, although SOCE contributes to cellular energetics, the role of PM-ER physical tethering in regulating ROS, mitochondrial respiration, and subsequent ATP production cannot be overlooked.

Together, these data indicate that reducing the PM-ER tether structure in an overload environment reboosts cellular energetics and ameliorates cell dysfunction.

## DISCUSSION

**Fig. 9.**
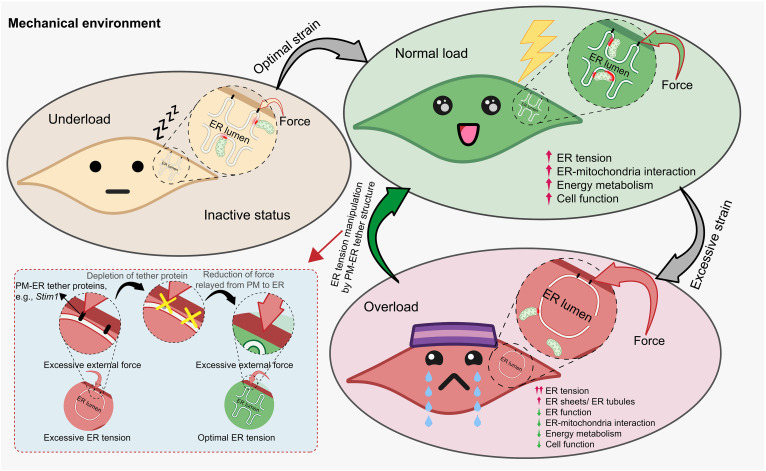
Schematic diagram illustrating a tension-related PM-ER mechanotransduction mechanism that dose-dependently regulates cellular metabolism. Mechanical strain can be transferred from the PM to the ER on MCSs via PM-ER tether structures composed of tether proteins that localize to contact sites between the two membranes, most notably STIM1. The repeatedly mechanical forces then increase ER tension as a mechanoadaptation mechanism in a dose-dependent manner. Furthermore, the mechanical strain leads to the regulation of ER-mitochondria interactions that ultimately associates with the degree of mitochondrial function and cellular function. In particular, optimal mechanical loading leads to strengthening of the ER-mitochondria interactions and a boosting of energy metabolism, as well as greater cell function. On the other hand, mechanical overloading leads to a pathological expansion of ER content, an uncoupling of ER-mitochondria contacts, increased ER stress, and an impairment in cellular energy metabolism, as well as reduced cell function. Furthermore, detethering the ER from the PM alleviated mechanical stress in the ER under stretching. Therefore, we can manipulate this system by genetic suppression of *Stim1* expression to improve outcomes in response to overloading. Figure created with BioRender.com.

In the present study, we focus on the aftereffects of mechanical loading on cells. We identified a PM-ER mechanotransduction pathway that regulates cellular energetics (Fig. 9). Our model suggests that external mechanical stress can be relayed from the PM to the ER via MCS-localized tether proteins, most notably STIM1, which then alters ER tension. This process of mechanoadaptation is dose dependent, although with a required minimal force and a duration threshold. Furthermore, the adaptive changes of ER tension are associated with ER-mitochondria interactions and the degree of mitochondrial and cellular functions. Optimal mechanical loading leads to strengthening of the ER-mitochondria interactions and a boosting of energy metabolism, as well as greater cell function. On the other hand, mechanical overloading leads to a pathological expansion of ER content, an uncoupling of ER-mitochondria contacts, increased ER stress, and an impairment in cellular energy metabolism, as well as reduced cell function. Moreover, manipulation of the PM-ER mechanotransmission pathway by genetic suppression of *Stim1* expression alters outcomes in response to overloading.

Using a highly selective SOCE inhibitor, Synta66, that does not affect STIM1-ORAI1 coupling, we elucidated the role of physical tethering in STIM1 function. Previous studies have shown that STIM1 is responsible for cell migration, but Synta66 treatment did not affect this cell behavior (*64*, *86*). Together with our results, these findings indicate that, although each tether protein may have a unique function, their impact on the physical bridging of two membranes in various sites perhaps deserves more attention. Moreover, STIM1-mediated PM-ER tethering has been commonly investigated under a SOCE-activated situation. The present study demonstrated a STIM1-mediated PM-ER tethering in a SOCE activation–independent manner, which is consistent with a previous report (*56*). A study revealed that ~50% of STIM1 puncta at MCSs in cells with depleted stores already exist before the depletion occurs (*62*). The SOCE-independent mediation of PM-ER contacts by STIM1 perhaps occurs via these STIM1 puncta, by a yet to be elucidated mechanism. Furthermore, the ultrastructure analysis of PM-ER contacts in the present study showed heterogeneous cortical ER in the tendon constructs, including both wide ER and thin ER. Thin ER expands in cells overexpressing STIM1, leading to a conclusion that STIM1 is related to thin ER (*87*). However, it remains to be demonstrated whether endogenous STIM1 is concentrated in the thin ER, which needs high-quality antibodies (*85*, *87*). Our work shows that the extent of both wide ER-PM contacts and thin ER-PM contacts was affected by *Stim1* knockdown in tendon constructs, highlighting an essential impact of STIM1 on general PM-ER contacts.

The membrane tension we reported reflects adaptive changes resulting from long-term cyclic stretching, retained by the cells after the cessation of mechanical stimuli. Developing these adaptive changes typically involves rapid mechanosensing and mechanotransduction events, occurring within minutes to hours (*88*). However, the imprinting of adaptive changes often requires a longer duration, ranging from days to weeks, which aligns with our observations that a duration threshold was needed to induce these adaptive tension changes. The underlying mechanism of imprinting adaptive changes remains unclear.

Membrane tension could be influenced by changes in ER properties, including the membrane area-to-volume ratio, lipid composition and distribution, transmembrane protein content, or interactions with contractile elements and other subcellular structures (*8*, *89*, *90*). Irreversible changes of these factors, caused by physical alterations or cellular reprogramming downstream of PM-ER tethering mechanotransduction pathway, may offer plausible explanations for the observed adaptive membrane tension. A deeper understanding of adaptive membrane tension would be valuable for developing targeted approaches to directly manipulate it.

PM tension can be influenced by stretching as well as other mechanical forces, such as shear stress– and adhesion-induced forces (*91*, *92*). The latter are mostly influenced by the micropattern and stiffness of the ECM. It is likely that these mechanical forces also affect ER tension. We noticed variations in baseline ER tension under static conditions across different culture systems, such as glass-bottom dishes compared to silicon substrates in the bioreactor. This suggests that the culture substrate may influence ER tension, a hypothesis that warrants further investigation. In addition, investigating the role of the PM-ER tethering pathway in regulating cellular activities under different types of mechanical loading conditions, particularly its effects on ER behavior, is in need of further study.

Understanding of mechanotransduction for tensile strain to cells is essential for developing treatments in many clinical cases. Excessive cyclic strain to vascular cells induced by hypertension leads to atherosclerosis (*93*); to tendon cells induced by overuse especially in athletes causes tendinopathy (*94*); to podocytes induced by glomerular capillary hypertension results in glomerulosclerosis (*95*); to retinal Muller cells induced by pathological myopia leads to blindness (*96*); to bladder smooth muscle cells induced by chronic overdistension causes bladder hypertrophy and decompensation (*97*); to dermal fibroblasts following cutaneous trauma leads to hypertrophic scar (*98*). Some clinical operations may cause tension-stress and hurt resident cells. For example, positive end-expiratory pressure ventilator–induced tensile stress may impair epithelial cells through alveolar hyperinflation (*99*). Mechanical stimulation is also the key factor for governing stem cell differentiation in tissue engineering to produce valid alternative for tissue replacement in treatments (*100*, *101*). Besides, metabolic responses to physical stimulation and stress are extremely complex, involving interacting variables (*102*). The present study indicates that the mechanoadaptation mechanism on ER tension was related to ER-mitochondria contact sites and further tied to the alteration of cellular energetics and cellular function. Mechanical properties of subcellular structures have been linked to cellular function (*103*). The present study further emphasizes this relationship and unmasks this link between cyclic strain and metabolic response of cells, which lastly provides a potential strategy to regulate mechanotransduction by the PM-ER mechanotransmission pathway.

Our work identifies a previously unknown mechanotransduction pathway, but one that does not conflict with established classical pathways. Although our data suggest that mechanotransduction can be modulated by PM-ER contact sites without markedly altering the actin cytoskeleton, it is important to emphasize that the experiments conducted in this study to explore the PM-ER mechanotransduction were based on an intact cytoskeletal system. The actin cytoskeleton offers a stable supporting structure for force transmission and provides a scaffold where signaling molecules dock and interact (*104*). Alterations in the actin cytoskeletal structure have been shown to influence YAP/TAZ localization and cellular energetics (*105*, *106*). Our data also indicate that the cytoskeleton is critical for maintaining tension in the endomembrane system, as evidenced by the significant reduction in both ER and PM tension following cytochalasin D treatment. This reduction may result directly from cytoskeletal disruption or indirectly through effects on other mechanosensing compartments, such as the nucleus. Therefore, the mechanotransduction pathways through PM-ER contact sites and the actin cytoskeleton cannot be seen as disassociated parallel mechanisms. The facilitation of the cytoskeleton in the PM-ER mechanotransduction pathway can be conceptualized by hypothesizing that the cytoskeleton forms a scaffold supporting the entire cell and providing spatial organization for the endomembrane system. The PM-ER tethering structure anchors the ER to the cell border, enabling it to sense mechanical signals transmitted from the PM. Therefore, the PM-ER tethering pathway is a necessary, although not sufficient, condition for the regulation of membrane tension.

Mechanosensitive ion channels are another established mechanotransduction pathway. It has been reported that mechanical loading activates mechanosensitive ion channels, such as Piezo1 and TRPV4, facilitating calcium influx from the extracellular space into the cytoplasm (*107*, *108*). Our work highlights the role of PM-ER physical tethering in regulating cellular energetics. However, we also found that SOCE notably contributes to cellular energetics, particularly in regulating ROS production and mitochondrial respiration. The role of SOCE in dynamic mechanical loading environments may involve modulating intracellular calcium levels. Mechanical overload is often associated with excessive intracellular calcium, which is linked to mitochondrial dysfunction and oxidative stress (*109*, *110*). As an alternative mechanism for increasing intracellular calcium levels, inhibition of SOCE could rescue mitochondrial function and mitigate oxidative stress, potentially by alleviating calcium overload. This provides further evidence that these mechanotransduction pathways are essential and function collaboratively.

Other established mechanosensitive pathways may also collaborate with the PM-ER mechanotransduction pathway. The nucleus, another key mechanosensitive organelle, can sense mechanical forces primarily through pathways that transmit signals from focal adhesions to the PM, then to the cytoskeleton, and ultimately to the linker of nucleoskeleton and cytoskeleton (LINC) complex (*111*). Nuclear deformation can regulate cellular behavior by affecting nuclear pores and modulating the transport of proteins through these pores (*112*, *113*). Considering that the outer nuclear membrane is continuous with the ER membrane, it is conceivable that ER tension may influence nuclear membrane tension and, subsequently, the highly curved pore membrane connecting the inner and outer nuclear membranes (*114*). Furthermore, it is possible that mechanical forces propagate from the PM to the ER and then to the nuclear membrane, bypassing cytoskeletal transmission, which remains a largely unexplored area of research. In this study, we also revealed a relationship between PM-ER tethering and ROS. Because ROS have been reported to directly affect genomic stability (*115*), dissecting the mechanisms of force propagation to the nucleus, whether directly through the ER, via the cytoskeleton, or through indirect factors such as ROS, will be critical for advancing our understanding of intracellular mechanotransduction. Conversely, nuclear deformation may also propagate mechanical forces to the ER through the nuclear envelope, potentially affecting ER dynamics, a topic that merits further exploration.

This study sheds light on PM-ER physical tethering in mechanotransduction while acknowledging its limitations. In this study, we primarily focused on investigating the aftereffects of mechanical loading on cells as these parameters may be more directly linked to cellular function. Although we propose that mechanical strain is transmitted directly through PM-ER contact sites, supported by substantial indicative evidence and rationalized by our computational models, we acknowledge the need for further studies to explore acute strain transmission in cells, particularly with or without *Stim1* knockdown. Such investigations will require instantaneous measurements of cells during stretching, without time delays.

Although we found that adaptive ER tension was linked to ER-mitochondria contacts and metabolism, and speculated on the role of ER tension in regulating these processes, the precise regulatory role of adaptive ER tension in modulating ER-mitochondria contacts and metabolism remains unclear. Addressing this question would require a more targeted approach to directly manipulate adaptive ER tension.

Besides the ER-mitochondria interaction, there might be some other downstream events triggered by mechanical stimulation to the ER and further contribute to alterations in cellular activities, which require further investigation. For example, a recent study using a light-inducible mechanostimulator of the ER demonstrated that ER mechanostimulation inhibited ER-to-Golgi trafficking, which may be related to the expanded ER membrane observed in the current study (*116*). Piezo-1, the mechanosensitive ion channels on the PM, has also been found to localize to the ER (*117*). Similarly, Pannexin-1, another mechanosensitive channel, has been identified in the ER, where it may induce calcium efflux into the cytosol (*118*). These signals could potentially affect cellular energetics by regulating calcium homeostasis.

In the present study, we proposed and validated a morphological and functional homeostatic process involving the ER and the mitochondria mediated by PM-ER tethering. Together, the PM-ER tethering mechanotransduction and tension-related cellular activities described here have essential implications for our understanding of the mechanotransduction pathway while providing previously unknown insights into the regulation of cell metabolism during physiological and pathological mechanical variations that may aid our understanding of mechanotransduction-related disorders.

## MATERIALS AND METHODS

### Experimental design

#### Isolation and culture of mouse tendon cells

Animals were housed and maintained in accordance with the Institutional Animal Care and Use Committee guidelines of the University of Western Australia (approval number RA/3/100/1731). The middle third of the patellar tendons and Achilles tendons were excised by careful dissection from 6- to 8-week-old C57BL/6 mice of both sexes after euthanizing by cervical dislocation and subsequently rinsed in phosphate-buffered saline (PBS) containing penicillin (100 U/ml) and streptomycin (100 μg/ml) (Gibco). Tendon tissues were digested by type II collagenase (3 mg/ml) in α-minimal essential medium (MEM Alpha; Gibco) for 3 hours and then passed through a 70-μm cell strainer to yield single-cell suspensions. After centrifugation, cells were then cultured in complete medium containing MEM Alpha, 10% fetal bovine serum (FBS; Gibco), penicillin (100 U/ml), and streptomycin (100 μg/ml). Incubation conditions were 37°C in a humidified atmosphere with 5% CO_2_. Passage 3 was used for all experiments described below.

#### 2D and 3D mechanical stimulation of tendon cells

A well-established bioreactor system was constructed to provide cyclic strain to mouse tendon cells, as previously published (*39*, *119*). Briefly, for 2D uniaxial stretching experiments, tendon cells were cultured on collagen type I–coated silicon substrate (Flexcell International) to ~60% confluence in complete medium. Tendon cells were then subjected to a custom-made bioreactor (*37*) with 0 or 6% uniaxial cyclic strain at 0.25 Hz, 8 hours/day, for 6 days, unless stated otherwise. For 3D uniaxial stretching experiments, the tendon cells were first cultured to full confluence as a monolayer and then stimulated by connective tissue growth factor (25 ng/ml; 120-19, PeproTech) and ascorbic acid (4.4 μg/ml; A0278, Sigma-Aldrich) for 6 days to deposit the ECM and then to generate cell sheets. Cell sheets were then collected after detaching them with 0.25% trypsin and were subsequently attached to custom-made tissue hooks to form 3D constructs. 3D tendon constructs were then subjected to the bioreactor system in complete culture medium containing MEM Alpha, 10% FBS, penicillin (100 U/ml), and streptomycin (100 μg/ml), with 0, 3, 6, or 9% uniaxial cyclic strain at 0.25 Hz, 8 hours/day, for 6 days. The middle areas of samples were used for downstream analysis.

#### Cell transfections and chemical treatments

Tendon cells were transfected with *Stim1* siRNA (s13563, Thermo Fisher Scientific) and negative control scrambled siRNA (4390843, Thermo Fisher Scientific) at 10 nM unless stated otherwise, using Lipofectamine RNAiMAX Transfection Reagent (Invitrogen) for a 6-hour incubation according to the manufacturer’s protocol. GFP-MAPPER was a gift from J. Liou (Addgene plasmid no. 117721; http://n2t.net/addgene:117721; RRID:Addgene_117721). SPLICS_L_^ER-PM^ was a gift from M. Brini and T. Calì (Addgene plasmid no. 164111; http://n2t.net/addgene:164111; RRID:Addgene_164111). Tendon cells were transfected with GFP-MAPPER using Lipofectamine 3000 Transfection Reagents (Invitrogen) and incubated for 24 hours per the manufacturer’s protocol. Further transfection with SPLICS_L_^ER-PM^ to siRNA-transfected tendon cells was performed using Lipofectamine 3000 Transfection Reagents (Invitrogen) and incubated for 24 hours per the manufacturer’s protocol on the day after siRNA transfection.

In stretching experiments, transfections were conducted on the day before assembling samples in the bioreactor. To label ER and mitochondria in 3D tendon constructs, cell sheets were incubated with baculovirus-packaged DNA constructs, mitochondria-RFP (C10601, CellLight, Thermo Fisher Scientific) BacMam 2.0, and ER-GFP BacMam 2.0 (C10590, CellLight, Thermo Fisher Scientific) for 16 hours at 37°C, and then assembled on custom-made tissue hooks to form 3D constructs for mechanical stimulation. All cells were tested for mycoplasma contamination using a MycoTOOL kit (Roche) routinely.

For chemical treatments, tendon cells were incubated with 20 μM cytochalasin D (C2618, Sigma-Aldrich) for 20 min, followed by washing with PBS, or with 0.5 μM cytochalasin D for a 6-day culture. Tendon cells or 3D tendon constructs were incubated in complete culture medium supplied with 10 μM Synta66 for 6 days, with or without mechanical stretching. 3D tendon constructs were incubated in complete culture medium containing 2.5 mM NAC or 750 μM TUDCA (580549, Millipore) during mechanical stretch experiments. To elevate ROS levels, tendon constructs were incubated in complete culture medium supplied with 25 μM H_2_O_2_ (Sigma-Aldrich) for 6 days, with the medium changed daily.

#### FLIM acquisition and analysis

In cyclic stretching experiments, cells on silicon membrane or 3D tendon constructs were moved from the bioreactor system to 35-mm glass-bottom petri dishes (P35G-0.17-14-C, MatTek). For Flipper-TR and ER Flipper-TR costaining, mouse tendon cells were directly cultured in 35-mm glass-bottom petri dishes. Samples were then incubated with 1 μM Flipper-TR (SC020, Spirochrome), 1 μM ER Flipper-TR (SC021, Spirochrome), 1 μM Mito Flipper-TR (SC023, Spirochrome), or 1 μM Lyso Flipper-TR (SC022, Spirochrome) for 15 min and washed with live-cell imaging solution (A14291DJ, Invitrogen) before imaging. Imaging was performed using Nikon A1R with a PicoQuant FCS/FLIM module in Ti-E (inverted). Picosecond pulsed 485-nm laser was used for excitation. An Oko Labs stage top incubation system with Perfect Focus System (PFS) was equipped for live-cell imaging at 37°C, with 5% CO_2_. Cells in the middle area of the silicon membrane in 2D stretching experiments or in the middle area of 3D tendon constructs in 3D stretching experiments were scanned. SymPhoTime 64 software (PicoQuant) was used to analyze FLIM data. The fluorescence decay data (from full images or regions of interest) were fitted with a double-exponential. Two decay times were extracted as τ1 and τ2. The longest lifetime with the higher fit amplitude τ1 was used to report membrane tension.

#### Immunoblot analysis

The middle parts of 3D tendon constructs were obtained and first ground to fine powders in a mortar chilled with liquid nitrogen. Proteins from 3D tendon constructs and monolayer tendon cells were extracted by incubating in radioimmunoprecipitation assay lysis buffer [50 mM tris-HCl (pH 7.5), 150 mM NaCl, 1% Nonidet P-40, 0.1% SDS, and 0.5% sodium deoxycholate] supplemented with phenylmethylsulfonyl fluoride (100 μg/ml), 1 mM sodium orthovanadate, DNase (500 μg/ml), and a protease inhibitor cocktail (Roche), for 30 min at 4°C. Supernatants of lysates were collected after centrifugation, diluted with 4× SDS sampling buffer and boiled for 5 min. Proteins were separated by SDS–polyacrylamide gel electrophoresis and transferred to nitrocellulose membranes (Millipore). Membranes were blocked with 5% skim milk for 1 hour at room temperature and then probed by primary antibodies against STIM1 (1:250; s6197, Sigma-Aldrich), β-actin JLA20 (1:5000; Developmental Studies Hybridoma Bank), TNMD (1:100; ab203676, Abcam), COL1 (1:1000; ab260043, Abcam), and the corresponding horseradish peroxidase–conjugated secondary antibodies (Sigma-Aldrich). Immunoreactivity was detected using the Western Lightning Ultra Detection Kit (PerkinElmer) and visualized by the ChemiDoc MP Imaging System (Bio-Rad Laboratories Inc., USA). Relative protein levels were analyzed by ImageJ (National Institutes of Health).

#### RT-PCR and quantitative real-time polymerase chain reaction

RNAs from tendon cells and the middle parts of 3D tendon constructs were extracted using the TRIzol reagent (15596-026, Invitrogen) and PureLink RNA Mini Kit (Invitrogen, Thermo Fisher Scientific, USA). 3D tendon constructs were first ground to fine powders in a mortar chilled with liquid nitrogen. cDNA was obtained using MoMLV (Moloney murine leukemia virus) reverse transcriptase (Promega) after a two-step reaction. A NanoDrop 2000 spectrophotometer (Thermo Fisher Scientific) was used to quantify the RNA concentration. For RT-PCR, PCR products were electrophoresed in 5% agarose gel and then visualized by the ChemiDoc MP Imaging System (Bio-Rad Laboratories Inc., USA) after dyeing with SYBR Safe DNA gel stain (S33102, Invitrogen). For quantitative real-time polymerase chain reaction, PCR products were analyzed with iTaq Universal SYBR Green Supermix (Bio-Rad) in a CFX Connect Real-Time PCR Detection System (Bio-Rad), which was performed in triplicate for each sample. Each experiment was repeated by three individual samples from each group. Primers for the selected genes are listed in table S1.

#### Monolayer cell and 3D tendon construct confocal imaging and processing

To observe actin, tendon cells were seeded on 13-mm microscope coverslips (ProSciTech) placed in 24-well plates at a density of 10^4^ cells per well. Cells were stained by CFSE (1 μM, 15 min) after treatment, washed with PBS, fixed in 4% paraformaldehyde (PFA) for 15 min, permeabilized with 0.1% Triton X-100 in PBS for 5 min, and subsequently stained with rhodamine phalloidin (1:500; R415, Thermo Fisher Scientific) to visualize F-actin and Hoechst 33342 (1:5000; 62249, Thermo Fisher Scientific) to visualize the cell nucleus for 15 min at room temperature. Cells were then washed three times with PBS and mounted onto microscope slides (ProSciTech) by ProLong Diamond Antifade Mountant (P36970, Invitrogen). For live-cell imaging of PM-ER junctions, mouse tendon cells expressing GFP-MAPPER were seeded on 35-mm glass-bottom petri dishes (P35G-0.17-14-C, MatTek) and observed in live-cell imaging solution (A14291DJ, Invitrogen).

For SPLICS_L_^ER-PM^ imaging, tendon cells transfected with SPLICS_L_^ER-PM^ were fixed in 4% PFA for 15 min and subsequently stained with Hoechst 33342 (1:5000; 62249, Thermo Fisher Scientific) to visualize the cell nucleus for 15 min at room temperature. Cells were then washed three times with PBS and mounted onto microscope slides (ProSciTech) using ProLong Diamond Antifade Mountant (P36970, Invitrogen).

For ER morphology and ER-mitochondria contact imaging, 3D tendon constructs transfected with mitochondria-RFP BacMam 2.0, and ER-GFP BacMam 2.0 were fixed in 4% PFA for 1 hour at room temperature, snap-frozen in liquid nitrogen, and embedded in optimal cutting temperature compound (OCT compound) (Tissue-Tek). 3D tendon constructs were sectioned on a cryostat microtome (Leica) into 5-μm slices. The slides with samples were washed with PBS to remove OCT, stained with Hoechst 33342 as described above, and mounted onto microscope slides using SlowFade Glass Soft-set Antifade Mountant (S36917, Invitrogen).

For ThT staining, the 3D tendon constructs were moved from the bioreactor system to 35-mm glass-bottom petri dishes (P35G-0.17-14-C, MatTek) and subsequently stained with 10 μM ThT (ab120751, Abcam) for 30 min. Then, the live 3D tendon constructs were washed three times and observed in live-cell imaging solution (A14291DJ, Invitrogen).

Confocal microscopy imaging was performed on a Nikon A1Si confocal microscope or a Nikon A1R confocal microscope with a 60x oil immersion objective, or a Nikon PlanApo 100x oil immersion objective, or a 60x water immersion objective, equipped with PFS. Live-cell imaging was performed with a Tokai Hit incubation chamber or an Oko Labs stage top incubation system at 37°C, with 5% CO_2_. *Z*-stacks of 0.1- to 0.15-μm interstack interval were acquired for 3D imaging, except for SPLICS_L_^ER-PM^ imaging, in which *Z*-stacks were acquired for the entire depth of the cell by sampling at 0.3 μm in the *z* plane. Images were subjected to denoising, deconvolution, and rendering with NIS-Elements Advanced Research (Nikon) and Imaris (Bitplane).

#### 3D tendon construct SIM imaging and processing

For live-cell ER-mitochondria contact SIM imaging, 3D tendon constructs transfected with mitochondria-RFP BacMam 2.0 and ER-GFP BacMam 2.0 were moved from the bioreactor system to 35-mm glass-bottom petri dishes (P35G-0.17-14-C, MatTek) and observed in live-cell imaging solution (A14291DJ, Invitrogen). SIM imaging was performed on an inverted Nikon Ti2 microscope, equipped with the Piezo stage and PFS III. Imaging was acquired with a Nikon HP Apo TIRF 100x oil immersion objective and a Hamamatsu OCRA FLASH 4 sCMOS camera, in 3D stack SIM mode with *Z*-stacks of 0.125 μm interstack interval. Tokai Hit stage top incubator was used for live-cell imaging at 37°C with 5% CO_2_. SIM data processing and reconstruction were performed in Fourier space and displayed in maximum intensity projection by NIS Elements software (Nikon) and ImageJ (National Institutes of Health).

#### TEM imaging and processing

3D tendon constructs were moved from the bioreactor and transversely cut into 1-mm-thick sections. The middle portions were used for further processing. After fixation with 2.5% glutaraldehyde and 2% PFA in 0.1 M sodium cacodylate buffer, fixed tendon constructs were washed in PBS and immersed in 1% osmium tetroxide in a Biowave Microwave Tissue Processor set at 100 W for 6 min. Subsequently, tendon constructs were dehydrated using ascending ethanol series and dry acetone. Last, the samples were infiltrated and embedded in resin. Ultrathin longitudinal sections of 70 nm were cut using an ultramicrotome (Leica, UC6). The sections were stained with 1% aqueous uranyl acetate for 5 min, rinsed in 50% methanol and distilled water, and then stained with Sato’s modified lead citrate for 5 min. Images were taken on an F200 transmission electron microscope (JEOL) with camera OneView 4k (Gatan) and DigitalMicrograph software (Gatan) at 200 kV.

#### Image analysis

ER morphological analysis was modified from a previously published protocol (*120*). Briefly, the images were first converted to the bit depth of 8 with an intensity from 0 to 255 and then separated by Renyi entropy threshold setting to binary thresholded images. Three identical 56-pixel-wide line segments were drawn from the nuclear envelope to the cell periphery. The percentage of ER sheets in each line segment was calculated by dividing the number of ER sheets pixels by the number of total ER pixels. For overall ER area calculation, the cytoplasm was scrutinized. The overall ER area was then normalized by the area of cytoplasm examined. For F-actin orientation color coding, images were rotated to make the long axis of the cell vertical. Then, 0° was defined as vertical, and 90° was defined as horizontal. For spatial distribution mapping of ER on mitochondria, reconstructed SIM images were segmented using the threshold of mitochondria-RFP signals. Normalized intensity of ER-GFP was color coded and used to map onto the mitochondria. For quantification of SPLICS_L_^ER-PM^, a published protocol was followed (*121*). Briefly, a complete *Z*-stack was processed using plugins (available at https://github.com/titocali1/Quantification-Plugins). A 3D reconstruction of the resulting image was then obtained, thresholded, and counted automatically. All images were analyzed by ImageJ (National Institutes of Health).

#### SOCE assessment

Tendon cells were cultured in a 96-well plate and, where indicated, transfected with siRNA and further treated with Synta66 as described above. Tendon cells were loaded with Fluo-4 NW (F36206, Invitrogen) for 45 min at 37°C and then subjected to the “calcium readdition” protocol by switching between buffer solutions with or without extracellular calcium, combined with thapsigargin treatment as previously established (*122*). Briefly, cells were perfused with 0 mM Ca^2+^ Hanks’ balanced salt solution (HBSS) for 5 min to remove extracellular calcium and then treated with 2 μM thapsigargin for 30 min to deplete the ER calcium store, after which 2 mM Ca^2+^ HBSS were rapidly reintroduced. Ca^2+^ imaging was acquired every 5 s. Assessment was performed by a cell imaging multimode reader equipped with a dual reagent injector module (Cytation 5, BioTek).

#### ROS and ATP measurements

For ROS measurements, 3D tendon constructs were removed from the bioreactor system and incubated with 8.7 μM CM-H_2_DCFDA (C6827, Thermo Fisher Scientific), a chloromethyl derivative of H_2_DCFDA with better retention in live cells than H_2_DCFDA, for 30 min in live-cell imaging solution and Hoechst 33342 (1:5000; 62249, Thermo Fisher Scientific) for 15 min then observed in 35-mm glass-bottom petri dishes by a Nikon A1Si confocal microscope with 60x water immersion objective equipped with a Tokai Hit stage top incubation system. To quantify CM-H_2_DCFDA fluorescence intensity, 3D constructs were moved from the bioreactor system to 5-ml tubes that were then incubated with 8.7 μM CM-H_2_DCFDA for 30 min, transferred to 96-well plates after washing three times with PBS, and then tested on a cell imaging multimode reader (Cytation 5, BioTek). CellTiter-Glo 3D Cell Viability Assay (Promega) was used for ATP measurements, which generates luminescent signals proportional to the amount of ATP. 3D tendon constructs were moved from the bioreactor system to 96-well plates. CellTiter-Glo 3D reagent was added to samples and mixed by shaking for 5 min and then incubating for 25 min at room temperature. Measurement was performed on a cell imaging multimode reader (Cytation 5, BioTek). Gen5 software (BioTek) was used for data analysis.

#### OCR and ECAR measurements

3D tendon constructs were removed from the bioreactor system and transferred to 96-well plates after three washes with PBS. The OCR of 3D tendon constructs was measured with MitoXpress Xtra Oxygen Consumption Assay (MX-200-4, Agilent) on 96-well plates and sealed with mineral oil, per the manufacturer’s protocol. ECAR measurement of 3D tendon constructs was performed using pH-Xtra Glycolysis Assay (PH-200-4, Agilent), per the manufacturer’s protocol. Before performing the pH-Xtra Glycolysis Assay, 3D tendon constructs were incubated in a CO_2_-free incubator for 2 hours at 37°C for CO_2_ purge. Dual-read ratiometric time-resolved fluorescence measurement was performed on a cell imaging multimode reader (Cytation 5, BioTek). Tendon constructs used for OCR or ECAR measurements were then collected. DNA was isolated using the DNeasy Blood & Tissue Kit (69506, QIAGEN) according to the manufacturer’s instructions. Isolated DNA was quantified by NanoDrop (Thermo Fisher Scientific). OCR and ECAR measurements from 3D tendon constructs were normalized by the DNA content, as previously published (*123*).

#### Integrative bioinformatics analysis across different mechanically stimulated datasets

A systematic search of the GEO database was done by the search formula showed in table S2. After manual selections to exclude duplicate and irrelevant datasets, 17 datasets were extracted. For some datasets, including samples with different stimulation durations, the samples with the longest simulation duration were selected as the treated group for analysis. Differential analysis was performed using limma package in R for each individual dataset (*124*). Then, the up-regulated and down-regulated genes were ranked by their fold change (FC) in each dataset. To integrate all 17 datasets, meta-analysis was conducted by the RobustRankAggreg R package (*125*), which is a standard method to minimize the outliers, errors, and noise among several datasets. A ubiquitous gene signature responsive to mechanical stimuli was explored. Genes with |log_2_FC| > 0.5 and adjusted *P* < 0.05 were considered as significant and were listed in the gene signature. To identify the functional roles of the gene signature indicated above, GO enrichment of biological process and molecular function was assessed using clusterProfiler R package. A *P* value of <0.05 and the false discovery rate (FDR) of <0.1 were considered statistically significant for GO enrichment. All analyses were conducted using R version 3.6.

#### Computational modeling of PM-ER TMS under stretch

A 3D continuum finite element model (FEM) of a thin patch of PM-ER tethered mechanotransmission system (TMS) with 60 μm by 22.5 μm by 50 nm (length by width by thickness) was developed to study the propagation of mechanical signals among subcellular components via PM-ER contact at a nanometer level. This PM-ER TMS model included the cell nucleus, nuclear lamina, PM, ER, and PM-ER tether structure and was modeled into shell elements. The mesh was generated into a total of 69,824 elements and 75,854 nodes. To simulate the stressed condition in actual experiments in a simplified way, a fixed support was applied to the edge of cell nucleus, and a 9% strain was applied to the PM. As the length of PM-ER TMS was 60 μm, a 5.4-μm uniaxial displacement was applied as 9% strain. As this model was used to reveal the force propagation over relatively brief periods, the utilization of mechanical properties in the model assumed that elasticity was time independent. To map the stress distribution on the mitochondria and lysosomes in PM-ER TMS under 6% strain stretching, a mitochondrion and a lysosome, each tethered to the ER by a tether structure, were further inputted into PM-ER TMS, and a uniaxial displacement of 3.6 μm was applied to the PM. Physical properties and morphological parameters of different cell components were previously experimentally measured (table S3). Simulation and calculation were performed using the ANSYS software package (ANSYS, Canonsburg, USA, v2021R1).

#### Statistical analysis

Comparisons of means between two groups were conducted by two-tailed Student’s *t* test. Comparisons of more than two groups were performed using one-way analysis of variance (ANOVA) with Dunnett’s post hoc or Tukey’s post hoc in accordance with comparison with the mean of every other column or a control column or two-way ANOVA with Sidak’s post hoc, unless stated otherwise. Statistical calculations were carried out and visualized by GraphPad Prism 8 (GraphPad) or OriginPro (version 2023b, OriginLab Corporation, Northampton, MA, USA). Each experiment consisted of at least three biological replicates. Each imaging experiment involved at least five different scanning areas on each biological replicate. Data are shown as the means ± SEM. Statistical significance was considered at α = 0.05, and *P* values were marked by asterisks (n.s., not significant; *P < 0.05, **P < 0.01, and ***P < 0.001).

## Supplementary Materials

### This PDF file includes:

Text S1 Figs. S1 to S23 Tables S1 to S3 Uncropped Western blots from fig. S21 References
