# Supplementary material for: External strain on the plasma membrane is relayed to the endoplasmic reticulum by membrane contact sites and alters cellular energetics
Source: Sci Adv. 2025 Jun 25;11(26):eads6132. doi: 10.1126/sciadv.ads6132 (PMC12190009; doi:10.1126/sciadv.ads6132)
Supplement: Supplementary file 1 — Text S1 Figs. S1 to S23 Tables S1 to S3 Uncropped Western blots from fig. S21 References [file sciadv.ads6132_sm.pdf]

Science Advances
AAAS

Supplementary Materials for

External strain on the plasma membrane is relayed to the endoplasmic reticulum by membrane contact sites and alters cellular energetics

Ziming Chen *et al.*

Corresponding author: Ming Hao Zheng, minghao.zheng@uwa.edu.au

Sci. Adv. **11**, eads6132 (2025)
DOI: 10.1126/sciadv.ads6132

This PDF file includes:

Text S1
Figs. S1 to S23
Tables S1 to S3
Uncropped Western blots from fig. S21
References

Text S1. Computational modeling suggests that detethering the ER from the PM alleviates mechanical stress in the ER under stretching

Based on our observation on the changing of ER tension after cyclic strain with the intact or reduced PM-ER contacts, we proposed that detethering of the ER from the PM can hinder the propagation of mechanical force from the PM to the ER and thus lead to lower adaptive membrane tension in the latter (**Fig. 3O**). To speculate on the effect of PM-ER contacts on the ER mechanical homeostasis under stretching, we employed a computer-aided cell modeling method. To simulate the force propagation from the PM to the ER, we first developed a finite element model (FEM) for a thin patch of PM-ER tethered mechanotransmission system (PM-ER TMS) (**Fig. S19A**). We simplified the model and did not account for the inherent nature of the membrane system, including its nonequilibrium state, high anisotropy and viscoelasticity. We included the cell nucleus, nuclear lamina, PM, ER and the PM-ER tether structure in this continuum model (**Fig. S19B**). The ER was organized into ER sheets and interlinked ER tubules connected at three-way junctions as matrices, with a ratio of ER sheets to total ER at 20 percents(*120, 126*). The thickness of PM-ER TMS was set to 50 nm, similar to the thickness of the ER lumen(*127*). The PM-ER tether structure was initially arranged to an extent of 150 nm and the number of two based on the PM occupancy by PM-ER contacts at around 1%(*53, 87, 128*). Physical properties and morphological parameters of different cell components were based on previous studies (**Table S1**).

We then simulated a uniaxial 9% strain to stretch the cell as applied for stressed condition in actual experiments, which enabled the analysis of force propagation in PM-ER TMS (**Fig. S19C**). As we observed that *Stim1* knockdown reduced both the number (**Fig. 5E**,**F**) and the extent of PM-ER contact sites (**Fig. 8A**,**B**), we simulated three models with different PM-ER tether structures in PM-ER TMS to dissect the effect of number and extent of PM-ER contact sites on force propagation, including Model 1 with natural PM-ER tether structures as described above, Model 2 with reduced number of PM-ER tether structure from two to one, and Model 3 with reduced number of PM-ER tether structure from two to one and reduced extent of PM-ER tether structure from 150 nm to 75 nm (**Fig. S19D**). The total ER was meshed into 60,162 nodes. We selected one region from ER tubules, Region 1, consisting of 348 nodes and one region from ER sheets, Region 2, consisting of 242 nodes for statistic quantification (**Fig. S19E**,**F**). We then defined the deformation as the displacement of each node before and after stretching, calculated by the square root of the total of the square of X, Y and Z direction (**Fig. S19D**,**G**,**H**). Compared to Model 1 with natural PM-ER tether structures under a 9% strain on PM, analysis showed decreased deformation on both ER tubules and ER sheets in Model 2 with reduced number of PM-ER tether structures (**Fig. S19G**,**H**). By comparison between Model 2 and Model 3, FEM showed that reduced extent of PM-ER tether structure decreased the deformation on both ER tubules and ER sheets (**Fig. S19G**,**H**). As expected, compared to Model 1 and Model 2, least deformation was demonstrated in Model 3 on both ER tubules and ER sheets (**Fig. S19G**,**H**). We subsequently calculated von Mises stress, characterized as the equivalent uniaxial tensile stress that would generate the same distortion energy as the actual composite applied stresses, based on the principal stresses and in accordance with the Von Mises Stress equation. Paired *t*-tests at each node in Region 1 and Region 2 revealed a significantly lower von Mises stress on both ER tubules and ER sheets could be seen in Model 2 compared to Model 1; in Model 3 compared to Model 2; and in Model 3 compared to Model 1, indicating mechanical stress was significantly diminished on each node under a 9% strain on the PM when the number or extent of PM-ER tethers are lower (**Fig. S19I**). Consistently, calculation of average von Mises stress in total ER demonstrated that Model 2 relieved 37.3 Pa stress in the ER compared to Model 1; Model 3 relieved 18.7 Pa stress in the

ER compared to Model 2; and Model 3 relieved 56 Pa stress totally in the ER compared to Model 1, indicating that a decrease of either the number or extent of PM-ER tether structures alleviate mechanical stress on the ER under stretching (**Fig. S19I**). Consequently, our FEM simulation suggests the critical role of the PM-ER tether structure in force propagation under external mechanical strain.

Our data showed that the adaptive response of membrane tension to external mechanical strain occurred in the ER, but neither in mitochondria nor lysosomes (**Fig. 1F**,**G**). To assess stress distribution on mitochondria and lysosomes tethered to the ER in the PM-ER TMS under mechanical stretching, we inputted a mitochondrion and a lysosome, each connected to the ER by a tether structure, into our simulation of Model 1 with natural PM-ER tether structures. We set the mechanical stimulus at 6% strain on the plasma membrane to mimic the conditions of our experimental setup. The computational analysis revealed that von Mises stress experienced by the mitochondrion and lysosome was significantly lower than that on the ER (**Fig. S19J**). The simulation suggests that while the ER's deformation may facilitate the movement of these two tethered organelles, the mitochondria and lysosomes themselves do not undergo substantial stress due to their motility and lack of fixed boundaries. This model offers a possible explanation for the observed absence of significant adaptive changes of membrane tension on the mitochondria and lysosomes following stretching.

Together, these computational analyses suggested that detethering the ER from the PM alleviated mechanical stress in the ER under stretching, consistent with our experimental observations.

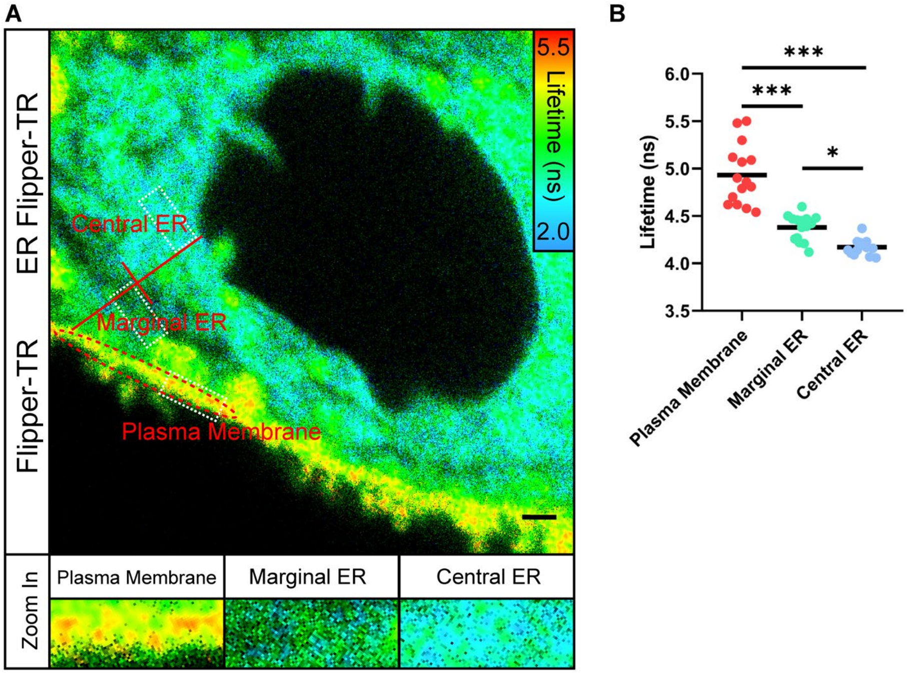

Fig. S1. Co-staining of PM and ER tension probes. (A) Representative FLIM images of mouse tendon cells co-stained with Flipper-TR and ER Flipper-TR. According to the midpoint of a line from the nuclear envelope to the PM and perpendicular to the tangent of the surface of the nucleus, the endoplasmic reticulum is divided into marginal ER and central ER. Lower panels are the enlargements of areas of the PM, marginal ER and central ER from the upper panel (white rectangles). **(B)** Distribution of fluorescence lifetime selecting PM, marginal ER or central ER as the ROI in tendon cells (*n*=15 cells per group, each with at least two ROIs, from three independent experiments). ***$P < 0.001$; *$P < 0.05$ by one-way analysis of variance (ANOVA). ROI, region of interest.

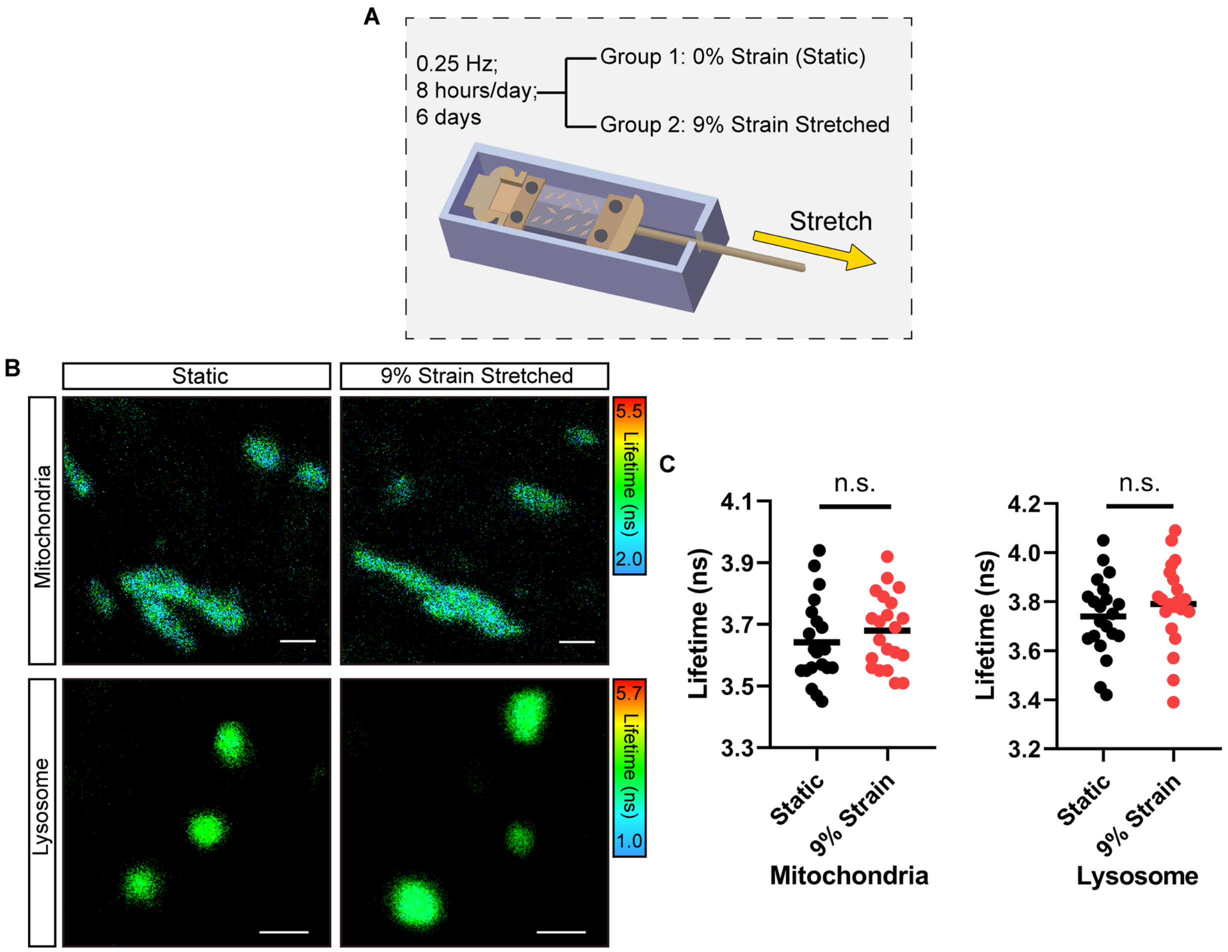

Fig. S2. 9% cyclic strain does not alter tension of mitochondria and lysosomes. (A) A schematic diagram illustrating the experimental approach used to mechanically stimulate monolayers of mouse tendon cells under 9% strain. **(B)** Representative FLIM images of mitochondria stained with the mitochondria-targeted fluorescent tension probe Mito Flipper-TR (upper), and lysosomes stained with the lysosome-targeted fluorescent tension probe Lyso Flipper-TR (lower), in tendon cells with or without 9% cyclic strain. Scale bars, 1 µm. **(C)** Distribution of fluorescence lifetime of Mito Flipper-TR (left) and Lyso Flipper-TR (right) in static and 9% strain-stretched group (*n*=21 cells per group, each with at least three ROIs, from three independent experiments). Line marks the mean of the distribution. n.s., not significant by Student's *t*-test.

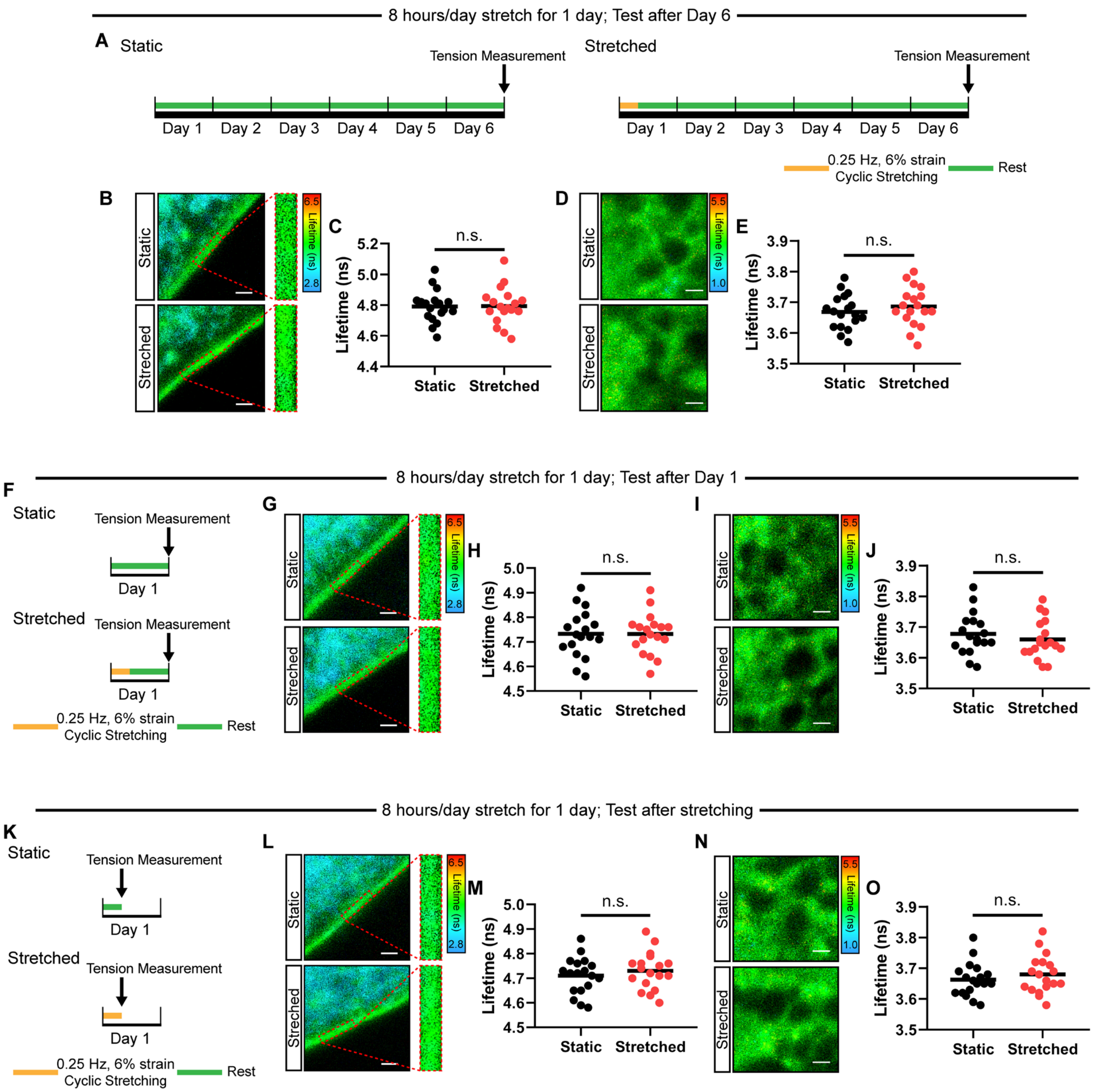

Fig. S3. Cyclic strain in the short term does not increase PM tension and ER tension. (A) A schematic diagram illustrating the experimental approach used to mechanically stimulate monolayers of mouse tendon cells for 1 day with tension measurements taken on the sixth day. **(B**,**C)** Representative FLIM images of PM tension (**B**) probed by the PM-targeted fluorescent tension probe Flipper-TR in tendon cells with or without cyclic mechanical stimulation using the experimental approach showed in (**A**), and the corresponding distribution of fluorescence lifetime of Flipper-TR (**C**) after selecting the PM as the region of interest (ROI) (n=18 cells per group, each with at least two ROIs, from three independent experiments). **(D**,**E)** Representative FLIM images of ER tension (**D**) probed by ER Flipper-TR in tendon cells with or without cyclic mechanical stimulation using the experimental approach showed in (**A**), and the corresponding distribution of fluorescence lifetime of ER Flipper-TR (**E**) (n=18 cells per group, each with at least two ROIs, from three independent experiments). **(F)** A schematic diagram illustrating the experimental approach used to mechanically stimulate monolayers of mouse tendon cells for 1 day with tension

measurements taken after the day. **(G,H)** Representative FLIM images of PM tension (**G**) probed by Flipper-TR in tendon cells with or without cyclic mechanical stimulation using the experimental approach showed in (**F**), and the corresponding distribution of fluorescence lifetime of Flipper-TR (**H**) after selecting the PM as the region of interest (ROI) (*n*=18 cells per group, each with at least two ROIs, from three independent experiments). **(I,J)** Representative FLIM images of ER tension (**I**) probed by ER Flipper-TR in tendon cells with or without cyclic mechanical stimulation using the experimental approach showed in (**F**), and the corresponding distribution of fluorescence lifetime of ER Flipper-TR (**J**) (*n*=18 cells per group, each with at least two ROIs, from three independent experiments). **(K)** A schematic diagram illustrating the experimental approach used to mechanically stimulate monolayers of mouse tendon cells for 8 hours with tension measurements taken immediately after the 8-hour stretching. **(L,M)** Representative FLIM images of PM tension (**L**) probed by Flipper-TR in tendon cells with or without cyclic mechanical stimulation using the experimental approach showed in (**K**), and the corresponding distribution of fluorescence lifetime of Flipper-TR (**M**) after selecting the PM as the region of interest (ROI) (*n*=18 cells per group, each with at least two ROIs, from three independent experiments). **(N,O)** Representative FLIM images of ER tension (**N**) probed by ER Flipper-TR in tendon cells with or without cyclic mechanical stimulation using the experimental approach showed in (**K**), and the corresponding distribution of fluorescence lifetime of ER Flipper-TR (**O**) (*n*=18 cells per group, each with at least two ROIs, from three independent experiments). Each right panel in (**B**,**G**,**L**) is an enlargement of the dashed red boxed area in the corresponding left panel. Line marks the mean of the distribution. Scale bars, 1 μm. n.s., not significant by Student's *t*-test.

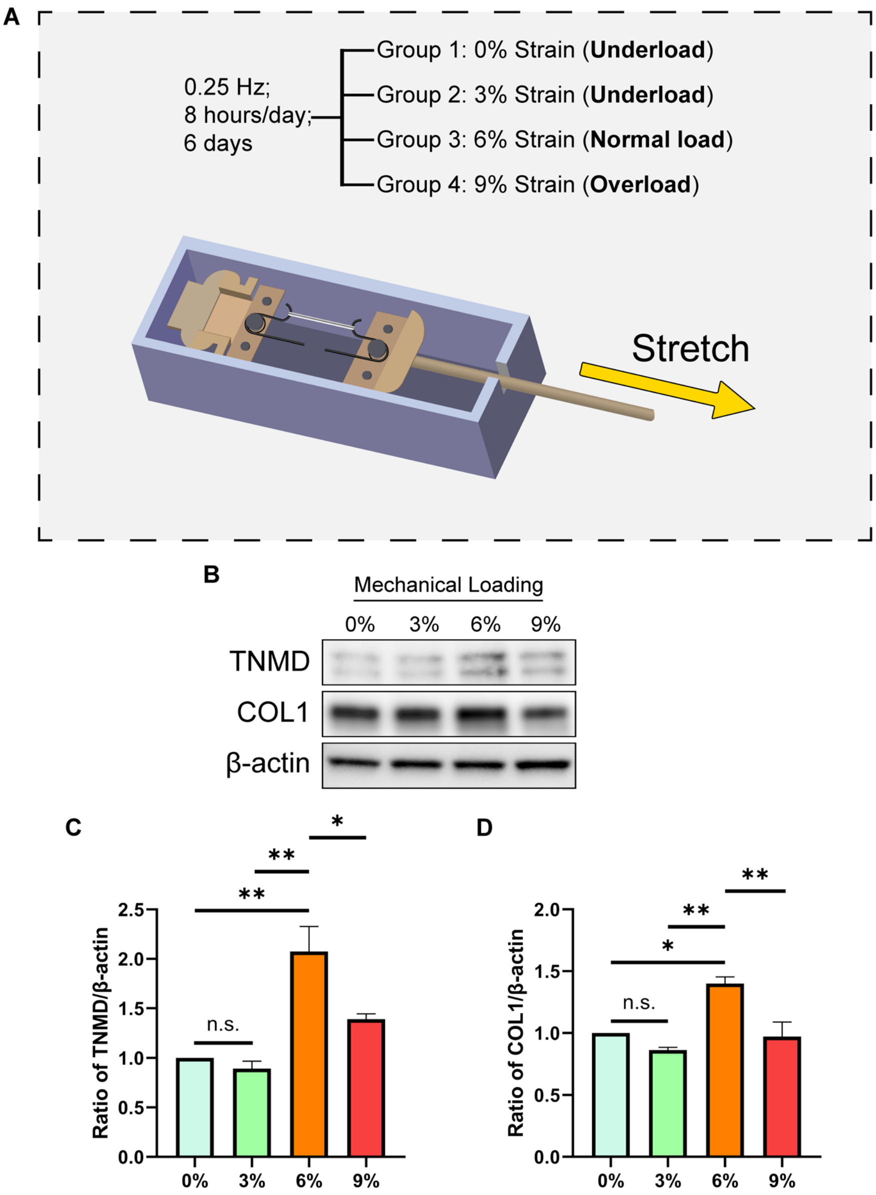

Fig. S4. A 3D uniaxial cyclic stretching system closely mimics the mechanical environment of underloading, normal loading and overloading. (A) A schematic diagram of mechanical stimulation of 3D tendon constructs stimulated with underloading (0% or 3% strain), normal loading (6% strain) or overloading (9% strain). **(B-D)** Representative immunoblot analysis of TNMD and COL1 expression in tendon constructs with the indicated loading regimes (**B**), and their quantitation (**C**,**D**). The quantitative analysis is based on three biological replicates from three independent experiments. β-Actin expression was measured as the internal control. $**P < 0.01$; $*P < 0.05$; n.s., not significant by one-way ANOVA.

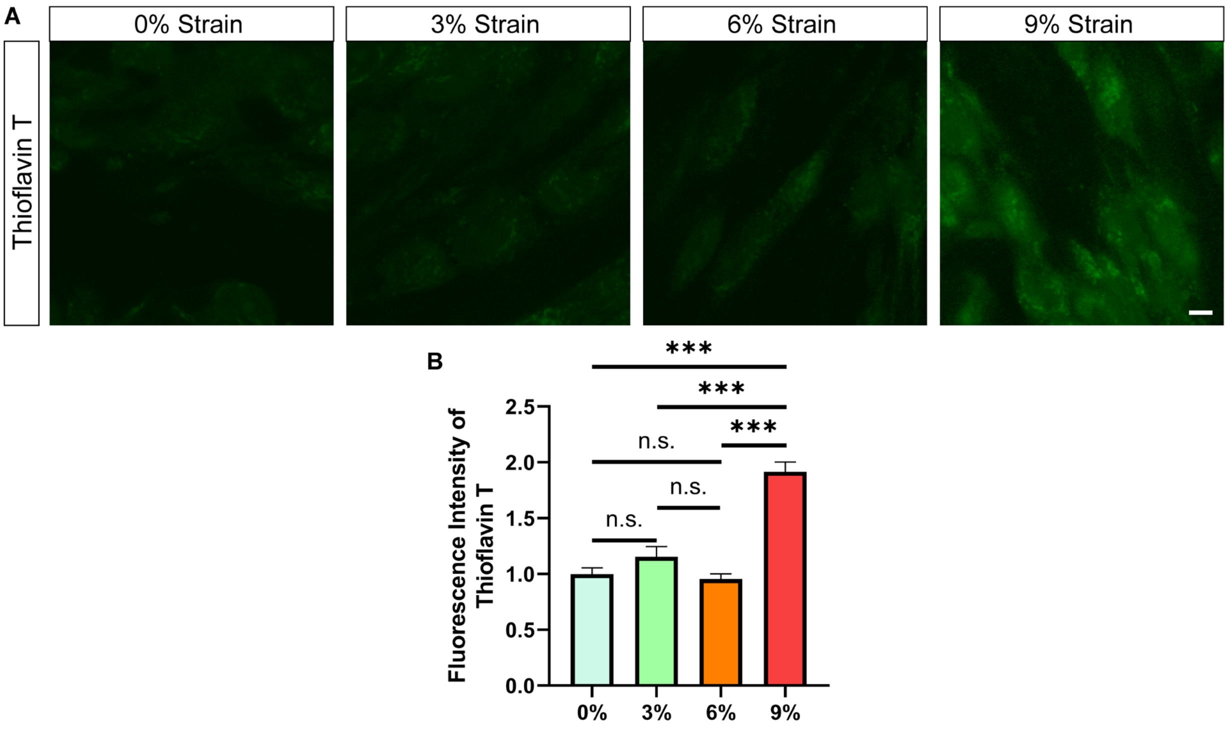

Fig. S5. Fluorescence intensity of ThT staining of 3D tendon cell constructs increases in the 9% strain group, compared to 0%, 3% or 6% strain groups. (A,B) Representative confocal images of ThT staining to 3D tendon cell constructs after 0%, 3%, 6%, or 9% cyclic strain (**A**), and its corresponding fluorescence intensity analysis (**B**) (*n*=15 scanning areas from three tendon constructs per group, each with at least two ROIs, from three independent experiments). Scale bar, 5 µm. ***$P < 0.001$; *$P < 0.05$ by one-way ANOVA.

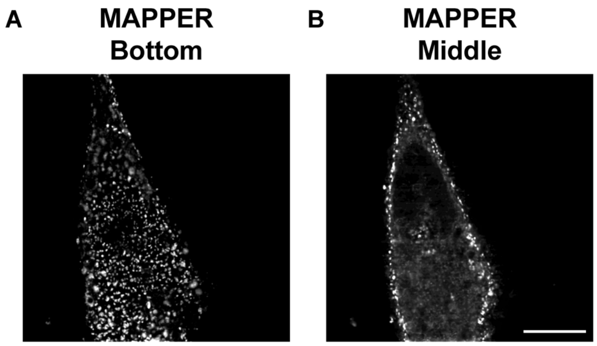

Fig. S6. PM-ER junctions exist in mouse tendon cells. (A,B) Live-cell confocal images of the bottom (**A**) and middle (**B**) sections of a GFP-MAPPER (marker for PM-ER junctions)-expressing mouse tendon cell. Scale bar, 10 μm.

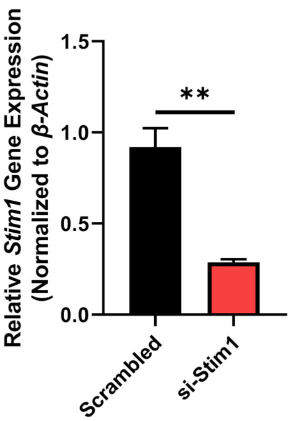

Fig. S7. Partial knockdown of *Stim1* in tendon cells receiving strain. RT-qPCR analysis of *Stim1* in mouse tendon cells cultured in 2D cyclic stretching environment with 6% strain for 6 days and transfected with scrambled siRNA or *Stim1*-siRNA. Three biological replicates from three independent experiments were conducted. $^{**}P < 0.01$ by Student's *t*-test.

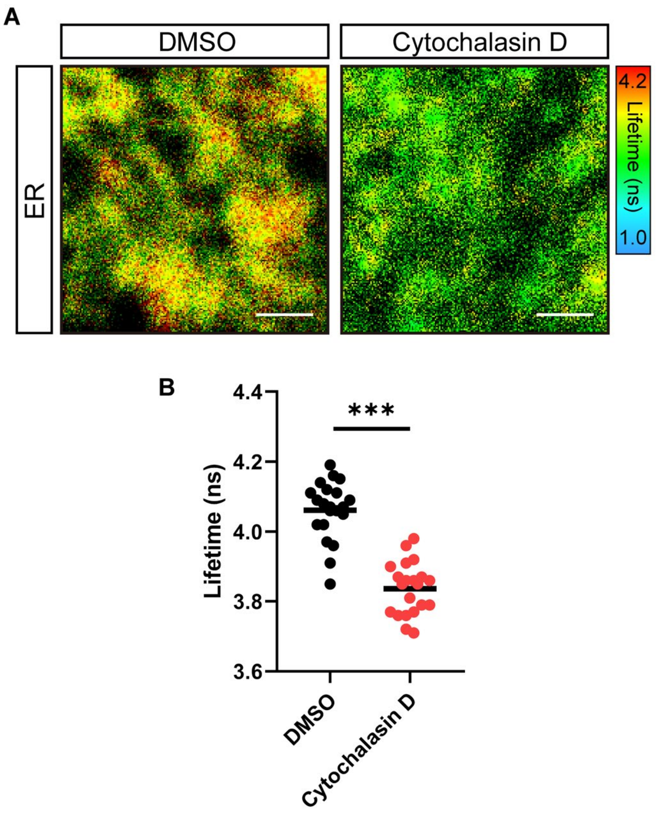

Fig. S8. Actin cytoskeleton is essential for maintaining ER tension. (A) Representative FLIM images of ER Flipper-TR-stained tendon cells treated with carrier (DMSO) or cytochalasin D. Scale bar, 1 μm. **(B)** Distribution of fluorescence lifetime of ER Flipper-TR in tendon cells cultured in glass-bottom petri dishes and treated with or without cytochalasin D (n=21 cells per group, each with at least two ROIs, from three independent experiments). Line marks the mean of the distribution. ***$P < 0.001$ by Student's t-test.

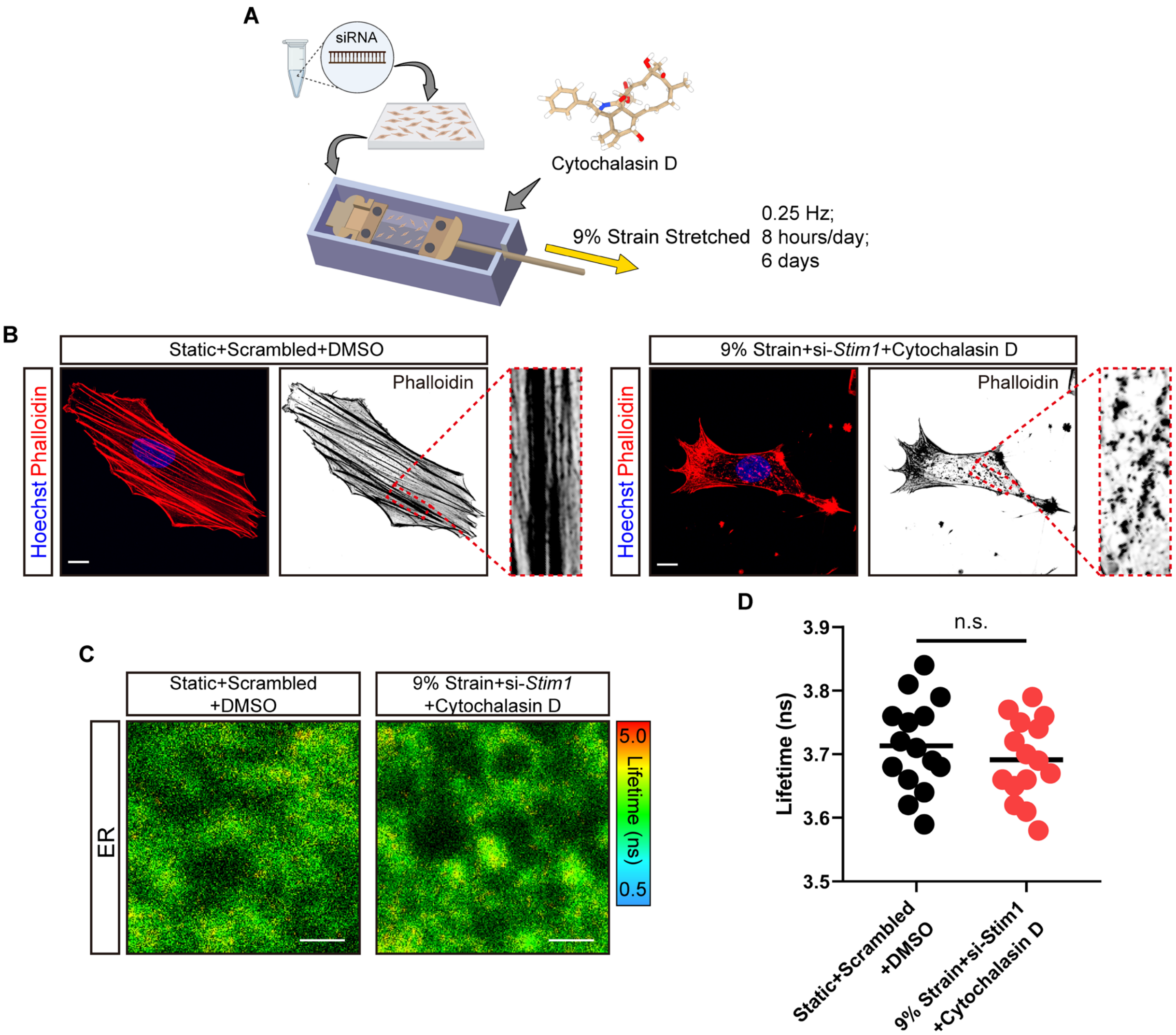

Fig. S9. Effects of cytochalasin D on ER tension in *Stim1* partial knockdown cells under 9% strain. (A) A schematic diagram illustrating the experimental setup used to mechanically stimulate monolayers of mouse tendon cells transfected with *Stim1*-siRNA and treated with cytochalasin D under 9% cyclic strain. **(B)** Representative confocal images showing Phalloidin-labeled F-actin (red) and Hoechst-labeled nuclei (blue) in tendon cells transfected with scrambled siRNA and treated with DMSO under static culture, as well as in tendon cells transfected with *Stim1*-siRNA and treated with cytochalasin D under 9% cyclic strain. Right panels display the F-actin channel along with magnified views of the regions outlined by the red dashed rectangles. **(C,D)** Representative FLIM images of ER Flipper-TR-stained tendon cells transfected with scrambled siRNA and treated with DMSO under static culture, and tendon cells transfected with *Stim1*-siRNA and treated with cytochalasin D under 9% cyclic strain (**C**). Scale bar, 1 µm. And corresponding distribution of fluorescence lifetime of ER Flipper-TR (**D**) (n=15 cells per group, each with at least two ROIs, from three independent experiments). Line marks the mean of the distribution. n.s., not significant by Student's t-test.

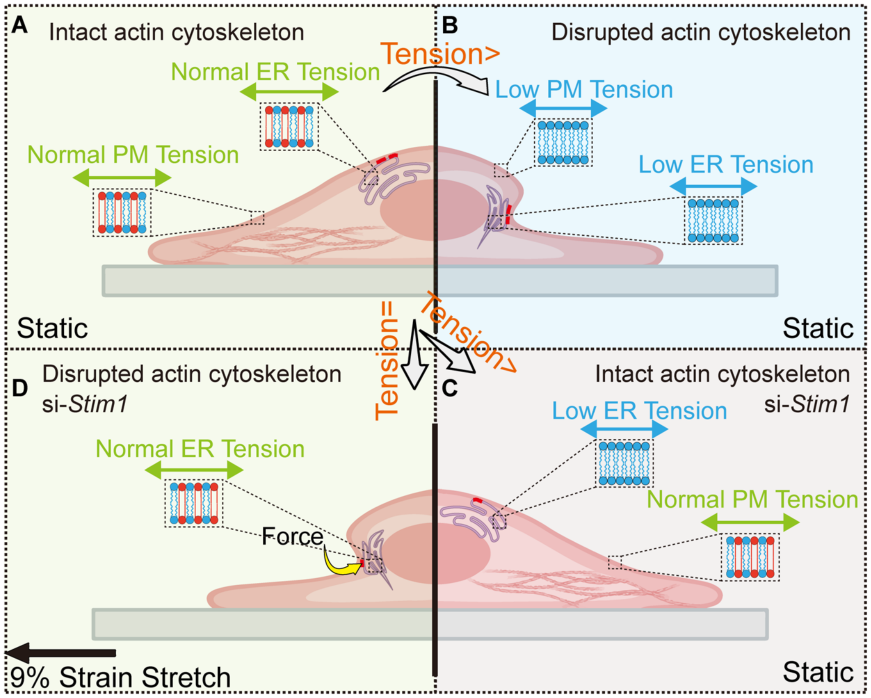

Fig. S10. Schematic summary of the contributions of the actin cytoskeleton in maintaining PM and ER tension, along with hypothetical models. (A-C) Disruption of the actin cytoskeleton reduces both PM and ER tension (**B**), while detethering the ER from the PM via si-*Stim1* transfection reduces ER tension without affecting PM tension (**C**) under static culture conditions, compared to control cells (**A**). **(D)** Under 9% strain, the combined disruption of the actin cytoskeleton and si-*Stim1* transfection resulted in ER tension comparable to that of control cells under static conditions. Since si-*Stim1* treatment reduced ER tension under static culture conditions to levels lower than those in control cells (**B**), this suggests that external cyclic forces may still influence ER tension, potentially through remaining mechanotransduction pathways, such as residual PM-ER contact sites or other yet-to-be-identified structures.

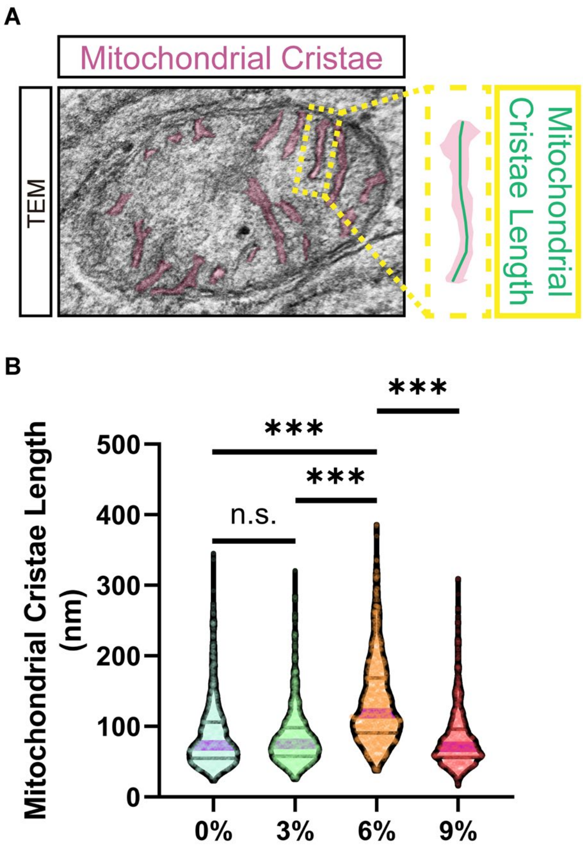

Fig. S11. Mechanical loading dose-dependently affects mitochondrial cristae. (A) Method for quantitative analysis of mitochondrial cristae length. Mitochondrial cristae are highlighted in purple color in a pseudocoloured transmission electron microscopy image. Right panel is the zoom of the yellow dashed box in the corresponding left panel, with the crista long axis delineated by a green line, which was used for cristae length measurements. **(B)** The corresponding quantitative analysis to mitochondrial cristae length of 3D tendon constructs receiving 0%, 3%, 6% or 9% cyclic strain (0% strain n=809, 3% strain n=1019, 6% strain n=720, 9% strain n=693; n, mitochondrial cristae from three independent experiments). ***$P < 0.001$; n.s., not significant by Kruskal-Wallis test. TEM, transmission electron microscope. Violin plot presents the median and quartiles.

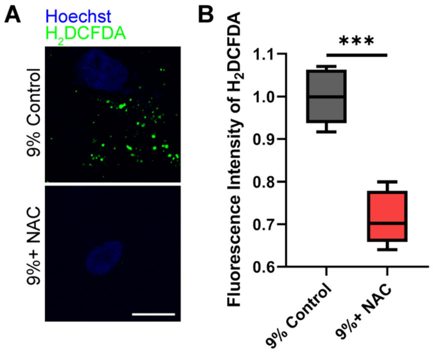

Fig. S12. Validation of the effect of NAC treatment on the clearance of ROS in 3D tendon constructs during overloading. (A,B) Representative confocal fluorescence images of H_2DCFDA-labeled ROS in 3D tendon constructs receiving 9% strain supplied with or without NAC (**A**), and the corresponding fluorescence intensity quantification of H_2DCFDA (**B**) (*n*=5 biological replicates from three independent experiments). Scale bar, 10 µm. ***$P < 0.001$ by Student's *t*-test.

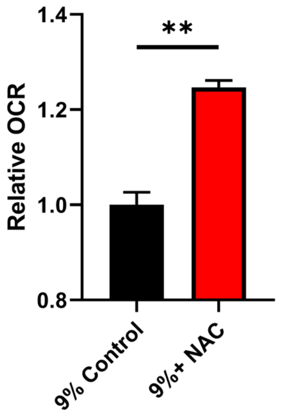

Fig. S13. The effect of NAC treatment on mitochondrial respiration in 3D tendon constructs during overloading. Mitochondrial respiration of 3D tendon constructs receiving 9% strain supplied with or without NAC (*n*=3 biological replicates from three independent experiments) measured by OCR. $^{**}P < 0.01$ by Student's *t*-test. Error bars stand for standard error of the mean.

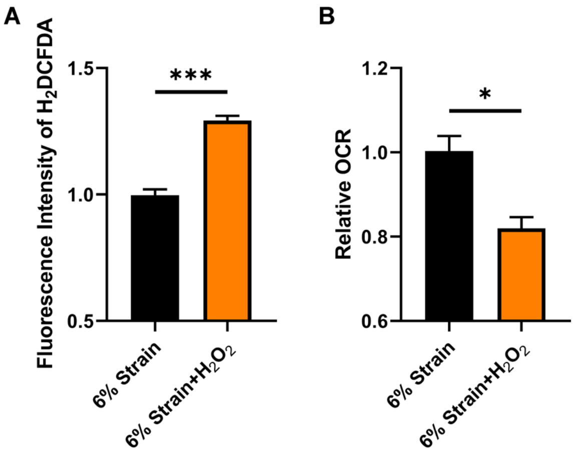

Fig. S14. The effect of H_2O_2 treatment on mitochondrial respiration in 3D tendon constructs under 6% strain. (A) Quantification of fluorescence intensity of H_2DCFDA-labeled ROS in 3D tendon constructs subjected to 6% strain with or without H_2O_2 treatment (*n*=3 biological replicates from three independent experiments). **(B)** Mitochondrial respiration of 3D tendon constructs receiving 6% strain supplied with or without H_2O_2 (*n*=3 biological replicates from three independent experiments) measured by OCR. ***$P < 0.001$; *$P < 0.1$ by Student's *t*-test. Error bars stand for standard error of the mean.

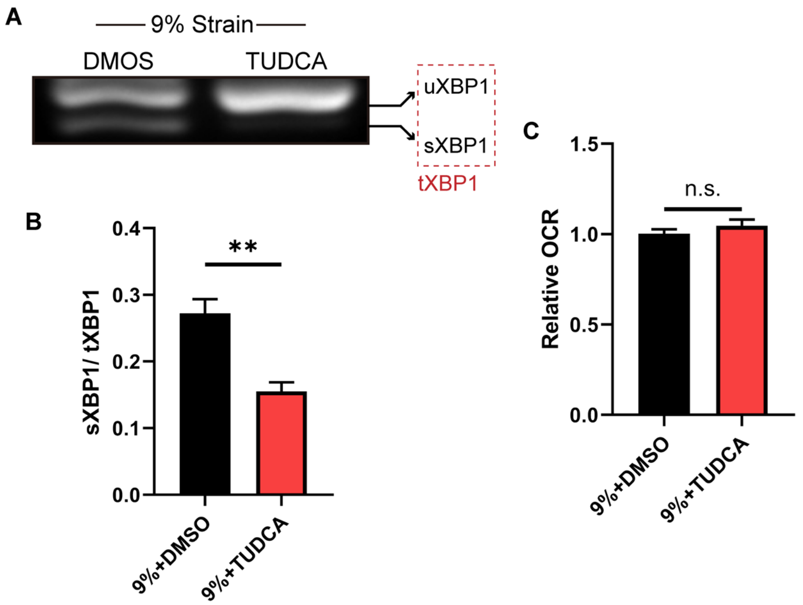

Fig. S15. ER stress does not affect mitochondrial respiration under 9% strain. (A,B) RT-PCR analysis (**A**) and its quantification (**B**) of XBP1 splicing of 3D tendon constructs receiving 9% strain supplied with or without TUDCA (three biological replicates from three independent experiments). **(C)** Mitochondrial respiration of 3D tendon constructs receiving 9% strain supplied with or without TUDCA (n=3 biological replicates from three independent experiments) measured by OCR. uXBP1, unspliced XBP1; sXBP1, spliced XBP1; tXBP1, total XBP1. **$P < 0.01$; not significant by Student's t-test. Error bars stand for standard error of the mean.

A

Organ /Tissue	Cell Type	Species	GEO Accession
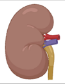	Podocyte<br>Podocyte	Mouse<br>Mouse	GSE66336<br>GSE99988
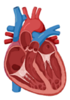	Cardiomyocyte<br>Fibroblast<br>Cardiomyocyte<br>Ventricular cell<br>Cardiomyocyte<br>Ventricular myocyte	Rat<br>Rat<br>Rat<br>Rat<br>Rat<br>Rat	GSE2032<br>GSE2032<br>GSE5996<br>GSE48342<br>GSE92956<br>GSE107551
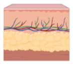	Skin fibroblast<br>Dermal fibroblast	Mouse<br>Human	GSE3486<br>GSE10125
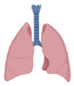	Lung type II epithelial cell	Rat	GSE3541
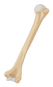	MLO-Y4 osteocyte-like cell	Mouse	GSE42874
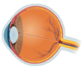	Retinal Muller cell<br>Corneal fibroblast	Rat<br>Mouse	GSE43516<br>GSE3486
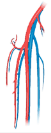	Umbilical vein endothelial cell	Human	GSE43582
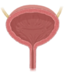	Bladder smooth muscle cell	Human	GSE1595
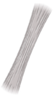	Tendon fibroblast	Mouse	GSE3486

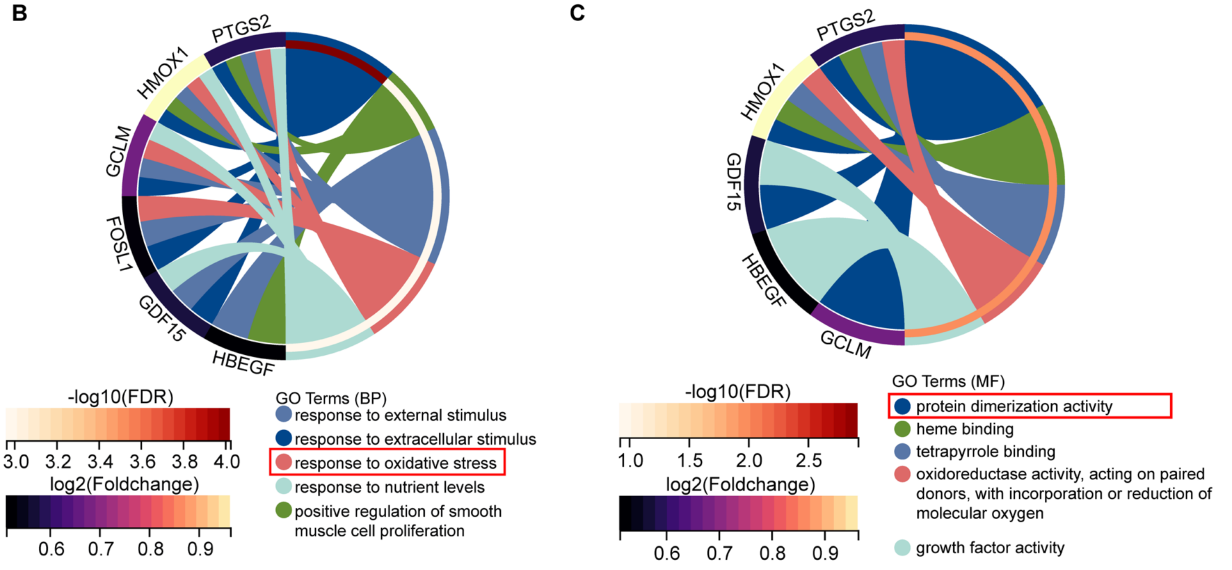

Fig. S16. Integrative bioinformatic analysis reveals ubiquitously mechanoresponsive cellular activities. (A-C) 17 datasets across 9 tissues/organs from the GEO database (**A**) were extracted for integrative bioinformatic analysis to generate chord plots of the top 5 ranked GO terms for BP

(**B**) and MF (**C**) (by *p* value) for ubiquitously mechanoresponsive cellular activities. The genes from the ubiquitous gene signature responsive to mechanical stimuli are linked to corresponding GO BP or MF terms via colored ribbons. $-\log_{10}$FDR and $\log_2$(Foldchange) are color-coded. FDR, false discovery rate; GEO, Gene Expression Omnibus; GO, Gene ontology; BP, Biological Process; MF, Molecular Function.

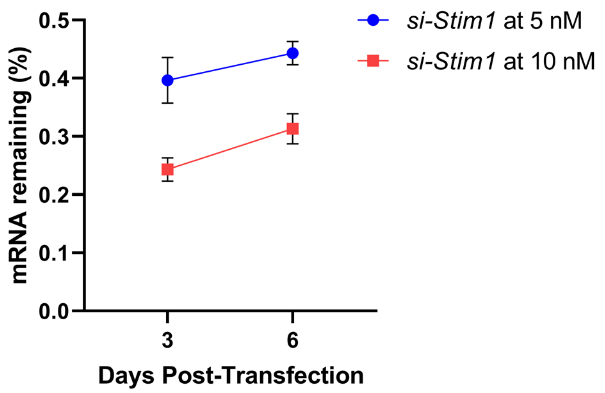

Fig. S17. Knockdown of *Stim1* in 3D tendon constructs with 9% cyclic strain. Remaining mRNA levels of *Stim1* as detected by RT-qPCR in 3D tendon constructs cultured in 9% cyclic strain environment and transfected with 5 nM (blue) or 10 nM (red) of *Stim1*-siRNA and then lysed at day 3 or day 6 post-transfection. Presented data are expressed relative to data from 3D tendon constructs transfected with scrambled siRNA cultured in 9% cyclic strain environment. Three biological replicates from three independent experiments were conducted.

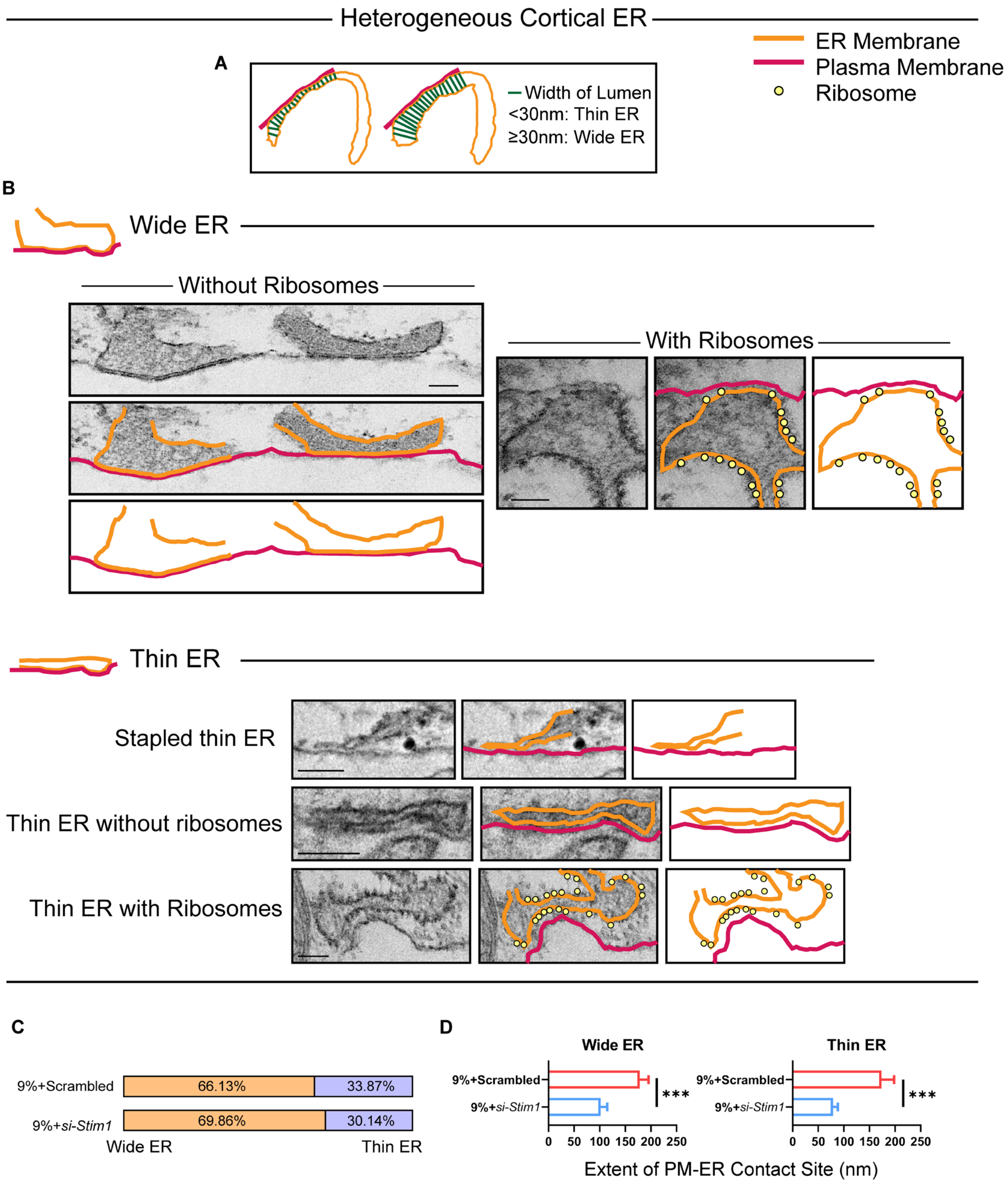

Fig. S18. Representative transmission electron microscopy images of heterogenous cortical ER in 3D tendon constructs. (A) Schematic diagram illustrating the definition of wide ER and thin ER. **(B)** Representative transmission electron microscopy images of heterogenous wide ER and thin ER. Pseudocoloured images highlighting PM (red), ER (orange), and ribosomes (yellow) in different colors. Stapled thin ER refers to a form of the thin ER with an extremely narrow lumen, in which the two opposite faces of the cisternae are closely apposed, resembling a "stapled" structure. **(C,D)** Quantification to the portion (**C**) and the extent of wide ER and thin ER (**D**) in 3D

tendon constructs transfected with scrambled siRNA or *Stim1*-siRNA, and cultured in 9% cyclic strain environment (9%+Scrambled in wide ER *n*=41, 9%+si-*Stim1* in wide ER *n*=51, 9%+Scrambled in thin ER *n*=21, 9%+si-*Stim1* in thin ER *n*=22; *n*, contact sites from three independent experiments). Scale bars, 100 nm. ***P < 0.001 by Student's *t*-test.

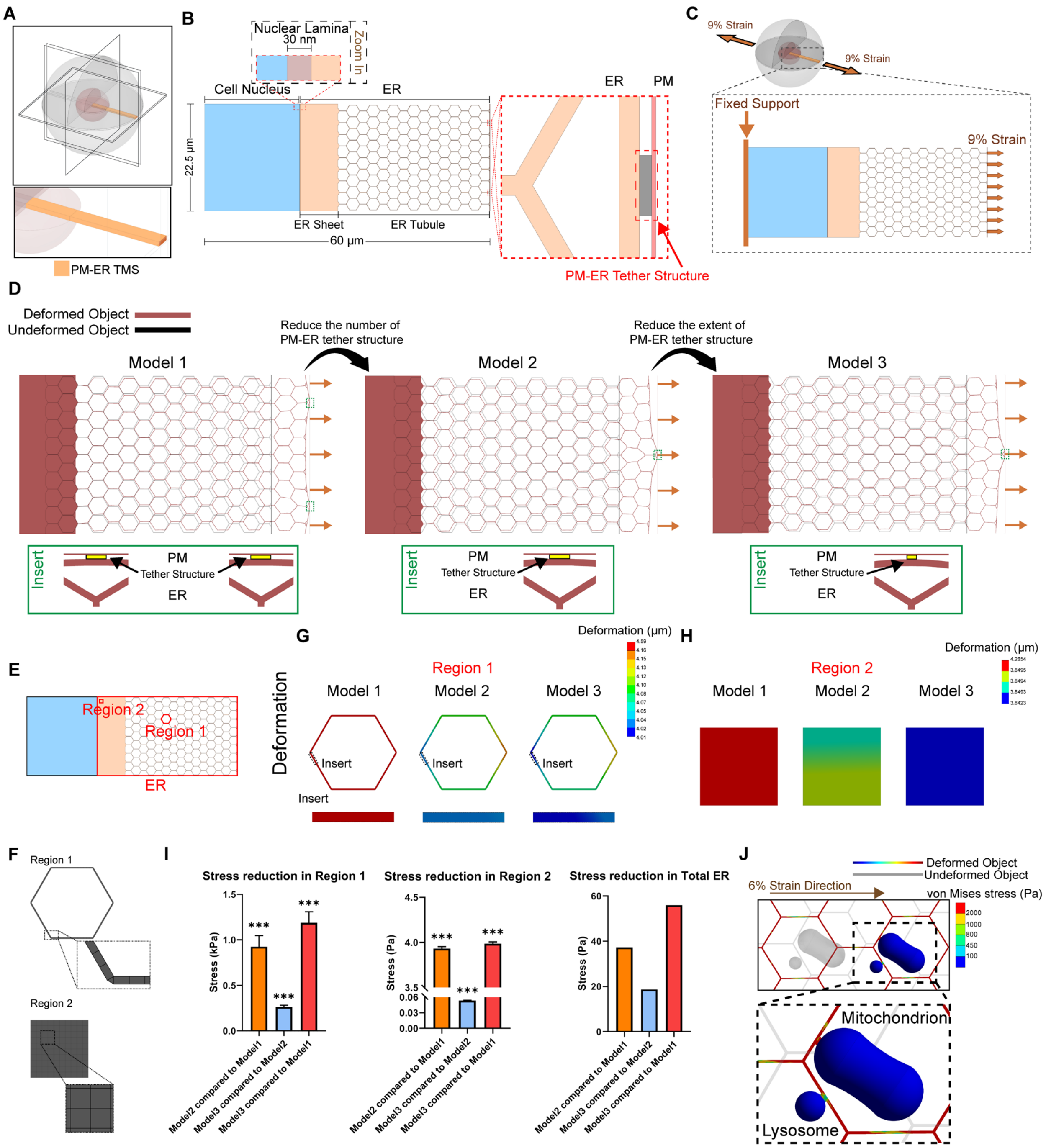

Fig. S19. Detethering the ER from the PM alleviates mechanical stress in the ER under stretching. (A) A schematic diagram illustrating a thin patch of PM-ER tethered mechanotransmission system (PM-ER TMS) in a cell. Bottom panel is the zoom of the PM-ER TMS in the top panel. **(B)** A finite element model (FEM) of a patch of PM-ER TMS includes the cell nucleus, nuclear lamina (indicated by the top red dashed insert and colored in brown), PM, ER, and the PM-ER tether structure (indicated by the right red dashed insert and colored in grey). **(C)** To simulate a uniaxial 9% strain to PM-ER TMS as applied for stressed condition in actual experiments, a fixed support was applied to the edge of cell nucleus, and a 9% strain was applied to the PM as indicated by the bottom black dashed box. **(D)** Three models were computerized as

in the upper panel. The lower panel shows the enlargement of PM-ER tether structure in the green dashed boxed area in the corresponding upper panel. Model 1 with two PM-ER tether structures, and the extent of each PM-ER tether structure is 150 nm. Model 2 with reduced number of PM-ER tether structure from two to one, and the extent of each PM-ER tether structure is 150 nm. Model 3 with reduced number of PM-ER tether structure from two to one and reduced extent of PM-ER tether structure from 150 nm to 75 nm. The undeformed PM-ER TMS before stretching is shown in black, and the simulated deformed PM-ER TMS after stretching by 9% strain is shown in red. **(E**,**F)** Two regions from the ER were chosen for detailed display (**E**), including Region 1 from ER tubules consisting of 348 nodes, and Region 2 from ER sheets consisting of 242 nodes (**F**). Inserts in (**F**) show the mesh. **(G**,**H)** Deformation in Region 1 (**G**) and Region 2 (**H**) after stretching. The lower panels in (**G**) show the enlargement in the dashed boxed area in the corresponding upper panels. **(I)** Stress reduction after stretching in Region 1, Region 2 and total ER by comparison among Model 1, Model 2 and Model 3. **(J)** Stress distribution in PM-ER TMS featuring a mitochondrion (cylinder) and a lysosome (sphere) tethered to the ER, subjected to a uniaxial 6% strain, as described in (**C**) with a modification to the strain level. The undeformed situation before stretching is shown in grey, and the simulated deformed PM-ER TMS after stretching by 6% strain is color-coded according to von Mises stress. Lower panel is an enlargement of the black dashed boxed area in the upper panel. ***$P < 0.001$ by paired *t*-test. Error bars stand for standard error of the mean.

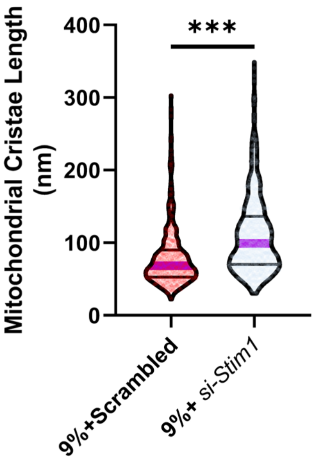

Fig. S20. Partial knockdown of *Stim1* rescues the integrity of mitochondrial cristae impaired by overloading. Quantitative analysis of mitochondrial cristae length in 3D tendon constructs transfected with scrambled siRNA or *Stim1*-siRNA and cultured under 9% cyclic strain (9%+Scrambled *n*=415, 9%+si-*Stim1* *n*=684; *n*, contact sites from three independent experiments). ***$P < 0.001$ by Mann-Whitney test.

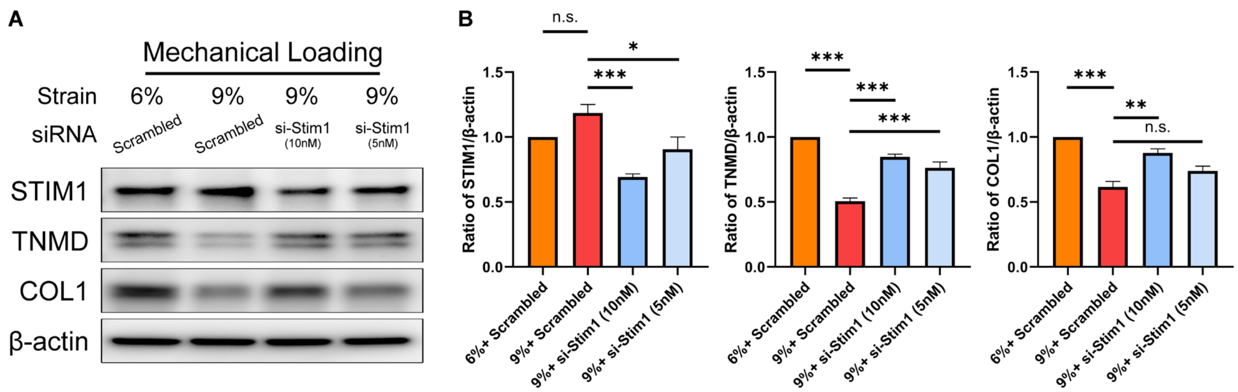

Fig. S21. Partial knockdown of the PM-ER tether protein *Stim1* can rescue tendon cell function impaired by overloading. (A,B) Representative immunoblot analysis of STIM1, TNMD and COL1 expression (**A**), and the quantitative analysis of immunoblot assays (**B**) presented as three biological replicates from three independent experiments, in 3D tendon constructs receiving 6% strain for 6 days, transfected with scrambled siRNA, or 9% strain transfected with scrambled siRNA, 5 nM *Stim1*-siRNA, or 10 nM *Stim1*-siRNA. β-Actin expression was measured as the internal control. ***$P < 0.001$; **$P < 0.01$; *$P < 0.05$; n.s., not significant by one-way ANOVA.

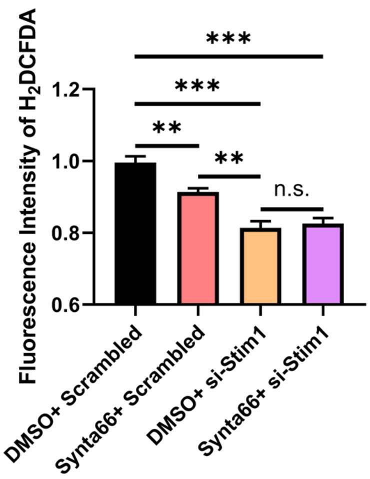

Fig. S22. Contributions of SOCE and PM-ER physical tethering to ROS in tendon constructs during overload. Quantification of fluorescence intensity of H_2DCFDA-labeled ROS in 3D tendon constructs transfected with scrambled siRNA or *Stim1*-siRNA, and further treated with DMSO (carrier) or Synta66 (10 μM) following 9% cyclic strain (n=5 biological replicates from three independent experiments). ***$P < 0.001$; **$P < 0.01$; n.s., not significant by one-way ANOVA. Error bars stand for standard error of the mean.

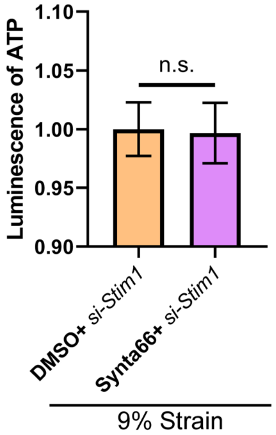

Fig. S23. Effect of SOCE activity repression in *Stim1*-partial knockdown tendon constructs. Luminescence measurement of ATP in 3D tendon constructs receiving 9% strain and transfected with *Stim1*-siRNA, and further treated with DMSO or Synta66 (10 μM) (*n*=5 biological replicates from three independent experiments). n.s., not significant by Student's *t*-test. Error bars stand for standard error of the mean.

Table S1. Primer Sets

Gene	Species	Primer sequence	
		Forward 5'->3'	Reverse 5'->3'
Stim1	Mouse	TGACAGGGACTGTACTGAAGATG	TATGCCGAGTCAAGAGAGGAG
Esyt-1	Mouse	GGGAGCTAACCCTCATCTGC	CGCCAATGATTCGTAATGGCTTT
Esyt-2	Mouse	CCGCCTGGGTTCATTTTCCA	GGTTTCTCGAAAGAGCTTCTCG
Esyt-3	Mouse	CAAACGCAACCGGAGGAAAG	GCCCCGGATCTTCTGAAGTTC
Tmem24	Mouse	CTTTCCGGGAGAACTGGCAA	CAAAGGCGATTTGGATGGAGC
Orp5	Mouse	ATGCAATGGATCTGACAAGGAAT	TTCTTCTCCTGCCGATAGTTCT
Kcnb1	Mouse	AGAAACACACAGCAATAGCGT	GTACTCCCGTGGAGACTCTTG
Kcnb2	Mouse	ACTGTAACACTCACGAGAGTCT	CTCCAGGATGTCGGTCGAAG
Gramd2	Mouse	TCGGCTCTATATCTCTCCCAAC	CCACAGGAATGACCACCTTGAT
β-Actin	Mouse	GTGACGTTGACATCCGTAAAGA	GCCGGACTCATCGTACTCC

Table S2. Search Strategy for Integrative Bioinformatic Analysis

Database	Search Formula
Gene Expression Omnibus	((((((mechanical[All Fields] AND stimulus[All Fields]) OR ("stress, mechanical"[MeSH Terms] OR mechanical stress[All Fields])) OR (mechanical[All Fields] AND ("sprains and strains"[MeSH Terms] OR strain[All Fields]))) OR (tensile[All Fields] AND ("sprains and strains"[MeSH Terms] OR strain[All Fields]))) OR mechanosensation[All Fields]) OR mechanotransduction[All Fields]) OR (mechanical[All Fields] AND loading[All Fields]) AND ("gse"[Filter] AND "Expression profiling by array"[Filter])

Table S3. Parameters in Computational Modelling

Cell Component	Morphological Parameters (y * x * z)	Physical Properties	Reference
Cell membrane	22.5 µm * 10 nm * 50 nm	Young's modulus: 10MPa<br>Poisson's Ratio 0.4[#]	(*129, 130*)
Nuclear	22.5 µm * 20 µm * 50 nm	Shear modulus: 50 Pa<br>Poisson's Ratio: 0.4[#]	(*131-134*)
Nuclear lamina	22.5 µm * 30 nm * 50 nm	Shear modulus: 50 Pa<br>Poisson's Ratio: 0.4[#]	(*131-134*)
Tether structure	150nm * 30 nm * 50 nm[##]	Young's modulus: 2 GPa<br>Poisson's Ratio: 0.4[#]	(*135*)
ER (total)	22.5 µm * 39.97µm * 50 nm	Young's modulus: 10 MPa<br>Poisson's Ratio: 0.4[#]	(*129, 130, 134*)
Mitochondrion	2 µm * 1 µm * 1 µm<br>(with membrane thickness of 10 nm)	Young's modulus: 10 MPa<br>Poisson's Ratio: 0.4[#]	(*129, 130, 136, 137*)
Lysosome	Sphere with a diameter of 500 nm<br>(with membrane thickness of 10 nm)	Young's modulus: 10 MPa<br>Poisson's Ratio: 0.4[#]	(*129, 130, 138, 139*)

[#]: For simplicity, fixed Poisson's Ratio was used.
[##]: Unless stated otherwise.

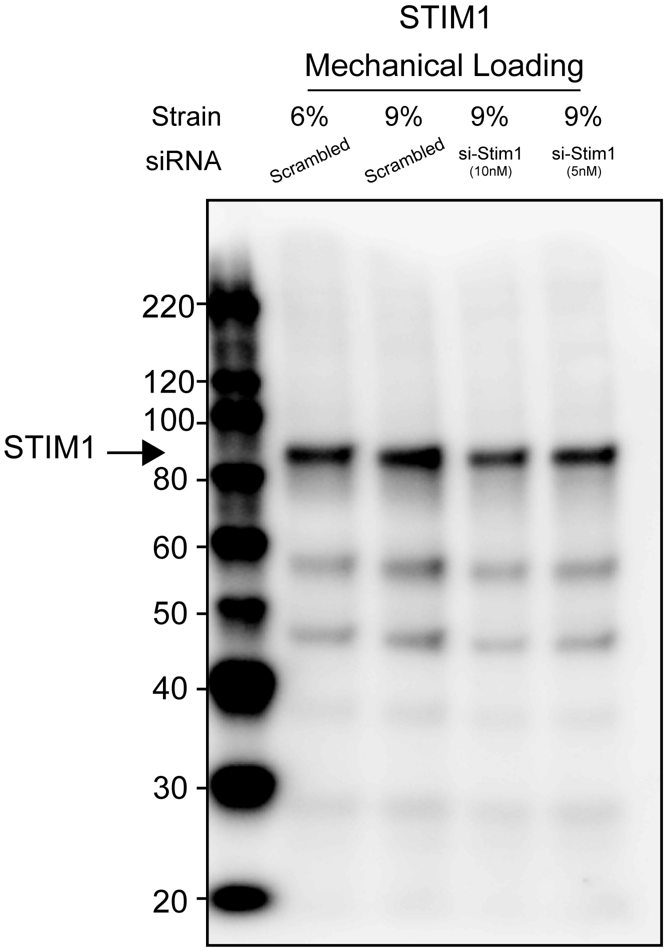
STIM1
Mechanical Loading
Strain
6%
9%
9%
9%
siRNA
Scrambled
Scrambled
si-Stim1
(10nM)
si-Stim1
(5nM)
220
120
100
80
60
50
40
30
20
STIM1

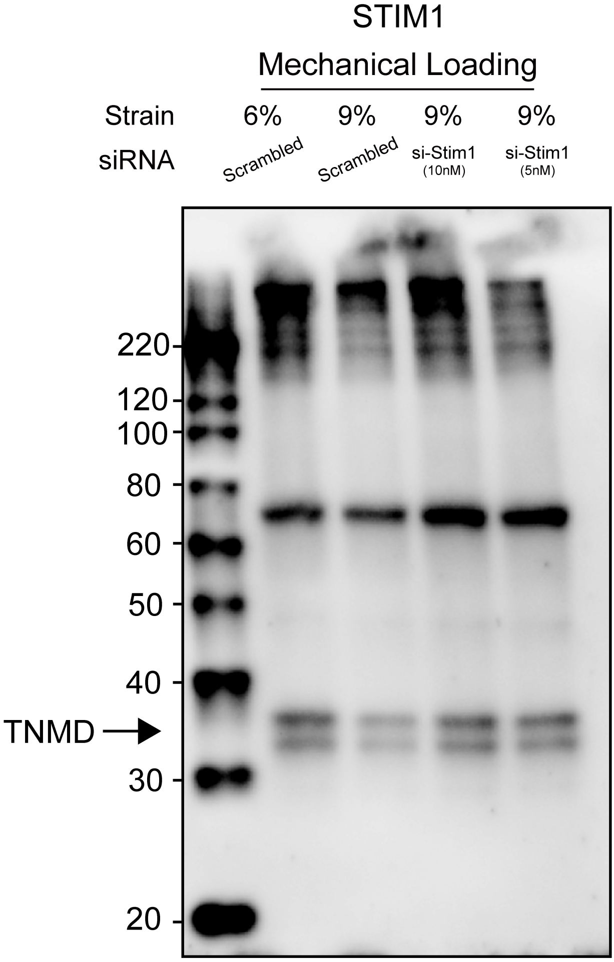
STIM1
Mechanical Loading
Strain
6%
9%
9%
9%
siRNA
Scrambled
Scrambled
si-Stim1
(10nM)
si-Stim1
(5nM)
220
120
100
80
60
50
40
30
20
TNMD

REFERENCES AND NOTES

1. J. Hu, S. Chen, W. Hu, S. Lü, M. Long, Mechanical point loading induces cortex stiffening and actin reorganization. *Biophys. J.* **117**, 1405–1418 (2019).

2. S. Mathieu, J. B. Manneville, Intracellular mechanics: Connecting rheology and mechanotransduction. *Curr. Opin. Cell Biol.* **56**, 34–44 (2019).

3. K. Tsujita, R. Satow, S. Asada, Y. Nakamura, L. Arnes, K. Sako, Y. Fujita, K. Fukami, T. Itoh, Homeostatic membrane tension constrains cancer cell dissemination by counteracting BAR protein assembly. *Nat. Commun.* **12**, 5930 (2021).

4. D. E. Jaalouk, J. Lammerding, Mechanotransduction gone awry. *Nat. Rev. Mol. Cell Biol.* **10**, 63–73 (2009).

5. S. Ma, S. Cahalan, G. LaMonte, N. D. Grubaugh, W. Zeng, S. E. Murthy, E. Paytas, R. Gamini, V. Lukacs, T. Whitwam, M. Loud, R. Lohia, L. Berry, S. M. Khan, C. J. Janse, M. Bandell, C. Schmedt, K. Wengelnik, A. I. Su, E. Honore, E. A. Winzeler, K. G. Andersen, A. Patapoutian, Common PIEZO1 allele in African populations causes RBC dehydration and attenuates plasmodium infection. *Cell* **173**, 443–455.e12 (2018).

6. E. Gracey, A. Burssens, I. Cambré, G. Schett, R. Lories, I. B. McInnes, H. Asahara, D. Elewaut, Tendon and ligament mechanical loading in the pathogenesis of inflammatory arthritis. *Nat. Rev. Rheumatol.* **16**, 193–207 (2020).

7. E. Cambria, M. F. Coughlin, M. A. Floryan, G. S. Offeddu, S. E. Shelton, R. D. Kamm, Linking cell mechanical memory and cancer metastasis. *Nat. Rev. Cancer* **24**, 216–228 (2024).

8. F. Rashid, W. Liu, Q. Wang, B. Ji, J. Irudayaraj, N. Wang, Mechanomemory in protein diffusivity of chromatin and nucleoplasm after force cessation. *Proc. Natl. Acad. Sci. U.S.A.* **120**, e2221432120 (2023).

9. J. L. Balestrini, S. Chaudhry, V. Sarrazy, A. Koehler, B. Hinz, The mechanical memory of lung myofibroblasts. *Integr. Biol. (Camb)* **4**, 410–421 (2012).

10. A. Saraswathibhatla, D. Indana, O. Chaudhuri, Cell-extracellular matrix mechanotransduction in 3D. *Nat. Rev. Mol. Cell Biol.* **24**, 495–516 (2023).

11. C. S. Janota, F. J. Calero-Cuenca, E. R. Gomes, The role of the cell nucleus in mechanotransduction. *Curr. Opin. Cell Biol.* **63**, 204–211 (2020).

12. F. A. Pennacchio, P. Nastały, A. Poli, P. Maiuri, Tailoring cellular function: The contribution of the nucleus in mechanotransduction. *Front. Bioeng. Biotechnol.* **8**, 596746 (2020).

13. F. Bertillot, Y. A. Miroshnikova, S. A. Wickström, SnapShot: Mechanotransduction in the nucleus. *Cell* **185**, 3638–3638.e1 (2022).

14. S. E. Szczesny, R. L. Mauck, The nuclear option: Evidence implicating the cell nucleus in mechanotransduction. *J. Biomech. Eng.* **139**, 0210061–02100616 (2017).

15. T. Panciera, L. Azzolin, M. Cordenonsi, S. Piccolo, Mechanobiology of YAP and TAZ in physiology and disease. *Nat. Rev. Mol. Cell Biol.* **18**, 758–770 (2017).

16. M. Wang, R. J. Kaufman, Protein misfolding in the endoplasmic reticulum as a conduit to human disease. *Nature* **529**, 326–335 (2016).

17. J. A. Olzmann, P. Carvalho, Dynamics and functions of lipid droplets. *Nat. Rev. Mol. Cell Biol.* **20**, 137–155 (2019).

18. D. S. Schwarz, M. D. Blower, The endoplasmic reticulum: Structure, function and response to cellular signaling. *Cell. Mol. Life Sci.* **73**, 79–94 (2016).

19. L. M. Westrate, J. E. Lee, W. A. Prinz, G. K. Voeltz, Form follows function: The importance of endoplasmic reticulum shape. *Annu. Rev. Biochem.* **84**, 791–811 (2015).

20. H. Wu, P. Carvalho, G. K. Voeltz, Here, there, and everywhere: The importance of ER membrane contact sites. *Science* **361**, eaan5835 (2018).

21. S. Phuyal, F. Baschieri, Endomembranes: Unsung heroes of mechanobiology? *Front. Bioeng. Biotechnol.* **8**, 597721 (2020).

22. C. Li, T. Qian, R. He, C. Wan, Y. Liu, H. Yu, Endoplasmic reticulum-plasma membrane contact sites: Regulators, mechanisms, and physiological functions. *Front. Cell Dev. Biol.* **9**, 627700 (2021).

23. P. Wang, R. Pleskot, J. Zang, J. Winkler, J. Wang, K. Yperman, T. Zhang, K. Wang, J. Gong, Y. Guan, C. Richardson, P. Duckney, M. Vandorpe, E. Mylle, J. Fiserova, D. Van Damme, P. J. Hussey, Plant AtEH/Pan1 proteins drive autophagosome formation at ER-PM contact sites with actin and endocytic machinery. *Nat. Commun.* **10**, 5132 (2019).

24. G. Csordás, D. Weaver, G. Hajnóczky, Endoplasmic reticulum-mitochondrial contactology: Structure and signaling functions. *Trends Cell Biol.* **28**, 523–540 (2018).

25. T. Di Mattia, C. Tomasetto, F. Alpy, Faraway, so close! Functions of endoplasmic reticulum-endosome contacts. *Biochim. Biophys. Acta Mol. Cell Biol. Lipids* **1865**, 158490 (2020).

26. G. Radovanović, S. Bohm, K. K. Peper, A. Arampatzis, K. Legerlotz, Evidence-based high-loading tendon exercise for 12 weeks leads to increased tendon stiffness and cross-sectional area in Achilles tendinopathy: A controlled clinical trial. *Sports Med. Open* **8**, 149 (2022).

27. S. Bohm, F. Mersmann, M. Tettke, M. Kraft, A. Arampatzis, Human Achilles tendon plasticity in response to cyclic strain: Effect of rate and duration. *J. Exp. Biol.* **217**, 4010–4017 (2014).

28. P. G. Smith, L. Deng, J. J. Fredberg, G. N. Maksym, Mechanical strain increases cell stiffness through cytoskeletal filament reorganization. *Am. J. Physiol. Lung Cell. Mol. Physiol.* **285**, L456–L463 (2003).

29. C. Guilluy, L. D. Osborne, L. Van Landeghem, L. Sharek, R. Superfine, R. Garcia-Mata, K. Burridge, Isolated nuclei adapt to force and reveal a mechanotransduction pathway in the nucleus. *Nat. Cell Biol.* **16**, 376–381 (2014).

30. K. N. Dahl, A. J. Ribeiro, J. Lammerding, Nuclear shape, mechanics, and mechanotransduction. *Circ. Res.* **102**, 1307–1318 (2008).

31. A. Ghisleni, N. C. Gauthier, Mechanotransduction through membrane tension: It's all about propagation? *Curr. Opin. Cell Biol.* **86**, 102294 (2023).

32. M. M. Kozlov, L. V. Chernomordik, Membrane tension and membrane fusion. *Curr. Opin. Struct. Biol.* **33**, 61–67 (2015).

33. X. Yang, C. Lin, X. Chen, S. Li, X. Li, B. Xiao, Structure deformation and curvature sensing of PIEZO1 in lipid membranes. *Nature* **604**, 377–383 (2022).

34. M. Simunovic, G. A. Voth, Membrane tension controls the assembly of curvature-generating proteins. *Nat. Commun.* **6**, 7219 (2015).

35. A. Diz-Muñoz, K. Thurley, S. Chintamen, S. J. Altschuler, L. F. Wu, D. A. Fletcher, O. D. Weiner, Membrane tension acts through PLD2 and mTORC2 to limit actin network assembly during neutrophil migration. *PLOS Biol.* **14**, e1002474 (2016).

36. K. Tsujita, T. Takenawa, T. Itoh, Feedback regulation between plasma membrane tension and membrane-bending proteins organizes cell polarity during leading edge formation. *Nat. Cell Biol.* **17**, 749–758 (2015).

37. T. Wang, Z. Lin, R. E. Day, B. Gardiner, E. Landao-Bassonga, J. Rubenson, T. B. Kirk, D. W. Smith, D. G. Lloyd, G. Hardisty, A. Wang, Q. Zheng, M. H. Zheng, Programmable mechanical stimulation influences tendon homeostasis in a bioreactor system. *Biotechnol. Bioeng.* **110**, 1495–1507 (2013).

38. J. H. Wang, Mechanobiology of tendon. *J. Biomech.* **39**, 1563–1582 (2006).

39. T. Wang, C. Thien, C. Wang, M. Ni, J. Gao, A. Wang, Q. Jiang, R. S. Tuan, Q. Zheng, M. H. Zheng, 3D uniaxial mechanical stimulation induces tenogenic differentiation of tendon-derived stem cells through a PI3K/AKT signaling pathway. *FASEB J.* **32**, 4804–4814 (2018).

40. A. Colom, E. Derivery, S. Soleimanpour, C. Tomba, M. D. Molin, N. Sakai, M. González-Gaitán, S. Matile, A. Roux, A fluorescent membrane tension probe. *Nat. Chem.* **10**, 1118–1125 (2018).

41. A. Goujon, A. Colom, K. Straková, V. Mercier, D. Mahecic, S. Manley, N. Sakai, A. Roux, S. Matile, Mechanosensitive fluorescent probes to image membrane tension in mitochondria, endoplasmic reticulum, and lysosomes. *J. Am. Chem. Soc.* **141**, 3380–3384 (2019).

42. B. Pontes, P. Monzo, L. Gole, A. L. Le Roux, A. J. Kosmalska, Z. Y. Tam, W. Luo, S. Kan, V. Viasnoff, P. Roca-Cusachs, L. Tucker-Kellogg, N. C. Gauthier, Membrane tension controls adhesion positioning at the leading edge of cells. *J. Cell Biol.* **216**, 2959–2977 (2017).

43. A.-L. Le Roux, X. Quiroga, N. Walani, M. Arroyo, P. Roca-Cusachs, The plasma membrane as a mechanochemical transducer. *Philos. Trans. R. Soc. London Ser. B Biol. Sci.* **374**, 20180221 (2019).

44. J. Xu, Y. Tseng, D. Wirtz, Strain hardening of actin filament networks. Regulation by the dynamic cross-linking protein α-actinin. *J. Biol. Chem.* **275**, 35886–35892 (2000).

45. M. M. Nava, Y. A. Miroshnikova, L. C. Biggs, D. B. Whitefield, F. Metge, J. Boucas, H. Vihinen, E. Jokitalo, X. Li, J. M. García Arcos, B. Hoffmann, R. Merkel, C. M. Niessen, K. N. Dahl, S. A. Wickström, Heterochromatin-driven nuclear softening protects the genome against mechanical stress-induced damage. *Cell* **181**, 800–817.e22 (2020).

46. C. Roffay, G. Molinard, K. Kim, M. Urbanska, V. Andrade, V. Barbarasa, P. Nowak, V. Mercier, J. García-Calvo, S. Matile, R. Loewith, A. Echard, J. Guck, M. Lenz, A. Roux, Passive coupling of membrane tension and cell volume during active response of cells to osmosis. *Proc. Natl. Acad. Sci. U.S.A.* **118**, e2103228118 (2021).

47. S. Schuck, W. A. Prinz, K. S. Thorn, C. Voss, P. Walter, Membrane expansion alleviates endoplasmic reticulum stress independently of the unfolded protein response. *J. Cell Biol.* **187**, 525–536 (2009).

48. S. A. Oakes, F. R. Papa, The role of endoplasmic reticulum stress in human pathology. *Annu. Rev. Pathol.* **10**, 173–194 (2015).

49. M. Calfon, H. Zeng, F. Urano, J. H. Till, S. R. Hubbard, H. P. Harding, S. G. Clark, D. Ron, IRE1 couples endoplasmic reticulum load to secretory capacity by processing the XBP-1 mRNA. *Nature* **415**, 92–96 (2002).

50. D. R. Beriault, G. H. Werstuck, Detection and quantification of endoplasmic reticulum stress in living cells using the fluorescent compound, Thioflavin T. *Biochim. Biophys. Acta* **1833**, 2293–2301 (2013).

51. C.-L. Chang, T.-S. Hsieh, T. T. Yang, K. G. Rothberg, D. B. Azizoglu, E. Volk, J.-C. Liao, J. Liou, Feedback regulation of receptor-induced Ca^{2+} signaling mediated by E-Syt1 and Nir2 at endoplasmic reticulum-plasma membrane junctions. *Cell Rep.* **5**, 813–825 (2013).

52. M. Besprozvannaya, E. Dickson, H. Li, K. S. Ginburg, D. M. Bers, J. Auwerx, J. Nunnari, GRAM domain proteins specialize functionally distinct ER-PM contact sites in human cells. *Elife* **7**, e31019 (2018).

53. F. Giordano, Y. Saheki, O. Idevall-Hagren, S. F. Colombo, M. Pirruccello, I. Milosevic, E. O. Gracheva, S. N. Bagriantsev, N. Borgese, P. De Camilli, PI (4,5)P_2-dependent and Ca^{2+}-regulated ER-PM interactions mediated by the extended synaptotagmins. *Cell* **153**, 1494–1509 (2013).

54. M. Kirmiz, S. Palacio, P. Thapa, A. N. King, J. T. Sack, J. S. Trimmer, Remodeling neuronal ER-PM junctions is a conserved nonconducting function of Kv2 plasma membrane ion channels. *Mol. Biol. Cell* **29**, 2410–2432 (2018).

55. J. Chung, F. Torta, K. Masai, L. Lucast, H. Czapla, L. B. Tanner, P. Narayanaswamy, M. R. Wenk, F. Nakatsu, P. De Camilli, PI4P/phosphatidylserine countertransport at ORP5- and ORP8-mediated ER-plasma membrane contacts. *Science* **349**, 428–432 (2015).

56. F. Vallese, C. Catoni, D. Cieri, L. Barazzuol, O. Ramirez, V. Calore, M. Bonora, F. Giamogante, P. Pinton, M. Brini, T. Calì, An expanded palette of improved SPLICS reporters detects multiple organelle contacts in vitro and in vivo. *Nat. Commun.* **11**, 6069 (2020).

57. J. A. Lees, M. Messa, E. W. Sun, H. Wheeler, F. Torta, M. R. Wenk, P. De Camilli, K. M. Reinisch, Lipid transport by TMEM24 at ER-plasma membrane contacts regulates pulsatile insulin secretion. *Science* **355**, eaah6171 (2017).

58. J. T. Parsons, A. R. Horwitz, M. A. Schwartz, Cell adhesion: Integrating cytoskeletal dynamics and cellular tension. *Nat. Rev. Mol. Cell Biol.* **11**, 633–643 (2010).

59. W. F. Lai, W. T. Wong, Roles of the actin cytoskeleton in aging and age-associated diseases. *Ageing Res. Rev.* **58**, 101021 (2020).

60. W. Lei, O. F. Omotade, K. R. Myers, J. Q. Zheng, Actin cytoskeleton in dendritic spine development and plasticity. *Curr. Opin. Neurobiol.* **39**, 86–92 (2016).

61. A. S. Moore, S. M. Coscia, C. L. Simpson, F. E. Ortega, E. C. Wait, J. M. Heddleston, J. J. Nirschl, C. J. Obara, P. Guedes-Dias, C. A. Boecker, T. L. Chew, J. A. Theriot, J. Lippincott-Schwartz, E. L. F. Holzbaur, Actin cables and comet tails organize mitochondrial networks in mitosis. *Nature* **591**, 659–664 (2021).

62. Y. Shen, N. B. Thillaiappan, C. W. Taylor, The store-operated Ca^{2+} entry complex comprises a small cluster of STIM1 associated with one Orai1 channel. *Proc. Natl. Acad. Sci. U.S.A.* **118**, e2010789118 (2021).

63. M. Yen, R. S. Lewis, Physiological CRAC channel activation and pore properties require STIM1 binding to all six Orai1 subunits. *J. Gen. Physiol.* **150**, 1373–1385 (2018).

64. L. Waldherr, A. Tiffner, D. Mishra, M. Sallinger, R. Schober, I. Frischauf, T. Schmidt, V. Handl, P. Sagmeister, M. Köckinger, I. Derler, M. Üçal, D. Bonhenry, S. Patz, R. Schindl, Blockage of store-operated Ca^{2+} influx by Synta66 is mediated by direct inhibition of the Ca^{2+} selective Orai1 Pore. *Cancers* **12**, 2876 (2020).

65. B. Sinha, D. Köster, R. Ruez, P. Gonnord, M. Bastiani, D. Abankwa, R. V. Stan, G. Butler-Browne, B. Vedie, L. Johannes, N. Morone, R. G. Parton, G. Raposo, P. Sens, C. Lamaze, P. Nassoy, Cells respond to mechanical stress by rapid disassembly of caveolae. *Cell* **144**, 402–413 (2011).

66. P. Rangamani, K. K. Mandadap, G. Oster, Protein-induced membrane curvature alters local membrane tension. *Biophys. J.* **107**, 751–762 (2014).

67. S. Yang, R. Zhou, C. Zhang, S. He, Z. Su, Mitochondria-associated endoplasmic reticulum membranes in the pathogenesis of type 2 diabetes mellitus. *Front. Cell Dev. Biol.* **8**, 571554 (2020).

68. G. A. Ngoh, K. N. Papanicolaou, K. Walsh, Loss of mitofusin 2 promotes endoplasmic reticulum stress. *J. Biol. Chem.* **287**, 20321–20332 (2012).

69. S. Cogliati, J. A. Enriquez, L. Scorrano, Mitochondrial cristae: Where beauty meets functionality. *Trends Biochem. Sci.* **41**, 261–273 (2016).

70. J. Gao, A. Qin, D. Liu, R. Ruan, Q. Wang, J. Yuan, T. S. Cheng, A. Filipovska, J. M. Papadimitriou, K. Dai, Q. Jiang, X. Gao, J. Q. Feng, H. Takayanagi, C. Zhang, M. H. Zheng, Endoplasmic reticulum mediates mitochondrial transfer within the osteocyte dendritic network. *Sci. Adv.* **5**, eaaw7215 (2019).

71. M. J. Barrera, S. Aguilera, I. Castro, P. Carvajal, D. Jara, C. Molina, S. González, M. J. González, Dysfunctional mitochondria as critical players in the inflammation of autoimmune diseases: Potential role in Sjögren's syndrome. *Autoimmun. Rev.* **20**, 102867 (2021).

72. A. P. Arruda, B. M. Pers, G. Parlakgül, E. Güney, K. Inouye, G. S. Hotamisligil, Chronic enrichment of hepatic endoplasmic reticulum-mitochondria contact leads to mitochondrial dysfunction in obesity. *Nat. Med.* **20**, 1427–1435 (2014).

73. V. I. Lushchak, M. Duszenko, D. V. Gospodaryov, O. Garaschuk, Oxidative stress and energy metabolism in the brain: Midlife as a turning point. *Antioxidants (Basel)* **10**, 1715 (2021).

74. M. Schieber, N. S. Chandel, ROS function in redox signaling and oxidative stress. *Curr. Biol.* **24**, R453–R462 (2014).

75. D. C. Liemburg-Apers, P. H. Willems, W. J. Koopman, S. Grefte, Interactions between mitochondrial reactive oxygen species and cellular glucose metabolism. *Arch. Toxicol.* **89**, 1209–1226 (2015).

76. C. Henríquez-Olguin, J. R. Knudsen, S. H. Raun, Z. Li, E. Dalbram, J. T. Treebak, L. Sylow, R. Holmdahl, E. A. Richter, E. Jaimovich, T. E. Jensen, Cytosolic ROS production by NADPH oxidase 2 regulates muscle glucose uptake during exercise. *Nat. Commun.* **10**, 4623 (2019).

77. S. A. Mookerjee, A. A. Gerencser, D. G. Nicholls, M. D. Brand, Quantifying intracellular rates of glycolytic and oxidative ATP production and consumption using extracellular flux measurements. *J. Biol. Chem.* **292**, 7189–7207 (2017).

78. A. Zalewska, I. Szarmach, M. Żendzian-Piotrowska, M. Maciejczyk, The effect of N-acetylcysteine on respiratory enzymes, ADP/ATP ratio, glutathione metabolism, and nitrosative stress in the salivary gland mitochondria of insulin resistant rats. *Nutrients* **12**, 458 (2020).

79. S. S. Cao, R. J. Kaufman, Endoplasmic reticulum stress and oxidative stress in cell fate decision and human disease. *Antioxid. Redox Signal.* **21**, 396–413 (2014).

80. J. Knupp, P. Arvan, A. Chang, Increased mitochondrial respiration promotes survival from endoplasmic reticulum stress. *Cell Death Differ.* **26**, 487–501 (2019).

81. P. Strzyz, ER stress boosts respiration. *Nat. Rev. Mol. Cell Biol.* **20**, 453 (2019).

82. I. N. Freitas, J. A. da Silva Jr., K. M. de Oliveira, B. Lourençoni Alves, T. Dos Reis Araújo, J. P. Camporez, E. M. Carneiro, A. P. Davel, Insights by which TUDCA is a potential therapy against adiposity. *Front. Endocrinol.* **14**, 1090039 (2023).

83. T. Barrett, S. E. Wilhite, P. Ledoux, C. Evangelista, I. F. Kim, M. Tomashevsky, K. A. Marshall, K. H. Phillippy, P. M. Sherman, M. Holko, A. Yefanov, H. Lee, N. Zhang, C. L. Robertson, N. Serova, S. Davis, A. Soboleva, NCBI GEO: Archive for functional genomics data sets—Update. *Nucleic Acids Res.* **41**, D991–D995 (2013).

84. D. T. Dang, Molecular approaches to protein dimerization: Opportunities for supramolecular chemistry. *Front. Chem.* **10**, 829312 (2022).

85. Y. Wu, C. Whiteus, C. S. Xu, K. J. Hayworth, R. J. Weinberg, H. F. Hess, P. De Camilli, Contacts between the endoplasmic reticulum and other membranes in neurons. *Proc. Natl. Acad. Sci. U.S.A.* **114**, E4859–E4867 (2017).

86. Y. F. Chen, W. T. Chiu, Y. T. Chen, P. Y. Lin, H. J. Huang, C. Y. Chou, H. C. Chang, M. J. Tang, M. R. Shen, Calcium store sensor stromal-interaction molecule 1-dependent signaling plays an important role in cervical cancer growth, migration, and angiogenesis. *Proc. Natl. Acad. Sci. U.S.A.* **108**, 15225–15230 (2011).

87. L. Orci, M. Ravazzola, M. Le Coadic, W. W. Shen, N. Demaurex, P. Cosson, STIM1-induced precortical and cortical subdomains of the endoplasmic reticulum. *Proc. Natl. Acad. Sci. U.S.A.* **106**, 19358–19362 (2009).

88. C. C. Price, J. Mathur, J. D. Boerckel, A. Pathak, V. B. Shenoy, Dynamic self-reinforcement of gene expression determines acquisition of cellular mechanical memory. *Biophys. J.* **120**, 5074–5089 (2021).

89. K. M. Schmoller, P. Fernández, R. C. Arevalo, D. L. Blair, A. R. Bausch, Cyclic hardening in bundled actin networks. *Nat. Commun.* **1**, 134 (2010).

90. M. Luciano, C. Tomba, A. Roux, S. Gabriele, How multiscale curvature couples forces to cellular functions. *Nat. Rev. Phys.* **6**, 246–268 (2024).

91. W. Li, X. Yu, F. Xie, B. Zhang, S. Shao, C. Geng, A. U. R. Aziz, X. Liao, B. Liu, A membrane-bound biosensor visualizes shear stress-induced inhomogeneous alteration of cell membrane tension. *iScience* **7**, 180–190 (2018).

92. D. Lachowski, C. Matellan, S. Gopal, E. Cortes, B. K. Robinson, A. Saiani, A. F. Miller, M. M. Stevens, A. E. Del Río Hernández, Substrate stiffness-driven membrane tension modulates vesicular trafficking via caveolin-1. *ACS Nano* **16**, 4322–4337 (2022).

93. S. Lehoux, Y. Castier, A. Tedgui, Molecular mechanisms of the vascular responses to haemodynamic forces. *J. Intern. Med.* **259**, 381–392 (2006).

94. N. L. Millar, K. G. Silbernagel, K. Thorborg, P. D. Kirwan, L. M. Galatz, G. D. Abrams, G. A. C. Murrell, I. B. McInnes, S. A. Rodeo, Tendinopathy. *Nat. Rev. Dis. Primers* **7**, 1 (2021).

95. R. V. Durvasula, S. J. Shankland, Mechanical strain increases SPARC levels in podocytes: Implications for glomerulosclerosis. *Am. J. Physiol. Renal Physiol.* **289**, F577–F584 (2005).

96. B. Fortune, Pulling and tugging on the retina: Mechanical impact of glaucoma beyond the optic nerve head. *Invest. Ophthalmol. Vis. Sci.* **60**, 26–35 (2019).

97. R. M. Adam, S. H. Eaton, C. Estrada, A. Nimgaonkar, S. C. Shih, L. E. Smith, I. S. Kohane, D. Bägli, M. R. Freeman, Mechanical stretch is a highly selective regulator of gene expression in human bladder smooth muscle cells. *Physiol. Genomics* **20**, 36–44 (2004).

98. J. He, B. Fang, S. Shan, Y. Xie, C. Wang, Y. Zhang, X. Zhang, Q. Li, Mechanical stretch promotes hypertrophic scar formation through mechanically activated cation channel Piezo1. *Cell Death Dis.* **12**, 226 (2021).

99. S. K. Sahetya, E. C. Goligher, R. G. Brower, Fifty years of research in ARDS. Setting positive end-expiratory pressure in acute respiratory distress syndrome. *Am. J. Respir. Crit. Care Med.* **195**, 1429–1438 (2017).

100. S.-J. Gwak, S. H. Bhang, I.-K. Kim, S.-S. Kim, S.-W. Cho, O. Jeon, K. J. Yoo, A. J. Putnam, B.-S. Kim, The effect of cyclic strain on embryonic stem cell-derived cardiomyocytes. *Biomaterials* **29**, 844–856 (2008).

101. M. K. Hayward, J. M. Muncie, V. M. Weaver, Tissue mechanics in stem cell fate, development, and cancer. *Dev. Cell* **56**, 1833–1847 (2021).

102. J. A. Hawley, M. Hargreaves, M. J. Joyner, J. R. Zierath, Integrative biology of exercise. *Cell* **159**, 738–749 (2014).

103. J. Lammerding, Mechanics of the nucleus. *Compr. Physiol.* **1**, 783–807 (2011).

104. K. Ohashi, S. Fujiwara, K. Mizuno, Roles of the cytoskeleton, cell adhesion and rho signalling in mechanosensing and mechanotransduction. *J. Biochem.* **161**, 245–254 (2017).

105. G. Halder, S. Dupont, S. Piccolo, Transduction of mechanical and cytoskeletal cues by YAP and TAZ. *Nat. Rev. Mol. Cell Biol.* **13**, 591–600 (2012).

106. P. Biswal, M. R. Sahu, M. H. Ahmad, A. C. Mondal, The interplay between hippo signaling and mitochondrial metabolism: Implications for cellular homeostasis and disease. *Mitochondrion* **76**, 101885 (2024).

107. J. Li, B. Hou, S. Tumova, K. Muraki, A. Bruns, M. J. Ludlow, A. Sedo, A. J. Hyman, L. M. Keown, R. S. Young, N. Y. Yuldasheva, Y. Majeed, L. A. Wilson, B. Rode, M. A. Bailey, H. R. Kim, Z. Fu, D. A. L. Carter, J. Bilton, H. Imrie, P. Ajuh, T. N. Dear, R. M. Cubbon, M. T. Kearney, K. R. Prasad, P. C. Evans, J. F. X. Ainscough, D. J. Beech, Piezo1 integration of vascular architecture with physiological force. *Nature* **515**, 279–282 (2014).

108. C. J. O'Conor, H. A. Leddy, H. C. Benefield, W. B. Liedtke, F. Guilak, TRPV4-mediated mechanotransduction regulates the metabolic response of chondrocytes to dynamic loading. *Proc. Natl. Acad. Sci. U.S.A.* **111**, 1316–1321 (2014).

109. S. Wang, W. Li, P. Zhang, Z. Wang, X. Ma, C. Liu, K. Vasilev, L. Zhang, X. Zhou, L. Liu, J. Hayball, S. Dong, Y. Li, Y. Gao, L. Cheng, Y. Zhao, Mechanical overloading induces GPX4-regulated chondrocyte ferroptosis in osteoarthritis via Piezo1 channel facilitated calcium influx. *J. Adv. Res.* **41**, 63–75 (2022).

110. L. L. Walkon, J. O. Strubbe-Rivera, J. N. Bazil, Calcium overload and mitochondrial metabolism. *Biomolecules* **12**, 1891 (2022).

111. O. Lityagina, G. Dobreva, The LINC between mechanical forces and chromatin. *Front. Physiol.* **12**, 710809 (2021).

112. N. Koushki, A. Ghagre, L. K. Srivastava, C. Molter, A. J. Ehrlicher, Nuclear compression regulates YAP spatiotemporal fluctuations in living cells. *Proc. Natl. Acad. Sci. U.S.A.* **120**, e2301285120 (2023).

113. A. Elosegui-Artola, I. Andreu, A. E. M. Beedle, A. Lezamiz, M. Uroz, A. J. Kosmalska, R. Oria, J. Z. Kechagia, P. Rico-Lastres, A. L. Le Roux, C. M. Shanahan, X. Trepat, D.

Navajas, S. Garcia-Manyes, P. Roca-Cusachs, Force triggers YAP nuclear entry by regulating transport across nuclear pores. *Cell* **171**, 1397–1410.e14 (2017).

114. C. P. Lusk, M. C. King, Nuclear pore complexes feel the strain. *Mol. Cell* **81**, 4962–4963 (2021).

115. F. Salehi, H. Behboudi, G. Kavoosi, S. K. Ardestani, Oxidative DNA damage induced by ROS-modulating agents with the ability to target DNA: A comparison of the biological characteristics of citrus pectin and apple pectin. *Sci. Rep.* **8**, 13902 (2018).

116. Y. Song, Z. Zhao, L. Xu, P. Huang, J. Gao, J. Li, X. Wang, Y. Zhou, J. Wang, W. Zhao, L. Wang, C. Zheng, B. Gao, L. Jiang, K. Liu, Y. Guo, X. Yao, L. Duan, Using an ER-specific optogenetic mechanostimulator to understand the mechanosensitivity of the endoplasmic reticulum. *Dev. Cell* **59**, 1396–1409.e5 (2024).

117. B. J. McHugh, R. Buttery, Y. Lad, S. Banks, C. Haslett, T. Sethi, Integrin activation by Fam38A uses a novel mechanism of R-Ras targeting to the endoplasmic reticulum. *J. Cell Sci.* **123**, 51–61 (2010).

118. N. S. Lee, C. W. Yoon, Q. Wang, S. Moon, K. M. Koo, H. Jung, R. Chen, L. Jiang, G. Lu, A. Fernandez, R. H. Chow, A. C. Weitz, P. M. Salvaterra, F. Pinaud, K. K. Shung, Focused ultrasound stimulates ER localized mechanosensitive PANNEXIN-1 to mediate intracellular calcium release in invasive cancer cells. *Front. Cell Dev. Biol.* **8**, 504 (2020).

119. Z. Chen, P. Chen, R. Ruan, M. Zheng, In vitro 3D mechanical stimulation to tendon-derived stem cells by bioreactor. *Methods Mol. Biol.* **2436**, 135–144 (2022).

120. A. R. English, G. K. Voeltz, Rab10 GTPase regulates ER dynamics and morphology. *Nat. Cell Biol.* **15**, 169–178 (2013).

121. T. Calì, M. Brini, Quantification of organelle contact sites by split-GFP-based contact site sensors (SPLICS) in living cells. *Nat. Protoc.* **16**, 5287–5308 (2021).

122. G. S. Bird, W. I. DeHaven, J. T. Smyth, J. W. Putney Jr., Methods for studying store-operated calcium entry. *Methods* **46**, 204–212 (2008).

123. M. C. Ludikhuize, M. Meerlo, B. M. T. Burgering, M. J. Rodríguez Colman, Protocol to profile the bioenergetics of organoids using Seahorse. *STAR Protoc.* **2**, 100386 (2021).

124. M. E. Ritchie, B. Phipson, D. Wu, Y. Hu, C. W. Law, W. Shi, G. K. Smyth, limma powers differential expression analyses for RNA-sequencing and microarray studies. *Nucleic Acids Res.* **43**, e47 (2015).

125. R. Kolde, S. Laur, P. Adler, J. Vilo, Robust rank aggregation for gene list integration and meta-analysis. *Bioinformatics* **28**, 573–580 (2012).

126. J. Nixon-Abell, C. J. Obara, A. V. Weigel, D. Li, W. R. Legant, C. S. Xu, H. A. Pasolli, K. Harvey, H. F. Hess, E. Betzig, C. Blackstone, J. Lippincott-Schwartz, Increased spatiotemporal resolution reveals highly dynamic dense tubular matrices in the peripheral ER. *Science* **354**, aaf3928 (2016).

127. B. Zucker, M. M. Kozlov, Mechanism of shaping membrane nanostructures of endoplasmic reticulum. *Proc. Natl. Acad. Sci. U.S.A.* **119**, e2116142119 (2022).

128. G. H. C. Chung, M. Lorvellec, P. Gissen, F. Pichaud, J. J. Burden, C. J. Stefan, The ultrastructural organization of endoplasmic reticulum-plasma membrane contacts is conserved in epithelial cells. *Mol. Biol. Cell* **33**, ar113 (2022).

129. T. Jadidi, H. Seyyed-Allaei, M. R. R. Tabar, A. Mashaghi, Poisson's ratio and young's modulus of lipid bilayers in different phases. *Front. Bioeng. Biotechnol.* **2**, 8 (2014).

130. A. Janshoff, C. Steinem, Mechanics of lipid bilayers: What do we learn from pore-spanning membranes? *Biochim. Biophys. Acta* **1853**, 2977–2983 (2015).

131. P. Panorchan, B. W. Schafer, D. Wirtz, Y. Tseng, Nuclear envelope breakdown requires overcoming the mechanical integrity of the nuclear lamina. *J. Biol. Chem.* **279**, 43462–43467 (2004).

132. C. R. Ethier, C. A. Simmons, *Introductory Biomechanics: From Cells to Organisms* (Cambridge Univ. Press, 2007).

133. A. Vaziri, H. Lee, M. R. Kaazempur Mofrad, Deformation of the cell nucleus under indentation: Mechanics and mechanisms. *J. Mater. Res.* **21**, 2126–2135 (2006).

134. J. K. Kim, A. Louhghalam, G. Lee, B. W. Schafer, D. Wirtz, D. H. Kim, Nuclear lamin A/C harnesses the perinuclear apical actin cables to protect nuclear morphology. *Nat. Commun.* **8**, 2123 (2017).

135. M. Bathe, A finite element framework for computation of protein normal modes and mechanical response. *Proteins* **70**, 1595–1609 (2008).

136. G. W. Berbusse, L. C. Woods, B. P. Vohra, K. Naylor, Mitochondrial dynamics decrease prior to axon degeneration induced by vincristine and are partially rescued by overexpressed cytNmnat1. *Front. Cell. Neurosci.* **10**, 179 (2016).

137. G. Perkins, C. Renken, M. E. Martone, S. J. Young, M. Ellisman, T. Frey, Electron tomography of neuronal mitochondria: Three-dimensional structure and organization of cristae and membrane contacts. *J. Struct. Biol.* **119**, 260–272 (1997).

138. H. Xu, D. Ren, Lysosomal physiology. *Annu. Rev. Physiol.* **77**, 57–80 (2015).

139. S. Y. Zhu, R. Q. Yao, Y. X. Li, P. Y. Zhao, C. Ren, X. H. Du, Y. M. Yao, Lysosomal quality control of cell fate: A novel therapeutic target for human diseases. *Cell Death Dis.* **11**, 817 (2020).
